# Transcriptomic analysis of resistant and susceptible banana corms in response to infection by *Fusarium oxysporum* f. sp. *cubense* tropical race 4

**DOI:** 10.1038/s41598-019-44637-x

**Published:** 2019-06-03

**Authors:** Lei Zhang, Alberto Cenci, Mathieu Rouard, Dong Zhang, Yunyue Wang, Weihua Tang, Si-Jun Zheng

**Affiliations:** 1 State Key Laboratory for Conservation and Utilization of Bio-Resources in Yunnan, Ministry of Education Key Laboratory of Agriculture Biodiversity for Plant Disease Management, Yunnan Agricultural University, Kunming, 650201 China; 2 Agricultural Environment and Resources Institute, Yunnan Academy of Agricultural Sciences, Kunming, 650205 China; 3 Bioversity International, 2238 Beijing Road, Kunming, 650205 Yunnan China; 4 Bioversity International, Parc Scientifique Agropolis II, 34397 Montpellier, Cedex 5 France; 5 National Key Laboratory of Plant Molecular Genetics, CAS Center for Excellence in Molecular Plant Sciences, Institute of Plant Physiology and Ecology, Shanghai Institutes for Biological Sciences, Chinese Academy of Sciences, Shanghai, 200032 China; 6 Dehong Agricultural Technology Extension Center, Mangshi, 678400 China

**Keywords:** Gene expression analysis, Pathogens, Transcriptomics

## Abstract

Fusarium wilt disease, caused by *Fusarium oxysporum* f. sp. *cubense*, especially by tropical race 4 (*Foc* TR4), is threatening the global banana industry. *Musa acuminata* Pahang, a wild diploid banana that displays strong resistance to *Foc* TR4, holds great potential to understand the underlying resistance mechanisms. Microscopic examination reports that, in a wounding inoculation system, the *Foc* TR4 infection processes in roots of Pahang (resistant) and a triploid cultivar Brazilian (susceptible) were similar by 7 days post inoculation (dpi), but significant differences were observed in corms of both genotypes at 14 dpi. We compare transcriptomic responses in the corms of Pahang and Brazilian, and show that Pahang exhibited constitutive defense responses before *Foc* TR4 infection and inducible defense responses prior to Brazilian at the initial *Foc* TR4 infection stage. Most key enzymatic genes in the phenylalanine metabolism pathway were up-regulated in Brazilian, suggesting that lignin and phytotoxin may be triggered during later stages of *Foc* TR4 infection. This study unravels a few potential resistance candidate genes whose expression patterns were assessed by RT-qPCR assay and improves our understanding the defense mechanisms of Pahang response to *Foc* TR4.

## Introduction

Fusarium wilt disease, caused by *Fusarium oxysporum* f. sp. *cubense* (*Foc*), is a destructive soil-borne banana disease which is threatening the global banana industry^1,2^. *Foc* has been divided into 4 races and 24 vegetative compatibility groups (VCGs) according to pathogenicity on reference host cultivars and vegetative compatibility, respectively^3–5^. Tropical race 4 (TR4, VCG 01213/16) is of particular concern because the Cavendish subgroup of cultivars, the dominant export commodity, succumbs to this specific strain^2,6–8^. So far, limited options are available to manage Fusarium wilt of banana, due largely to the long-term existence of chlamydospores in soil and pathogens in host xylem vessels^9^. Deploying resistant cultivars is still the most effective and economical measure at present, however, seedless and parthenocarpic characters hamper the application of traditional breeding methods in banana genetic improvement^10,11^. To date, few resistant cultivars with acceptable agronomic traits could be used for production^9^. Global understanding of the plant-pathogen interaction at molecular levels would help in developing strategies to combat Fusarium wilt disease^12^.

Plants and pathogens have formed complex relationships of competitive interactions in the long-term evolutionary process. Plants recognize pathogen-associated molecular patterns (PAMPs) by utilizing pathogen recognition receptors (PRRs) to activate PAMP-triggered immunity (PTI)^13^. Once a pathogen has acquired the capacity to overcome primary defenses by secreting effectors, the plant detects it through resistant (R) proteins to activate effector-triggered immunity (ETI)^14,15^. After *Foc* infection, bananas also undergo a series of changes in physiology, biochemistry and molecular biology, such as accumulating H_2_O_2_ sharply in the root cells^16^; strengthening root cell walls through accumulation of lignin and phenolics^17–19^; secreting root exudates to inhibit spore germination and hyphal growth^20^; activating salicylic acid (SA) or jasmonic acid (JA) metabolism and signal transduction^21,22^; up-regulating cell-death genes like *MusaBAG1*^23^ and β-1,3-glucanase genes^24^ to inhibit the growth of the pathogen. Although these studies focused on the banana-*Foc* interaction, the molecular mechanisms underlying resistance to Fusarium wilt remained unclear.

The whole genome of DH-Pahang, a doubled-haploid of a wild *Musa acuminata* Pahang, was sequenced and 36,542 protein-coding genes were identified^25,26^, which provides an important step to explore the genetic basis of traits. The sequence analysis highlighted three whole genome duplications in the *Musa* genome history that resulted in at least one third of the annotated genes being present in multiple copies (paralogs)^25^. Furthermore, Pahang expressed strong resistance to *Foc* TR4 in the greenhouse and field trials^25,27,28^. Therefore, more attention is needed on unraveling the mechanisms at the molecular level in this wild *Foc* TR4 resistant genotype. Next-generation sequencing (NGS) has already been used for various applications, especially for gene expression analysis in a large number of plant species^29^. Previous studies were conducted on Cavendish banana roots infected with *Foc* TR4 by using suppression subtractive hybridization (SSH)^30^ or NGS^31–35^. Except for Li *et al*.^31^ who extracted RNA from the mixture of roots, pseudostems, leaves, fruits and flowers, other research groups^30,32–35^ used only roots as starting materials. However, roots should not be the only tissue to investigate because Fusarium wilt of banana is a typical vascular disease. Although the pathogen usually causes infection in the roots of susceptible and resistant banana cultivars, but infection generally progresses into vascularized portions of the corm only in susceptible cultivars^36^.

In the present study, we selected the highly resistant *Musa acuminata* Pahang and the highly susceptible Cavendish cultivar Brazilian to compare. We observed that although *Foc* TR4 invaded both banana genotypes through wounded roots, much severe disease symptoms appeared in above-ground parts of the susceptible genotype than resistant genotype, particularly in corms. Therefore, we sampled their corms at various infection stages to construct cDNA libraries, then sequenced using Illumina HiSeq™. Comparative transcriptome analysis showed that Pahang exhibited constitutive defense responses before inoculation and inducible defense responses prior to Brazilian at the initial *Foc* TR4 infection stage. RT-qPCR assays further indicated the high reliability of RNA-seq data.

## Results

### *Foc* TR4 infection process

**Figure 1:**
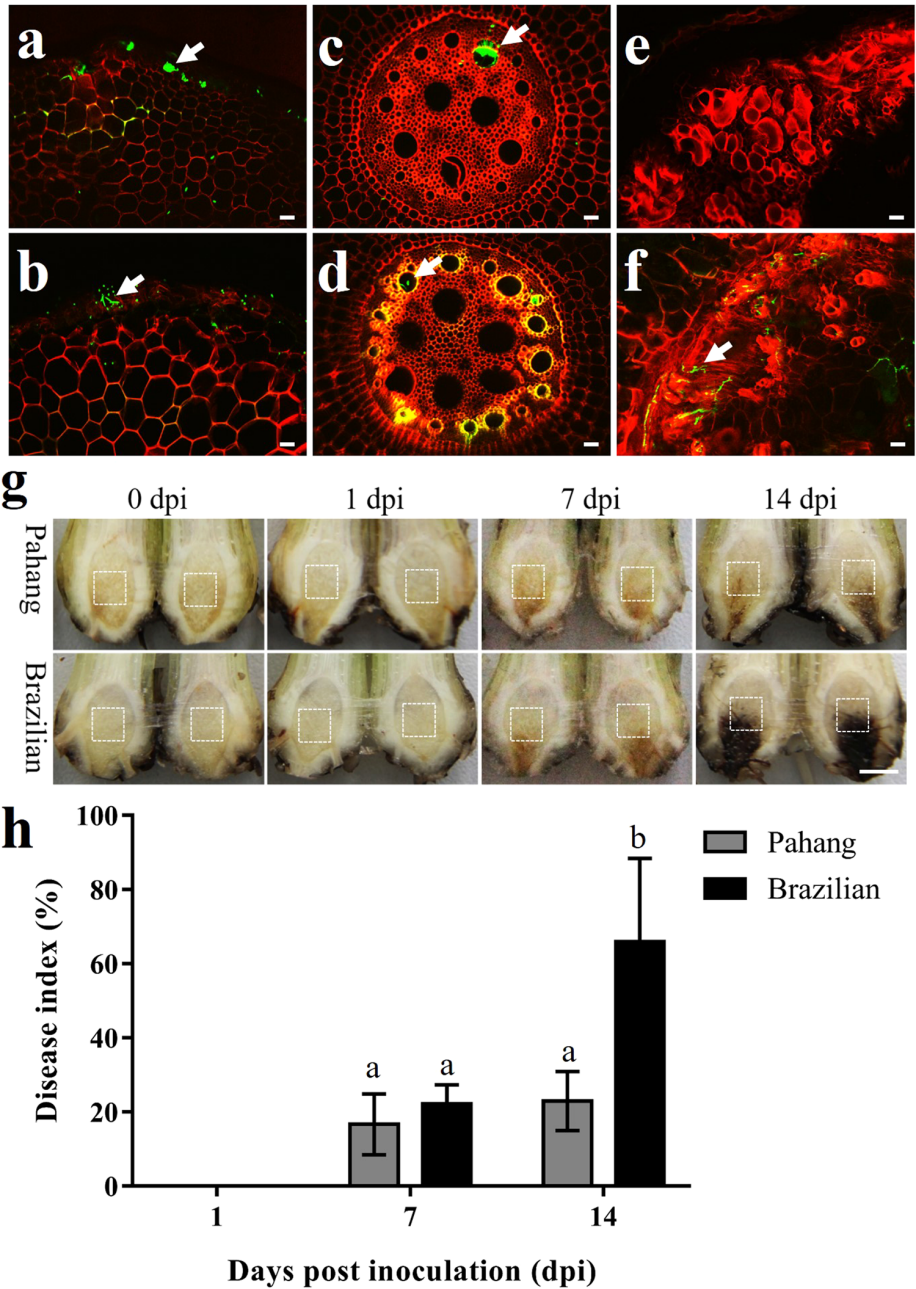
Comparison of *Foc* TR4 infection processes and disease index of Pahang and Brazilian. At 1 dpi, hyphae and spores adhere to the root epidermis of Pahang (**a**) and Brazilian (**b**). At 7 dpi, hyphae extend upward along root vascular bundles to corms of Pahang (**c**) and Brazilian (**d**). At 14 dpi, no hypha is found in corm central cylinder tissues of Pahang (**e**), but lots of hyphae grow in that of Brazilian (**f**). Bars = 50 μm. Arrows indicate *Foc* TR4 hyphae. (**g**) The symptoms of Fusarium wilt in banana corms. White boxes represent the location of sampling at different dpi. Bars = 5 mm. (**h**) Disease index survey with the Pahang and Brazilian in the greenhouse experiments. Nine plants per genotype were used in each treatment. Error bars represent standard deviation of the mean with four independent experiments. Significant differences were calculated using Tukey HSD. Different letters mean significant differences (P < 0.01).

Pahang and Brazilian wounded roots were inoculated with AmCyan-*Foc* TR4 to monitor the infection processes. At 1 dpi, hyphae and spores adhered to root epidermis of Pahang (Fig. 1a) and Brazilian (Fig. 1b). At 7 dpi, hyphae extended upward along root vascular bundles to corms of Pahang (Fig. 1c) and Brazilian (Fig. 1d). At 14 dpi, no hypha was found in corm central cylinder tissues of Pahang (Fig. 1e), but a lot of hyphae grew in that of Brazilian (Fig. 1f). Additionally, the lesion size in corms (Fig. 1g) and disease index (Fig. 1h) of Pahang was significantly less than that of Brazilian at 14 dpi. It suggested that in a wounding inoculation system, the infection processes in roots of Pahang and Brazilian were similar by 7 dpi, but significant differences occurred in corms of both banana genotypes at 14 dpi.

### Transcriptomic analysis

The cDNA libraries constructed from Pahang and Brazilian were sequenced with Illumina HiSeq^TM^ through strict data quality control. After removing adapter, ploy-N and low-quality reads, we obtained an average of 48,900,401 and 49,626,721 filtered reads in Pahang and Brazilian libraries, respectively. The Q20 of both were more than 95% (Supplementary Table S2). Among which, 82.70% (Pahang) and 65.07% (Brazilian) of reads were uniquely mapped to the banana reference genome sequence v2 (Supplementary Table S3). An average of 24,468 and 23,978 unigenes expressed in Pahang and Brazilian libraries, respectively, with FPKM > 1 was used as a threshold to determine gene expression (Supplementary Table S4). Principal component analysis (PCA) showed that the untreated and all treated (inoculated and mock) samples of Pahang and Brazilian were separately aggregated, indicating significant differences existed in gene expression profiles between different varieties and treatments (Supplementary Fig. S2).

**Figure 2:**
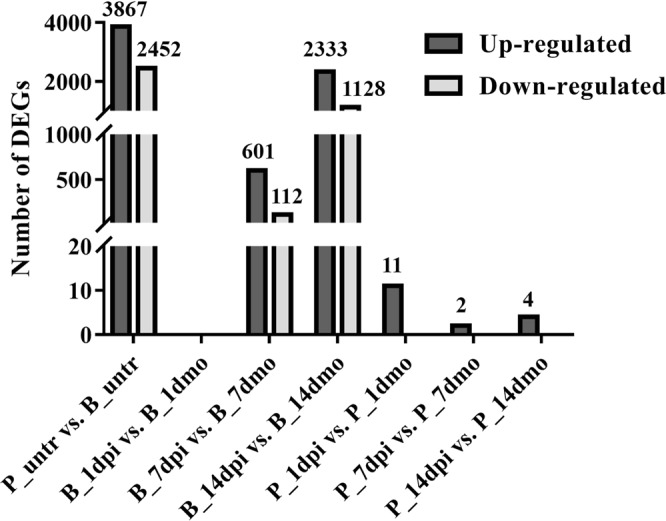
Number of differentially expressed genes (DEGs).

A total of 6,319 differentially expressed genes (DEGs, >15% of total genes) were identified in P_untr vs. B_untr, among which 3,867 DEGs (Pahang^+^) expressed at higher levels in Pahang than those in Brazilian and 2,452 DEGs (Brazilian^+^) expressed at lower levels in Pahang than those in Brazilian. No DEG was identified in B_1dpi vs. B_1dmo; 713 DEGs were identified in B_7dpi vs. B_7dmo, among which 601 DEGs were up-regulated and 112 DEGs were down-regulated; 3,461 DEGs were identified in B_14dpi vs. B_14dmo, of which 2,333 DEGs were up-regulated and 1,128 DEGs were down-regulated. Interestingly, there were 11 up-regulated DEGs in P_1dpi vs. P_1dmo, 2 up-regulated DEGs in P_7dpi vs. P_7dmo and 4 up-regulated DEGs in P_14dpi vs. P_14dmo, but no down-regulated DEGs were identified (Fig. 2).

### Functional category of DEGs

DEGs were further annotated by Gene Ontology (GO) and Kyoto Encyclopedia of Genes and Genomes (KEGG) functional enrichment.

**Figure 3:**
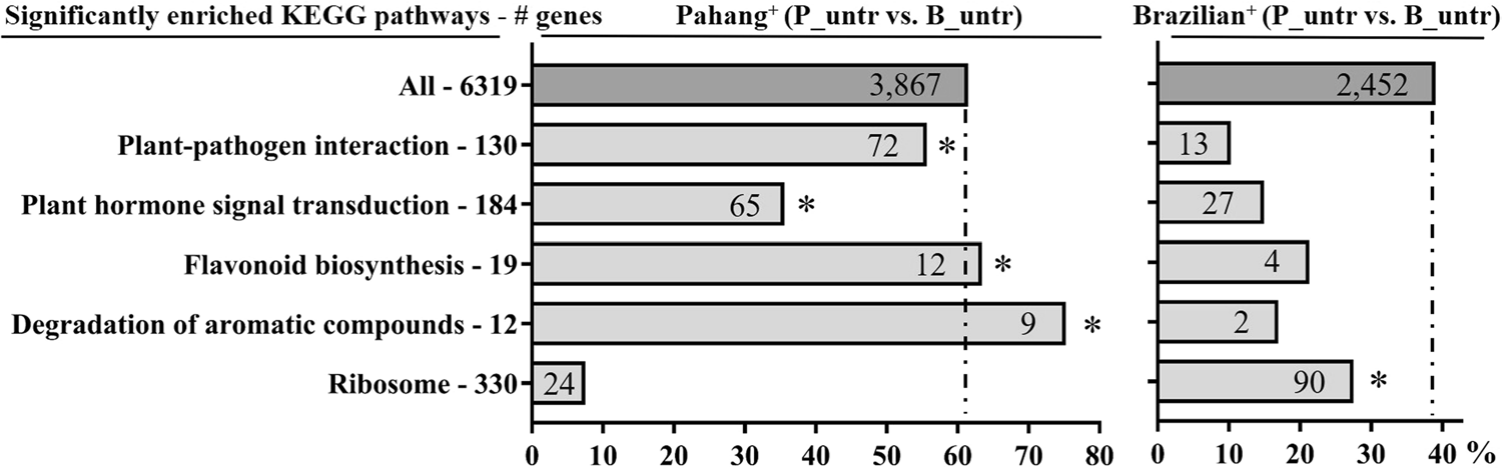
All significantly enriched KEGG pathways of DEGs in Pahang compared to Brazilian before inoculation (P_untr vs. B_untr). Vertical dashed lines indicate the percentage of Pahang^+^ and Brazilian^+^ DEGs in all DEGs by comparing Pahang with Brazilian before inoculation (P_untr vs. B_untr). The background number (#) of genes in each pathway is indicated on the left. The input number of DEGs within each pathway is indicated on the horizontal bars. Asterisks indicate significantly enriched pathways of DEGs in Pahang^+^ or Brazilian^+^ (*Corrected P-Value < 0.05).

DEGs identified in P_untr vs. B_untr. 2,602 DEGs (67.29% of all 3,867 Pahang^+^ DEGs) were annotated to the GO database under three groups including biological process (BP), cellular component (CC) and molecular function (MF) (Supplementary Fig. S3a). Among BP, “regulation of nucleobase-containing compound metabolic process”, “regulation of RNA biosynthetic process” and “regulation of RNA metabolic process” were the dominant terms. Among MF, “nucleic acid binding transcription factor activity”, “transcription factor activity, sequence-specific DNA binding”, “sequence-specific DNA binding” were significantly enriched terms. No DEGs were significantly enriched in CC. The Pahang^+^ DEGs were further analyzed using KEGG functional enrichment (Fig. 3, Supplementary Dataset). There were four significantly enriched pathways including “plant-pathogen interaction”, “plant hormone signal transduction”, “flavonoid biosynthesis” and “degradation of aromatic compounds”. 1,771 DEGs (72.23% of all 2,452 Brazilian^+^ DEGs) were annotated to the GO database (Supplementary Fig. S3b). The top three significantly enriched terms in BP were “peptide metabolic process”, “cellular amide metabolic process” and “translation”; in CC “ribosome”, “ribonucleoprotein complex” and “intracellular non-membrane-bounded organelle”; in MF “structural constituent of ribosome”, “structural molecule activity” and “oxidoreductase activity”. KEGG analysis showed that Brazilian^+^ DEGs were only significantly enriched in “ribosome” pathway (Fig. 3). The list of 85 DEGs corresponding to the most enriched pathway (i.e. plant-pathogen interaction) was mostly composed of WRKY and TIFY transcription factors, putative disease resistance genes (i.e. RPM1-like, RPP8-like) as well as calcium-binding protein, calcium-dependent protein kinase and calmodulin-like Protein (Supplementary Table 5).

**Figure 4:**
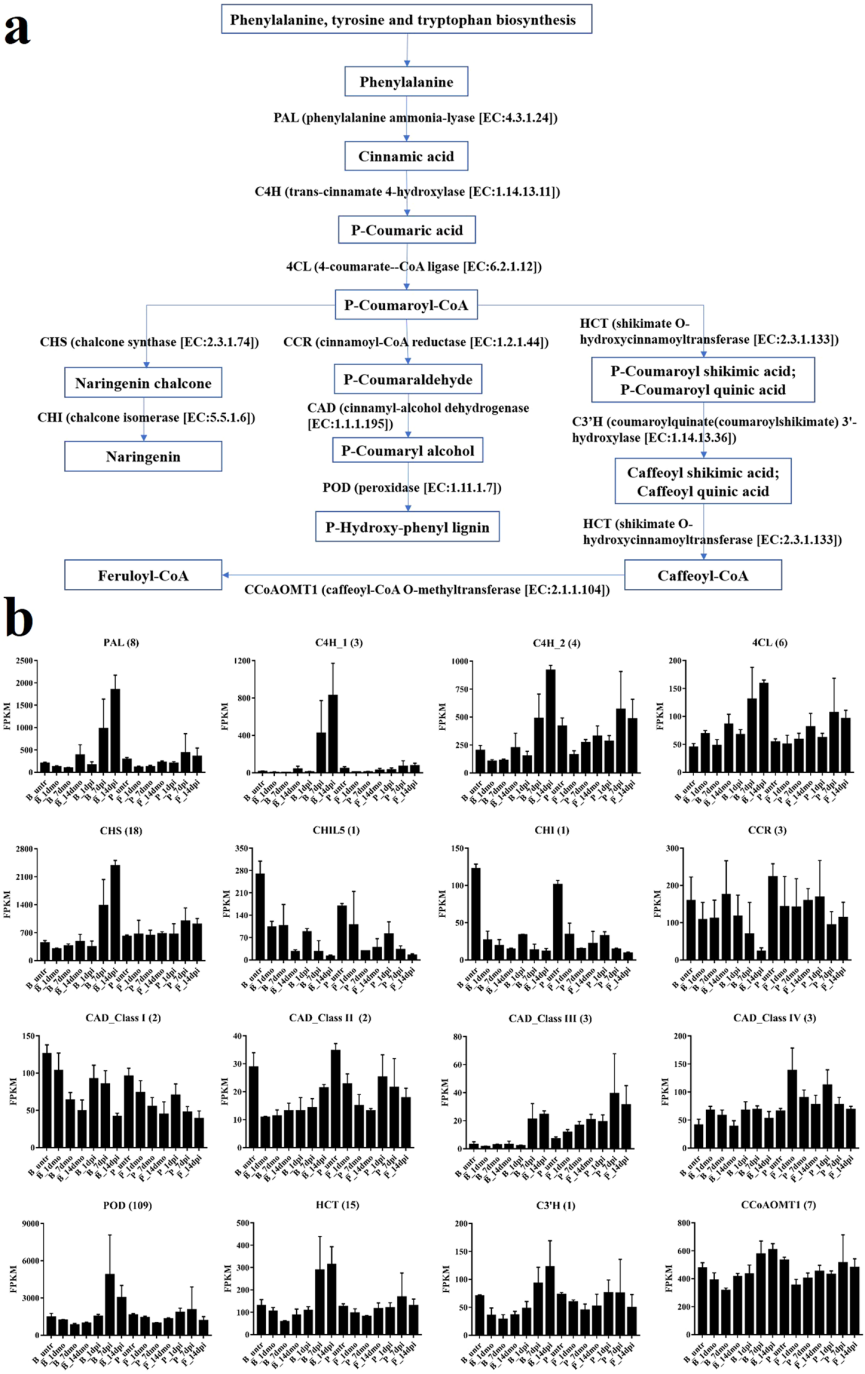
The expression pattern of genes coding for phenylalanine biosynthesis related enzymes. (**a**) Phenylalanine biosynthesis pathway (KEGG database). (**b**) Global expression profile of genes coding for phenylalanine biosynthesis related enzymes. Each bar represents cumulative gene expression (FPKM) by library for Pahang and Brazilian for the different time points. Error bars represent standard deviation of the mean with three independent biological replicates. The number in brackets represent the number of genes.

DEGs identified in B_dpi vs. B_dmo. A total of 2,775 DEGs (76.30% of all 3,637 DEGs) were assigned to the GO database (Supplementary Fig. S4a). The most significantly enriched terms were “oxidation-reduction process” in BP and “oxidoreductase activity” in MF. Additionally, these DEGs were further assigned to KEGG pathways (Supplementary Fig. S4b). The most significantly enriched pathways were “phenylpropanoid biosynthesis”, followed by “phenylalanine metabolism”, “biosynthesis of secondary metabolites”, “starch and sucrose metabolism”, “taurine and hypotaurine metabolism” and “plant hormone signal transduction”. We further analyzed the expression of DEGs involved in the phenylpropanoid biosynthesis pathway (Fig. 4a). To examine in depth the implication of the phenylpropanoid biosynthesis pathway in response to the *Foc* TR4 infection, all genes included in the KEGG database for 11 enzymes involved in this pathway were analyzed for their expression. All enzymes but coumaroylquinate (coumaroylshikimate) 3′-hydroxylase (C3′H) are coded by more than one gene in *Musa* genome. When considered together, the global expression of most of enzymes, such as phenylalanine ammonia-lyase (PAL), trans-cinnamate 4-hydroxylase (C4H), 4-coumarate-CoA ligase (4CL), chalcone synthase (CHS), peroxidase (POD), shikimate O-hydroxycinnamoyltransferase (HCT) and C3′H, appears up-regulated in B_7dpi and B_14dpi when compared with B_7dmo and B_14dmo, respectively **(**Fig. 4b**)**. The ten genes coding for cinnamyl-alcohol dehydrogenase (CAD) were subdivided in the four classes suggested by Eudes *et al*.^37^ and each class analyzed separately. Only class III genes resulted up-regulated in B_7dpi and B_14dpi **(**Supplementary Tables S6 and S7).

DEGs identified in P_dpi vs. P_dmo. A total of 17 DEGs were identified. Of which 16 DEGs’ sequences were aligned to GO and Swiss-prot database by BLAST (Supplementary Table S8). These DEGs included 3 pathogenesis-related protein 1-like (PR1), 2 chitinase (CHIT), 2 aspartic proteinase (ASP), 1 GDSL lipase (GLIP), 1 peroxidase (POD), 1 polyphenol oxidase (PPO), 1 putative glucuronosyltransferase PGSIP8, 1 phenylpropanoylacetyl-CoA synthase-like, 1 3-ketoacyl-CoA synthase, 1 sphinganine C(4)-monooxygenase 1-like, 1 cytochrome P450, 1 WRKY transcription factor and 1 uncharacterized gene.

### Paralogous Inclusive Expression analysis (PIE)

**Figure 5:**
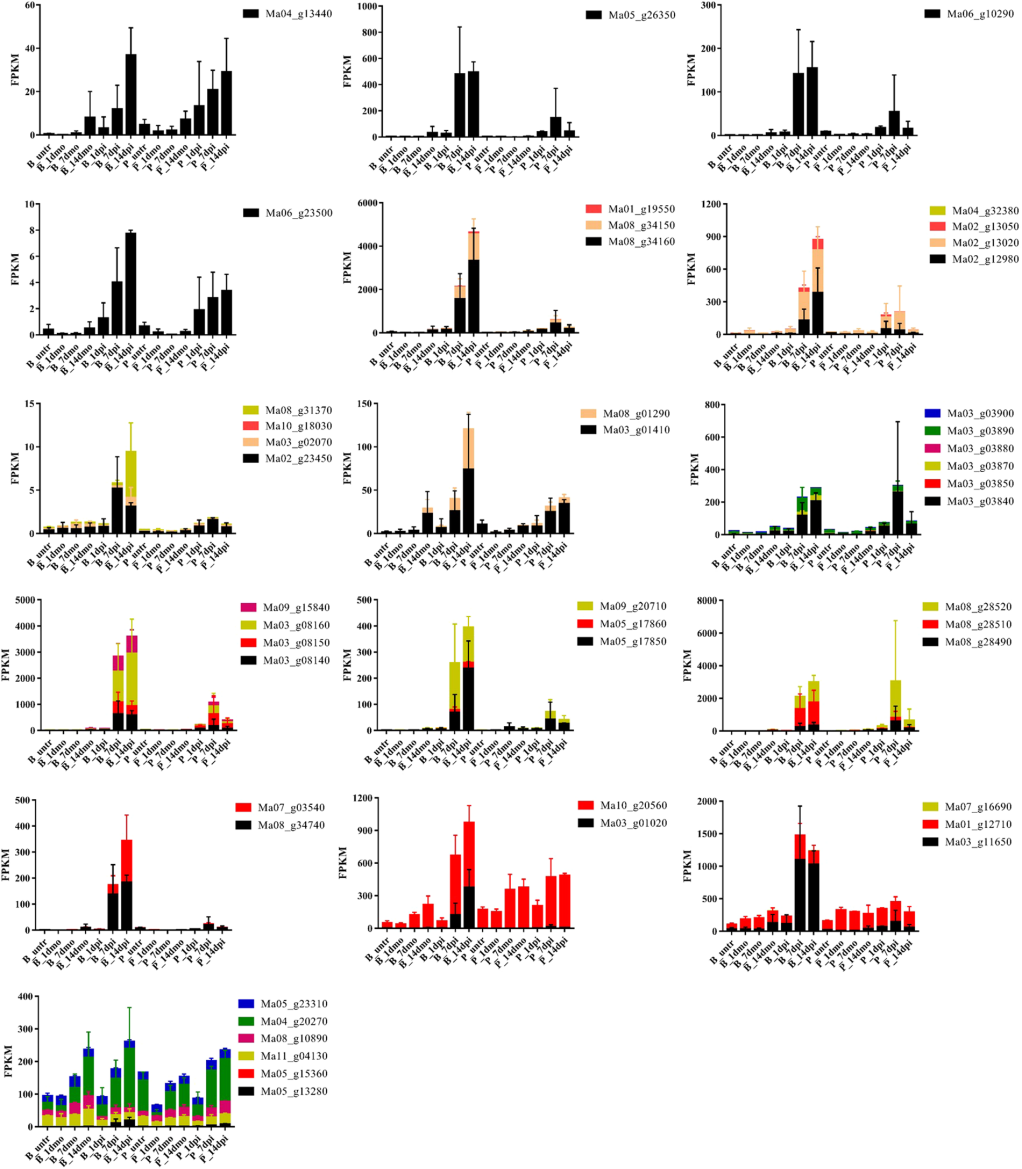
Paralogous Inclusive Expression analyses of the 17 DEGs. Each bar represents cumulative paralogous gene expression (FPKM) by library for Pahang and Brazilian for the different time points. DEGs expression are identified in black while respective paralogs are represented with other colors. Locus names are indicated in the legend. Error bars represent standard deviation of the mean with three independent biological replicates.

Due to ancient whole genome duplications, a relevant number of *Musa* genes are present in multiple copies with expected functional redundancy. Consequently, paralogous genes associated to DEGs were searched to assess their impact when considering gene expression additive effects. Among the 17 DEGs identified in Pahang under *Foc* TR4 infection, 4 DEGs (Ma04_g13440, Ma05_g26350, Ma06_g10290 and Ma06_g23500) have no paralog in the *Musa* genome. Their gene expression was not submitted to further analysis (Fig. 5). For the other 13 DEGs, gene expression of the paralog was considered to calculate the global gene expression. Since two of remaining 13 DEGs (Ma08_g34150 and Ma08_g34160) were paralogs, likely originated by a tandem duplication, they were analyzed together. As a result of the paralogous inclusive expression (PIE) analyses, all but three of the proteins coded by DEGs confirmed the global up-regulation of respective paralogous genes in Pahang to be due to *Foc* TR4 infection. For 3 genes, the PIE analysis highlighted inconsistencies with the expression of the respective DEGs. Consequently, the synthesis of the enzymes coded by these three DEGs and their respective paralogs was no longer considered as influenced by the infection of *Foc* TR4 in Pahang. The gene Ma10_g20560, paralog of Ma03_g01020 had a comparable level of expression in infected and mock Pahang corms and its expression level is higher than Ma03_g01020; Ma01_g12710, paralogs of Ma03_g11650 had comparable expression in infected and mock Pahang corms and its expression level was higher than Ma03_g11650; the five Ma05_g13280 paralogs had dramatically higher level of expression than Ma05_g13280 and the PIE analysis showed increased expression in infected and mock samples in both Pahang and Brazilian. It is worth to underline that all the proteins coded by these genes but Ma05_g13280 are highly up-regulated in Brazilian infected samples at 7 and/or 14 dpi.

### Assessment of the RNA-seq data

**Figure 6:**
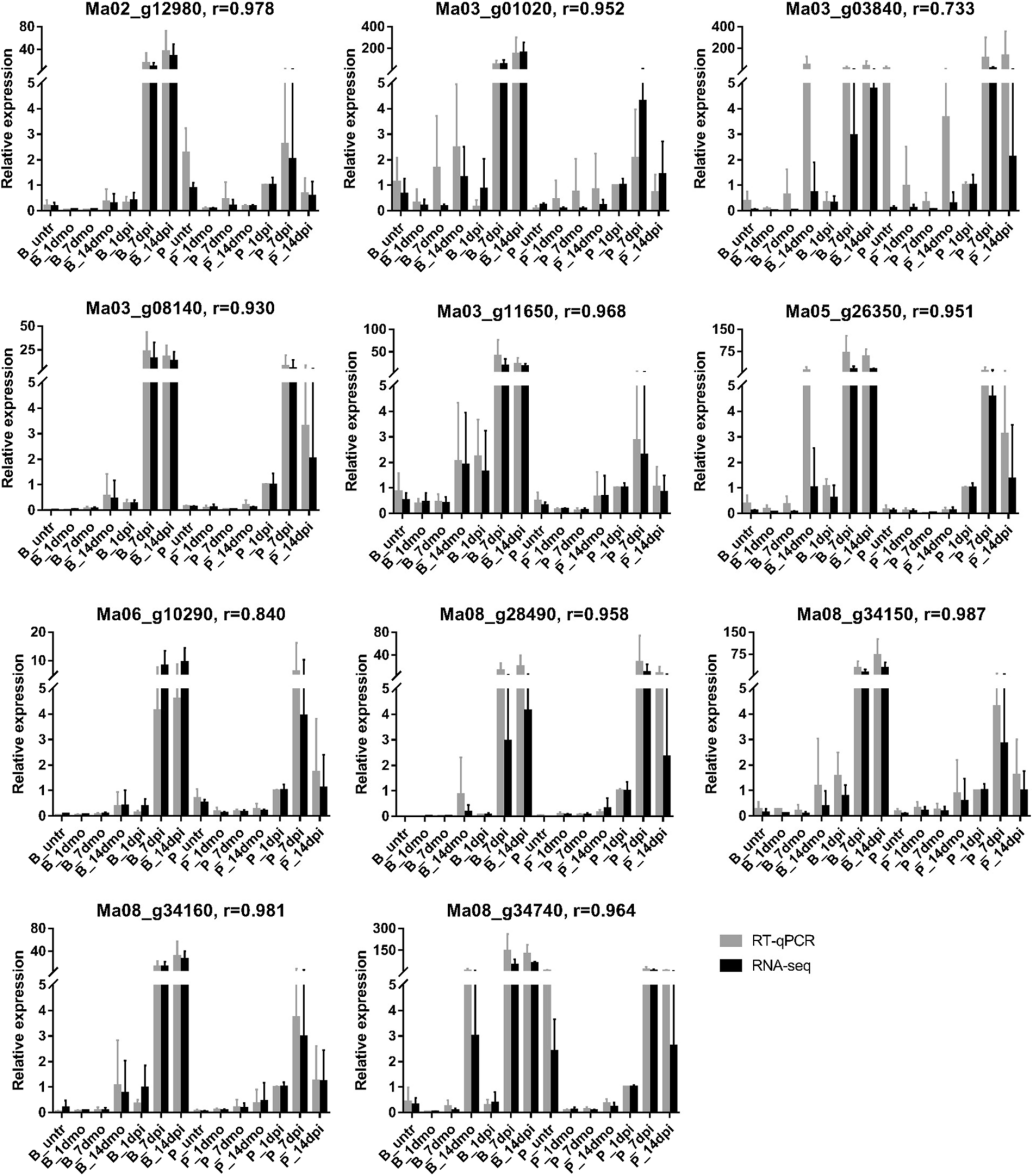
Assessment of RNA-seq data by RT-qPCR. 11 DEGs identified in Pahang response to *Foc* TR4 at 1 dpi compared with that of mock (P_1dpi vs. P_1dmo) are selected for assessment using RT-qPCR and showed high correlation coefficient (r) with RNA-seq data. Error bars represent standard deviation of the mean with three independent biological replicates.

A set of 11 DEGs identified in P_1dpi vs. P_1dmo which were supposed to play key roles in response to *Foc* TR4 at early infection stage were selected to assess using the RT-qPCR. These DEGs included 3 PR1 (Ma02_g08140, Ma08_g34150, Ma08_g34160), 2 ASP (Ma03_g03840, Ma03_g11650), 1 POD (Ma06_g10290), 1 PPO (Ma08_g34740), 1 GLIP (Ma02_g12980), 1 CHIT (Ma08_g28490), 1 cytochrome P450 (Ma05_g26350) and 1 phenylpropanoylacetyl-CoA synthase gene (Ma03_g01020). The RT-qPCR results for 81.8% (9/11) genes showed highly consistent with RNA-seq data; 90.9% genes (10/11) were consistent; only 9.1% genes (1/11) were inconsistent (Fig. 6), which suggested high reliability of RNA-seq results in our study.

## Discussion

### Sampling strategy and importance of corm tissues

Transcriptome sequencing has been performed to understand the differential response to *Foc* TR4 between resistant and susceptible banana genotypes, but how the resistant banana genotypes avoided infection have not been conclusive. In the previous NGS analyses^32,34,35^, the untreated banana roots harvested as controls were compared with inoculated root samples to identify DEGs, which was difficult to exclude changes in gene expression caused by wounds. Additionally, it was difficult to sample the roots at later infection stages, because the wounding roots rotted after 7 dpi. In the present study, we sampled banana corms to construct cDNA libraries, and identified potential resistant genes by comparing inoculated samples with mock samples at various infection stages. We focused on the response to *Foc* TR4 of corm tissues, which link roots to the pseudostem and leaves, rather than the roots because significant differential symptoms were displayed in corms due to *Foc* TR4 infection, and this is the first report where the corm is analysed during the banana-*Foc* TR4 interaction.

### Functional annotation of DEGs

During unchallenged conditions (P_untr vs. B_untr), Pahang, compared to Brazilian, showed a higher number of over-expressed genes than under-expressed genes with 3,867 DEGs for Pahang^+^ and 2,452 DEGs for Brazilian^+^. Pahang^+^ DEGs were significantly enriched in the resistance-related pathways, implying Pahang exhibited constitutive defense responses before inoculation. The result was similar to a previous study which reported that defense was built up in a resistant coffee accession before leaf miner (*Leucoptera coffella*) attack^38^. Among the 3,867 DEGs, it is worth mentioning that the most enriched KEGG pathway belonged to plant-pathogen interaction and was composed on 7 WRKY transcription factors known to play an important role in biotic stress resistance^39–41^. Interestingly, 12 out of the 51 TIFY transcription factors contained in the banana genome were also differentially expressed. Besides, 4 putative disease resistance genes were also present.

In B_dpi vs. B_dmo, the number of DEGs increased after inoculation, suggesting that the expression profiles of Brazilian were enhanced in the *Foc* TR4 infection process, and the most significantly enriched GO terms were “oxidation-reduction process” in BP and “oxidoreductase activity” in MF, indicating that the antioxidant system was activated in Brazilian at later infection stages. This result was also shown in tobacco after nematode infection^42^. Additionally, the most significantly enriched KEGG pathways were “phenylpropanoid biosynthesis” and “phenylalanine metabolism”, which was in agreement with previous study^32^. It indicates that these pathways participate in the regulation of responses to *Foc* TR4 attack (Supplementary Dataset). We decided to focus on the pathway starting from Phenylalanine (an amino acid used for protein synthesis) and, consequently, supposed to be abundant in cells and we followed three pathways toward lignin and toward two molecules where the upregulation appears to finish (Feruloyl CoA and Naringenin). Interestingly, all the enzymes upstream the synthesis of Caffeoyl Co-A (CCoA) are up-regulated. The CCoA is the substrate for the Caffeoyl-CoA O-methyltransferase (CCoAOMT) conferring quantitative resistance to multiple pathogens^43^. Even if the CCoAOMT is not up-regulated, the increase of substrate (CCoA) should modify the chemical equilibrium of the reaction catalyzed by CCoAOMT and increase the production of Feruloyl CoA that was suggested to strengthen the cell wall in response to pathogen aggression^44^.

In P_1dpi vs. P_1dmo, eight of the proteins coded by DEGs were up-regulated, and most proteins involved in defense against pathogens such as PR1, POD, PPO and CHIT, whereas no DEG was identified in B_1 dpi vs. B_1dmo. It suggested that Pahang exhibited inducible defense responses prior to Brazilian at the initial *Foc* TR4 infection stage, as suggested in previous studies^16,19,34^. Bai *et al*.^34^ proposed that the highly resistant cultivar ‘Yueyoukang 1’ (Cavendish subgroup) triggered a much faster defense response against *Foc* TR4 infection than ‘Brazilian’; Van den Berg *et al*.^19^ and Li *et al*.^16^ reported that tolerance genotypes of banana to Fusarium wilt would be associated with early up-regulation of cell wall-strengthening genes and early H_2_O_2_ accumulation in the roots.

In P_dpi vs. P_dmo, most DEGs expression levels were higher in B_7dpi than B_1dpi, and in P_7dpi than P_1dpi, suggesting both banana genotypes enhanced defense response in the *Foc* TR4 infection process. Interestingly, the expression levels of most DEGs were significantly higher in B_7dpi than P_7dpi, and in B_14dpi than P_14dpi. There might be two main reasons. First, Pahang suppressed the fungal growth in its corm due to the constitutive and inducible defense responses, whereas Brazilian did not respond promptly at the early infection stage, resulting in massive mycelia in its corm, which induced higher expression of massive genes, but it was too late to prevent *Foc* TR4 infection. Second, Pahang might technically lack the receptor genes corresponding to pathogen effectors so the effector-triggered susceptibility was not activated. It might be also another case here as very few genes were differentially expressed in the wild resistant genotype, while in susceptible genotype several thousand genes were differentially expressed. It is the next step that we try to find important susceptibility genes in those differentially expressed in susceptible genotype or candidate resistant genes having higher/lower basal expressed in mock inoculated plants.

### Genome wide analysis of DEGs

The *Musa* genome underwent three whole genome duplications in its evolution after lineage divergence from another monocot commelinidae^25^ that resulted in the presence of a high proportion of genes in multiple copies^45^. Recent paralogs are supposed to code for enzymes with similar functions. A genome wide analysis was performed to find paralogs of the most interesting DEGs identified in the comparative analyses. Even if paralog inclusive expression analysis is more conservative because it takes into account the expression of genes not identified as differentially expressed, its results confirmed the synthesized increase of almost all the enzymes coded by genes differentially expressed in the Pahang comparison between infected and mock samples and of the enzymes involved in the phenylalanine metabolism pathway in the Brazilian cultivar. Interestingly, lignin metabolism and production (phenylpropanoid metabolism-related genes, such as PAL, POD and CCoAOMT) were also proved to be had important roles in response to multiple pathogens^43,46^. The activities of enzyme and relative expressions of genes encoding PAL, C4H and CCoAOMT which are key intermediates dedicated to the biosynthesis of lignin monomers were positively induced in oil palm plantlets infected by *Ganoderma boninense*, except for CAD^47^. The expression of HCT was up-regulated in upland cotton whereas PAL, 4CL, CAD, CCoAOMT and COMT were up-regulated in sea-island cotton. Almost no DEGs involved in phenylpropanoid metabolism pathway were identified in these cotton genotypes when they were inoculated with *Verticillium dahliae*^48^. These results were similar with our findings. C4H and C3’H are the near the beginning of lignin biosynthetic pathway and their expression down-regulated resulted in lowering overall lignin content in Eucalyptus trees^49^. Plant can produce more flavonoid pathway products like naringenin with the help of CHS to protect it against biotic and abiotic stress^50^. Therefore, analysis of the expressions of phenylpropanoid metabolism-related genes are meaningful to elucidate the molecular mechanisms of banana response to *Foc* TR4.

### Identification of the genes involved in defense against *Foc* TR4

Recently, Dale *et al*.^7^ created a transgenic resistant banana by overexpressing one nucleotide-binding/leucine-rich repeat (NB-LRR) gene named RGA2 (resistance gene analog 2) from a resistant wild banana, and the transgenic plant was shown to be completely immune to *Foc* TR4 when planted in a heavily contaminated field. In addition, they found that the highly susceptible cultivar ‘Grande Naine’ already contained three low expression levels of the RGA2 homologues. Interestingly, in our RNA-seq data, both genotypes also included one weakly expressed RGA2-like gene (Ma03_g09130), showing 99.97% identity with RGA2. The expression levels of RGA2-like gene were very low, and showed no significant differential after *Foc* TR4 infection in Pahang and Brazilian. However, 4 putative disease resistance genes (Ma04_g35240, Ma07_g21730, Ma07_g21750 and Ma08_g31160), involved in plant-pathogen interaction, were threefold higher in Pahang than in Brazilian during unchallenged conditions, indicating their roles in constitutive defense mechanism.

In the present study, several proteins coded by DEGs identified in Pahang may play key roles in the banana-*Foc* TR4 interaction, including PR1, CHIT, ASP, GLIP, POD, PPO, cytochrome P450 and WRKY transcription factors.

Pathogenesis-related proteins have been divided into 17 families of induced proteins, among which PR1 is a dominant group induced by pathogens or salicylic acid, and is usually used as a marker of pathogen-induced systemic acquired resistance (SAR) conferring on plants the enhanced defensive state^51,52^. Li *et al*.^30^ also found the PR1 transcript level increased in each genotype in response to *Foc* TR4 attack. In line with these studies, we found 2 PR1 (Ma03_g08140, Ma08_g34150/Ma08_g34160) that maintained up-regulation in both banana genotypes during *Foc* TR4 infection. Of these, Ma03_g08140 has 99% identity with PR1-3 (KF582558.1), whose expression is significantly increased in the resistant cultivar response to *Foc* TR4^21^.

Chitin is an important component of fungal cell walls. Chitinase, an enzyme induced by the plant hormone ethylene or pathogen attack, has been shown to inhibit fungal growth *in vitro*^53^ and accumulate around fungal hyphae *in vivo*^54^. It has been demonstrated that overexpression of chitinase in transgenic plants reduces the fungal pathogen damage^55^. Subramaniam *et al*.^56^ reported that overexpressed chitinase in transgenic banana is correlated with *Foc* race 1 resistance. In the present study, 2 CHIT (Ma05_g17850, Ma08_g28490) were activated in both banana genotypes by *Foc* TR4, especially at later infection stages, suggesting the possible involvement of chitinase in response to *Foc* TR4.

Aspartic proteinases are one of four super-families of proteolytic enzymes widely distributed in varied living organisms, and they play key roles in the regulation of biological processes including defense response to pathogen attack. For example, Xia *et al*.^57^ and Prasad *et al*.^58^ reported that overexpression of CDR1, which encodes aspartate proteases, leads to enhanced resistance against bacterial and fungal pathogens. In contrast to CDR1, AED1, a predicted aspartyl protease, suppresses SAR^59^. In our study, 1 ASP (Ma03_g03840) was up-regulated in both banana genotypes challenged with *Foc* TR4. However, the roles of these two ASPs genes in banana-*Foc* TR4 interactions needs to be further validated.

GDSL lipase is a hydrolyser with a conserved GDSL motif at N′ terminus of the protein, and it is involved in various pathways including pathogen response. Oh *et al*.^60^ reported that a GDSL LIPASE1 (GLIP1) mutant enhanced susceptibility to the necrotrophic fungus *Alternaria brassicicola*, and a recombinant GLIP1 protein inhibited spore germination. Furthermore, overexpression of GLIP1 in plants increases resistance to various bacterial and fungal pathogens, and GLIP1 elicited systemic resistance via ethylene signaling^61^. GLIP2 also plays a role in resistance to *Erwinia carotovora*, but it was dependent on negative regulation of auxin signaling^62^. In our study, 1 GLIP (Ma02_g12980) was activated by *Foc* TR4 attack.

Peroxidases play several biological roles in plants, particularly in cellular ROS detoxification^63^ and cell wall modifications^64^, which have been considered to be biochemical markers for early identification of *Foc* resistance bananas^16^. In the present study, 1 POD (Ma06_g10290) was up-regulated in Pahang during all infection periods, but only up-regulated in Brazilian at later stages. Furthermore, a novel peroxidase CanPOD gene of pepper, which has 72% identity with Ma06_g10290, is involved in defense responses to *Phytophtora capsici* infection^65^.

Polyphenol oxidase is a ubiquitous copper metalloprotein in many organisms, and it can catalyze the oxidation of phenolic compounds to form highly toxic quinones, thus inhibiting the growth of pathogenic fungus^66^. Li and Steffens^67^ reported that the PPO-overexpressing tomato plants enhanced resistance to *Pseudomonas syringae* pv. *tomato*. In the present study, 1 PPO (Ma08_g34740) was activated in both banana genotypes at later infection stages, suggesting this gene played a role in response to *Foc* TR4.

Cytochrome P450s are a diverse array of multifunctional heme-thiolate proteins in plants that play critical roles in defense against pathogens by synthesis of lignin and defense compounds such as isoflavonoids, hydroxamic acids and glucosinolates^68^. In the present study, one cytochrome P450 (Ma05_g26350) was up-regulated in both banana genotypes after *Foc* TR4 inoculation, implying that banana might generate lignin and defense compounds to combat *Foc* TR4.

Transcription factors participate in a variety of different signaling pathways with diverse functions and play an important regulatory role in plant defense responses. WRKY is a superfamily of transcription regulators in plants that can bind specifically to W-box in the promoter of the target genes for transcriptional regulation^69^. WRKY transcription factors have been shown to not only regulate the expression of defense-related genes, but also regulate salicylate- and jasmonate-regulated disease response pathways. Ectopic over-expression of WRKY33 in *Arabidopsis* enhanced resistance to necrotrophic fungal pathogens^70^. In contrast, WRKY70 increased plant susceptibility to necrotrophic fungal pathogens because it acts as a repressor of jasmonate-regulated genes^71^. A total of 153 WRKY transcription factors were identified in the DH Pahang genome^25,41^. It was found that a large number (56) of WRKY genes showed inducible expression in banana root tissue during *Foc* infection^40^. During unchallenged conditions, 79 and 93 *MusaWRKYs* expressed in resistant (Karthobiumthum, ABB) and susceptible (Nendran, AAB) banana cultivars, respectively; upon root lesion nematode (*Pratylenchus coffeae*) infection, 49 and 40 *MusaWRKYs* were observed to be differentially regulated in resistant and susceptible cultivars, respectively^41^. Among them, *MusaWRKYs52* was supposed to play a role in resistance, whereas *MusaWRKYs69* and *MusaWRKYs92* may be acting as a repressor in a defense response^41^. In our study, 7 WRKY genes, including WRKY4 (Ma10_g03630), WRKY22 (Ma10_g06870), WRKY25 (Ma06_g34370) and WRKY26 (Ma03_g09270, Ma06_g01150, Ma08_g01650 and Ma11_g18140), which were involved in plant-pathogen pathway were twofold higher in Pahang than in Brazilian during untreated conditions, suggested that this expression of these WRKY genes might be associated with constitutive defense mechanism. WRKY23 (Ma06_g23500) was up-regulated in both banana genotypes after *Foc* TR4 inoculation, indicating it participated in general response to *Foc* TR4 in banana.

## Conclusion

In the present study, we assessed for the first time the transcriptome response in the corms of resistant and susceptible bananas during *Foc* TR4 infection stages. Before infection, a total of 6,319 DEGs were identified, of which 3,867 DEGs, expressed at higher levels in Pahang than those in Brazilian, were significantly enriched in the resistance-related pathways, including “plant-pathogen interaction”, “plant hormone signal transduction”, “flavonoid biosynthesis” and “degradation of aromatic compounds” pathways, suggesting that Pahang exhibited constitutive defense responses before *Foc* TR4 infection. At 1 dpi, eight of the proteins coded by DEGs were up-regulated in Pahang, and most proteins were involved in defending against pathogens such as PR1, POD, PPO and CHIT, whereas no DEG was identified in Brazilian, implying that Pahang exhibited inducible defense responses prior to Brazilian at the initial *Foc* TR4 infection stage. Additionally, we revealed that most key enzymatic genes involved in the phenylalanine metabolism pathway were up-regulated in corms of Brazilian at 7 dpi and 14 dpi, indicating that Brazilian might synthesize lignin and phytotoxin in response to *Foc* TR4 infection at later stages. Taking into account the paleopolyploid nature of the genome, the present work identified a few candidate genes in Pahang response to pathogen infection, thus greatly improving our understanding the mechanisms of Fusarium wilt resistance in Pahang.

## Materials and Methods

### Plant inoculation

*Musa acuminata* (subsp. *malaccensis***)** ‘Pahang’ (AA, ITC0609, 10.18730/9K93B) obtained from the International *Musa* Germplasm Transit Centre and *Musa* AAA Cavendish ‘Brazilian’ (commercial variety, in China) were used in the study. Transgenic *Foc* TR4 strain tagged with AmCyan protein (AmCyan-*Foc* TR4) was previously generated^72^. In short, the plasmid pZD 101-AmCyan expressing AmCyan protein and hygromycin B genes was linearized and transferred into wild type *Foc* TR4 strain 15-1, isolated from Yunnan, using the protoplasts transformation system. The AmCyan-*Foc* TR4 has similar pathogenicity to wild type strain 15-1 (Supplementary Fig. S1). Plants with 5-6 leaves and healthy root systems were inoculated with AmCyan-*Foc* TR4. The roots were cut to 3–5 cm, then soaked in a spore suspension of 1 × 10^6^ conidia/mL for 30 min. The mocks were dipped into sterile distilled water (ddH_2_O). All inoculated plants were transplanted into plastic pots filled with sterile vermiculite, and maintained in a phytotron at 28 °C, 80% humidity and a 16/8 h light/dark photoperiod.

### Microscopy observation and disease severity survey

AmCyan-*Foc* TR4 was used to monitor the infection process under an Olympus Fv10i microscope with two emission-collecting windows operating simultaneously. Excitation/emission wavelengths were 405 nm/460 to 500 nm for AmCyan protein and 405 nm/570 to 670 nm for plant cell wall autofluorescence^73^. To clear the infection process, the banana roots and corms were hand-sectioned to form cross sections to observe daily until 14 days post inoculation (dpi). Disease severity survey was conducted according to our previous studies^28^. The lesion size in dissected corms was rated for each plant according to five grades ranging from 0 to 4. 0, no lesion in corm; 1, 1–10% lesion area in corm; 2, 11–30% lesion area in corm; 3, 31–50% lesion area in corm; 4, more than 50% lesion area in corm. Disease index (%) = [∑ (grade × number of plants in that grade)/(4 × total number of assessed plants)] × 100.

### Sampling strategy

The banana corm is a compact ‘wood-like’ organ which comprises a massive central cylinder composed of large vascular bundles^74,75^. The central cylinder is the major water transport system in the corm, and it is also an important battleground of banana and *Foc* TR4. The banana will only survive if the central cylinder remains uninfected. Therefore, based on the infection processes of *Foc* TR4, disease index and symptoms in corms, we harvested the samples, which was located in the centre of corm central cylinder (Fig. 1), at 0, 1, 7, and 14 dpi for transcriptome analysis to reveal the mechanisms of bananas dynamic response to *Foc* TR4. The corm was cut into two parts longitudinally, and the center tissues of the corm central cylinder was extracted. Six plants were pooled together for each sample and the experiments were conducted with three independent biological replicates. All samples were frozen immediately in liquid nitrogen and stored at −70 °C until RNA extraction.

### RNA extraction and sequencing

Total RNA was extracted with Easypure Plant RNA Kit (Transgen, Code#ER301-01) and genomic DNA contamination removed with RNase-free DNase I (Transgen, Code#GD201-01) following the manufacturer’s protocols. RNA degradation and contamination were monitored on 1% agarose gels. RNA integrity was assessed using the RNA Nano 6000 Assay Kit of the Bioanalyzer 2100 system (Agilent Technologies, CA, USA) and all samples’ RNA integrity numbers (RIN) were greater than 8.0.

The library construction and Illumina sequencing were performed at Novogene Bioinformatics Technology Co., Ltd., Beijing, China (www.novogene.cn). Briefly, a total amount of 3 µg RNA per sample was used to construct a cDNA library with a fragment about 150 bp in length. Then paired-end reads were sequenced using the Illumina Hiseq platform. Reads containing adapter, reads containing ploy-N and low-quality reads from raw reads were removed to obtain filtered reads with FastQC software, whose Q20, Q30 and GC content were calculated at the same time. All the downstream analyses were based on the filtered reads with high quality. The paired-end filtered reads were aligned to the banana reference genome v2^26^ downloaded from the Banana Genome Hub (http://banana-genome-hub.southgreen.fr)^76^ using TopHat (v2.0.12). The gene expression level was analyzed by HTSeq (v0.6.1), and the FPKM (expected number of Fragments Per Kilobase of transcript sequence per Millions base pairs sequenced)^77^ was greater than 1 used as a threshold to determine gene expression in this study. Principal component analysis (PCA) was performed by GeneSpring GX (v7.3) based on the gene expression profiles of all samples.

### Identification and annotation of DEGs

Differentially expressed genes (DEGs) between samples of Pahang (resistant) versus Brazilian (susceptible) before treatment (P_untr vs. B_untr) and *Foc* TR4 inoculated samples versus mock inoculated samples (P_dpi vs. P_dmo: P_1dpi vs. P_1dmo & P_7dpi vs. P_7dmo & P_14dpi vs. P_14dmo; B_dpi vs. B_dmo: B_1dpi vs. B_1dmo & B_7dpi vs. B_7dmo & B_14dpi vs. B_14dmo) were identified using the DESeq R package (v1.18.0)^78^. Genes with an adjusted P-value < 0.05 were assigned as differentially expressed. A DEG of fold change with an absolute value of log_2_^ratio^ > 0 in read counts between two libraries was considered up-regulated expression; in contrast, log_2_^ratio^ < 0 was considered to down-regulated expression. DEGs were further annotated by Gene Ontology (GO) functional enrichment using GOseq (Release2.12)^79^, and Kyoto Encyclopedia of Genes and Genomes (KEGG) functional enrichment using KOBAS (2.0)^80^. DEGs were considered significantly enriched with an adjusted P-value < 0.05.

### DEG paralog identification in *Musa* genome and Paralog Inclusive Expression (PIE) analysis

Paralogous sequences of DEGs were identified in the *Musa* genome by BLASTp analysis using NCBI nr-protein database. *Musa* genes having higher scores than genes of other species were considered as paralogs. In order to verify whether identified paralogs were expressed and had any potential interference on the DEG impact, FPKM values of all paralogs were summed and compared among RNA-seq libraries.

### Assessment of DEGs with RT-qPCR

To assess the RNA-seq data, 11 DEGs, identified in P_1dpi vs. P_1dmo, were confirmed using real-time quantitative PCR (RT-qPCR). Primers designed with Primer-BLAST in NCBI were listed in the Supplementary Table S1. cDNA was synthesized using PrimeScript™ RT Master Mix (TaKaRa, Code No. RR036A) according to the manufacturer’s protocols. RT-qPCR system with the UNICON™ qPCR SYBR Green Master Mix (Yeasen, Code#10110AS) were performed in a Bio-Rad MyiQ single-color real-time PCR detection system. The 20 μL reaction mix contained 2 μL cDNA (0.5 ng/μL) templates, 10 μL SYBR Green Mix (1×), 0.4 μL each primer (10 μM) and ddH_2_O (RNAase free) 7.2 μL. The amplification program consisted of one cycle of 95 °C for 60 s, followed by 40 cycles of 95 °C for 10 s and 60 °C for 30 s, with the annealing temperature raised 0.5 °C at each step ranging from 65 °C to 95 °C, and each step stayed for 8 s for the acquisition signal to obtain a melting curve. Three independent biological replicates and three technical replicates of each sample were performed. The relative expression of target genes were calculated with 2^−∆∆CT^ method^81^ using the TIP41 as a reference gene^82^. The correlation coefficient (r) between RT-qPCR and RNA-seq data was calculated. If r ≥ 0.85, it is defined that the data of the two are highly consistent; 0.75 < r < 0.85, defined as consistent; r ≤ 0.75, defined as inconsistent.

### Use of experimental animals, and human participants

The authors confirm that all experiments were performed in accordance with relevant guidelines and regulations.

## Supplementary information

Supplementary Information

Supplementary Dataset

## Data Availability

All reads from RNA-seq experiment were deposited in NCBI’s Sequence Read Archive (SRA) under the BioProject PRJNA485562 (https://www.ncbi.nlm.nih.gov/bioproject/?term=PRJNA485562).
