# Supplementary material for: Transcriptomic analysis of resistant and susceptible banana corms in response to infection by Fusarium oxysporum f. sp. cubense tropical race 4
Source: Sci Rep. 2019 Jun 3;9:8199. doi: 10.1038/s41598-019-44637-x (PMC6546912; doi:10.1038/s41598-019-44637-x)
Supplement: Supplementary file 1 — Supplementary Information [file 41598_2019_44637_MOESM1_ESM.pdf]

Transcriptomic analysis of resistant and susceptible banana corms in response to infection by *Fusarium oxysporum* f. sp. *cubense* tropical race 4

Lei Zhang[1, 2, 6], Alberto Cenci[4], Mathieu Rouard[4], Dong Zhang[5], Yunyue Wang[1,*], Weihua Tang[5,*] ＆ Si-Jun Zheng[2, 3,*]

[1] State Key Laboratory for Conservation and Utilization of Bio-Resources in Yunnan, Ministry of Education Key Laboratory of Agriculture Biodiversity for Plant Disease Management, Yunnan Agricultural University, Kunming, 650201, China. [2]Agricultural Environment and Resources Institute, Yunnan Academy of Agricultural Sciences, Kunming, 650205, China. [3]Bioversity International, 2238 Beijing Road, Kunming 650205, Yunnan, China. [4]Bioversity International, Parc Scientifique Agropolis II, 34397 Montpellier, Cedex 5 France. [5]National Key Laboratory of Plant Molecular Genetics, CAS Center for Excellence in Molecular Plant Sciences, Institute of Plant Physiology and Ecology, Shanghai Institutes for Biological Sciences, Chinese Academy of Sciences, Shanghai 200032, China. [6]Dehong Agricultural Technology Extension Center, Mangshi, 678400, China. *Correspondence and requests for materials should be addressed to S.-J.Z. (email: s.zheng@cgiar.org), W.-H.T. (whtang@sibs.ac.cn) ＆Y.-Y.W. (1371209436@qq.com)

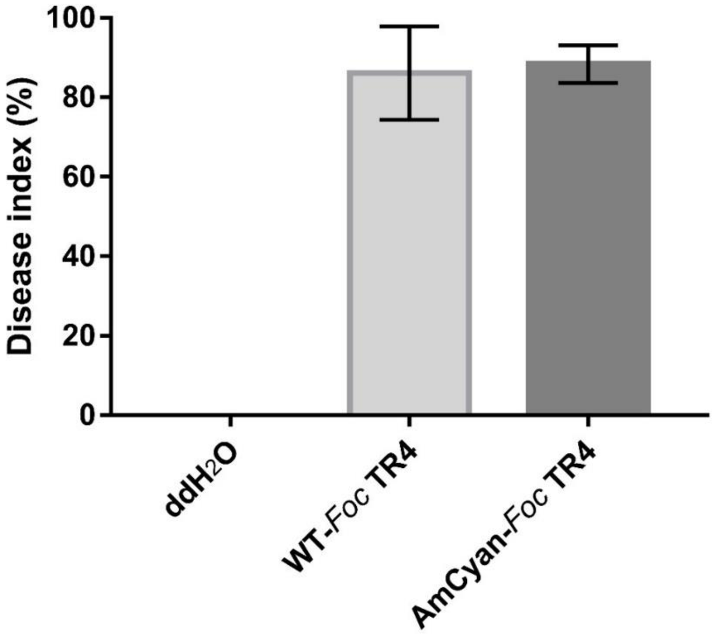

Supplementary Figure S1 Pathogenicity of AmCyan-*Foc* TR4 and WT-*Foc* TR4 derived from Yunnan. Disease index of Brazilian inoculated with ddH_2O, WT-*Foc* TR4 and AmCyan-*Foc* TR4 at 28 dpi. Error bars represent standard error of the mean with two independent experiments, and 9 plants were used in each treatment.

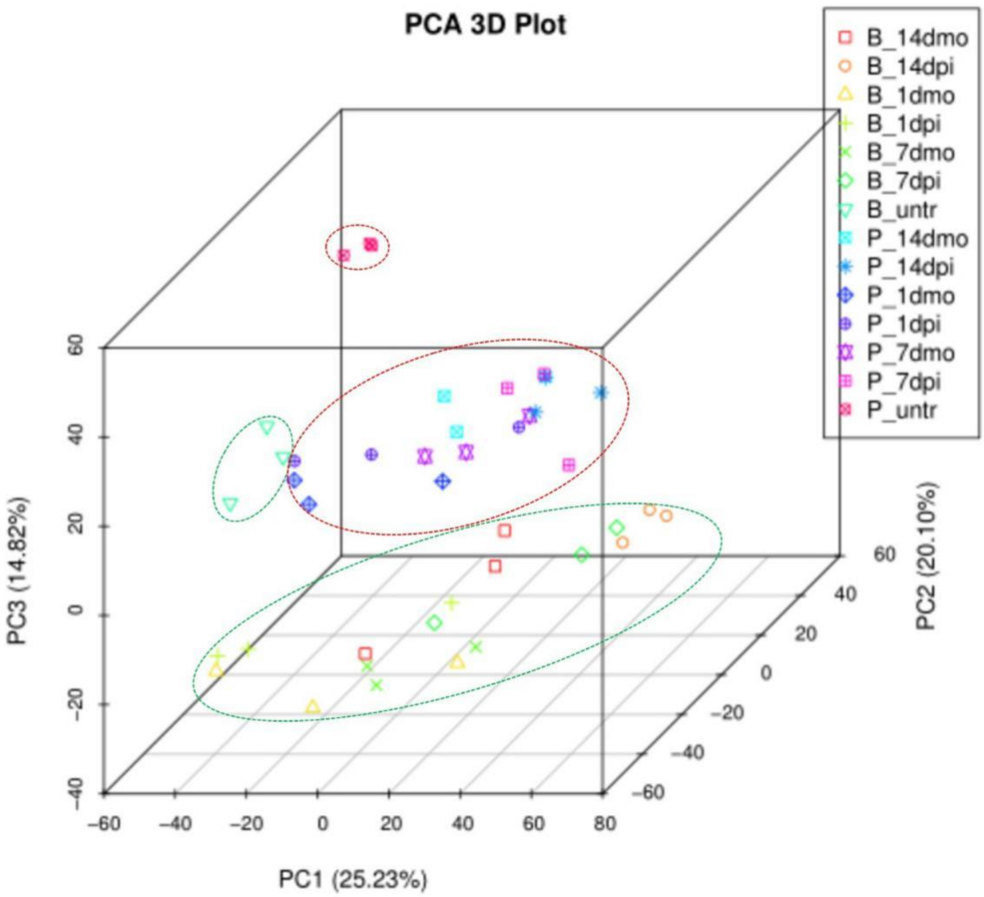

Supplementary Figure S2 Principal component analysis (PCA) of gene expression profiles of all samples.

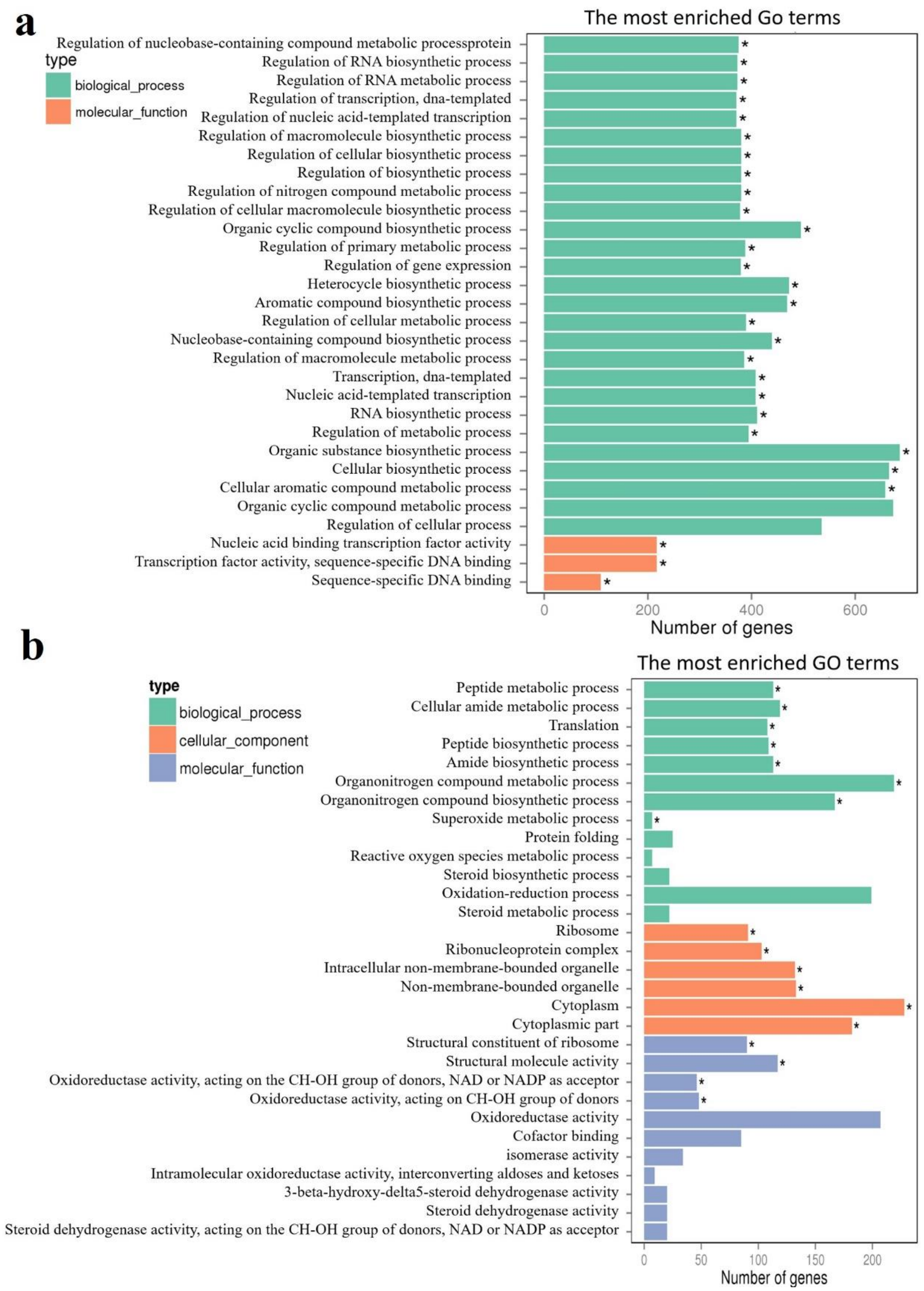

Supplementary Figure S3 Barplots of Pahang^{+} (a) and Brazilian^{+} (b) DEGs enriched GO terms (P_untr vs. B_untr). Asterisks indicate significantly enriched GO terms.

a

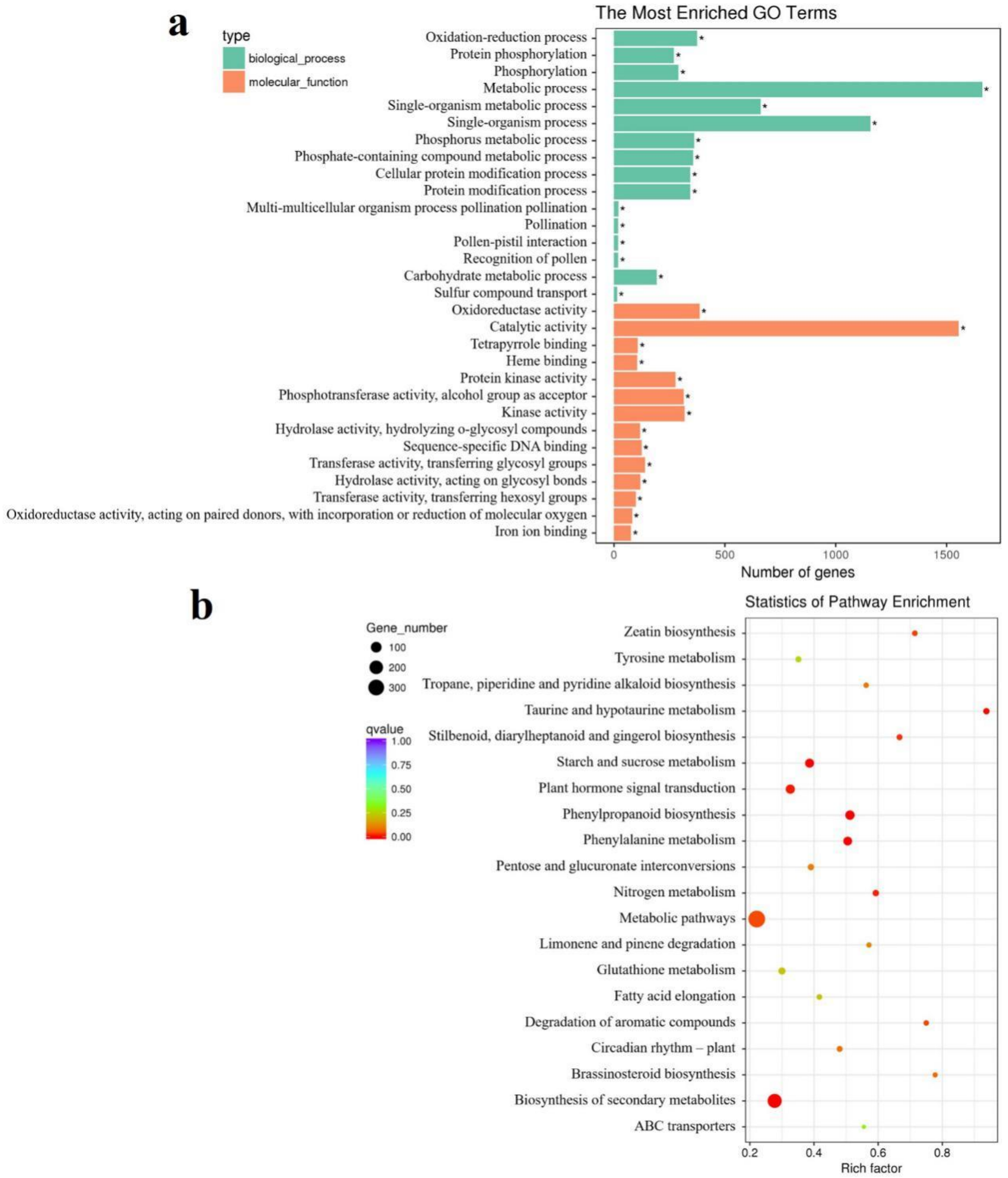

Supplementary Figure S4 Barplots of DEGs in Brazilian infected samples compared with mock samples (B_dpi vs. B_dmo) enriched GO terms (a) and the KEGG pathways (b). Asterisks indicate significantly enriched GO terms.

Supplementary Table S1 List of primers used in RT-qPCR

Gene id	Direction	Sequence/5'-3'	Product size/bp
Ma05_g26350	Forward	TCAACCTCATTGGCTCCCTG	151
	Reverse	GAGACGTCGTGGGTCTTGAG	
Ma08_g28490	Forward	GACTACGTGTGGGTGCAGTT	265
	Reverse	ACCCGCTGTTGAGATCGTAG	
Ma02_g12980	Forward	GCAATGACATCTACGCGCTG	187
	Reverse	ATTGCGACTCTGACCATCGG	
Ma08_g34160	Forward	GGTTACCTGACCACCAACCC	221
	Reverse	ACGTTACACTTCACACGCAC	
Ma06_g10290	Forward	CGTTGTGCTGCTACTTTCGG	205
	Reverse	CAAGAGCAACGACGCATCAC	
Ma03_g03840	Forward	CGCCTGGATAGTTTCGCCTT	197
	Reverse	TGGAATCATCGACCTGTGGC	
Ma08_g34740	Forward	GCTCGCTCGAGAACATACCA	132
	Reverse	CGTTTGAATGGTGGGCGAAG	
Ma03_g01020	Forward	ACGACGACCACTTCGAGAAC	232
	Reverse	ATGTTGCTGCCGACGATGAA	
Ma03_g08140	Forward	CCAGCGTTACCATTCGGCTA	195
	Reverse	GGGCAGTAGCTTGTAGGTGG	
Ma03_g11650	Forward	ACACCTGCTACGACTTCACG	266
	Reverse	CTTCGACCCTTAGCAGCCTC	
Ma08_g34150	Forward	TGTTGAGGTGTTGGACCACG	204
	Reverse	TCCGTCATCTTCTGCACCTC	
Ma06_g36690 (TIP41, reference gene)	Forward	TGGGTTGGATGCAAGAAGCA	214
	Reverse	ATTCGCTCGTCACAGTCCTC	

Supplementary Table S2 Summary of RNA-seq data quality

Sample name	Raw reads (R)	Filtered reads (C) (R/C)	Clean bases	Error rate	Q20	Q30
Brazilian	51793888	49626721 (95.82%)	7.44G	0.02%	95.44%	89.20%
Pahang	50763317	48900401 (96.33%)	7.34G	0.02%	95.60%	89.47%

Supplementary Table S3 Summary of read mapping

Sample	B_untr	B_1dmo	B_7dmo	B_14dmo	B_1dpi	B_7dpi	B_14dpi	P_untr	P_1dmo	P_7dmo	P_14dmo	P_1dpi	P_7dpi	P_14dpi
Total reads	51351143	50383524	53350958	48374121	45490725	51389749	47046826	51429291	48448129	47491282	50447841	52380300	46206952	45899013
Total mapped	34346866 (66.89%)	33020229 (65.54%)	35403336 (66.36%)	32040019 (66.23%)	30029321 (66.01%)	33871172 (65.91%)	30053325 (63.88%)	42508023 (82.65%)	41072401 (84.78%)	39951771 (84.12%)	41482628 (82.23%)	44115252 (84.22%)	38687929 (83.73%)	38081924 (82.97%)
Multiple mapped	314140 (0.61%)	339597 (0.67%)	507963 (0.95%)	374868 (0.77%)	277733 (0.61%)	384788 (0.75%)	450011 (0.96%)	430958 (0.84%)	472466 (0.98%)	422702 (0.89%)	340781 (0.68%)	416390 (0.79%)	391307 (0.85%)	361180 (0.79%)
Uniquely mapped	34032726 (66.27%)	32680633 (64.86%)	34895373 (65.41%)	31665151 (65.46%)	29751588 (65.40%)	33486384 (65.16%)	29603314 (62.92%)	42077065 (81.82%)	40599935 (83.80%)	39529069 (83.23%)	41141847 (81.55%)	43698863 (83.43%)	38296621 (82.88%)	37720744 (82.18%)
Read-1	17931060 (34.92%)	17984998 (35.70%)	18719654 (35.09%)	16883940 (34.90%)	16366416 (35.98%)	18074700 (35.17%)	16082552 (34.18%)	22032387 (42.84%)	21461240 (44.30%)	20945713 (44.10%)	22357491 (44.32%)	23310377 (44.50%)	20038551 (43.37%)	19952077 (43.47%)
Read-2	16101667 (31.36%)	14695634 (29.17%)	16175719 (30.32%)	14781212 (30.56%)	13385172 (29.42%)	15411684 (29.99%)	13520761 (28.74%)	20044678 (38.98%)	19138696 (39.50%)	18583356 (39.13%)	18784356 (37.24%)	20388486 (38.92%)	18258071 (39.51%)	17768667 (38.71%)
Reads map to '+'	16973088 (33.05%)	16280867 (32.31%)	17339636 (32.50%)	15731968 (32.52%)	14822045 (32.58%)	16676662 (32.45%)	14756511 (31.37%)	20989079 (40.81%)	20246666 (41.79%)	19707628 (41.50%)	20499071 (40.63%)	21794135 (41.61%)	19104154 (41.34%)	18803408 (40.97%)
Reads map to '-'	17059638 (33.22%)	16399765 (32.55%)	17555736 (32.91%)	15933184 (32.94%)	14929542 (32.82%)	16809722 (32.71%)	14846803 (31.56%)	21087986 (41.00%)	20353270 (42.01%)	19821441 (41.74%)	20642776 (40.92%)	21904728 (41.82%)	19192468 (41.54%)	18917336 (41.22%)
Non-splice reads	21100471 (41.09%)	19348963 (38.40%)	21365459 (40.05%)	19038555 (39.36%)	17232991 (37.88%)	20453255 (39.80%)	18505099 (39.33%)	28457318 (55.33%)	25859916 (53.38%)	25836129 (54.40%)	26377386 (52.29%)	27472164 (52.45%)	24922564 (53.94%)	24318731 (52.98%)
Splice reads	12932255 (25.18%)	13331670 (26.46%)	13529914 (25.36%)	12626596 (26.10%)	12518596 (27.52%)	13033129 (25.36%)	11098215 (23.59%)	13619747 (26.48%)	14740020 (30.42%)	13692940 (28.83%)	14764461 (29.27%)	16226699 (30.98%)	13374057 (28.94%)	13402013 (29.20%)

Supplementary Table S4 Number of genes in different expression levels

FPKM Interval	B_untr	B_1dmo	B_7dmo	B_14dmo	B_1dpi	B_7dpi	B_14dpi	P_untr	P_1dmo	P_7dmo	P_14dmo	P_1dpi	P_7dpi	P_14dpi
0-1	11563 (31.48%)	13152 (35.81%)	13127 (35.74%)	13226 (36.01%)	12882 (35.07%)	12761 (34.74%)	12533 (34.12%)	11330 (30.85%)	12221 (33.28%)	12573 (34.23%)	12527 (34.11%)	12179 (33.16%)	12399 (33.76%)	12583 (34.26%)
1-3	4675 (12.73%)	4556 (12.41%)	4538 (12.36%)	4693 (12.78%)	4586 (12.49%)	4756 (12.95%)	4880 (13.29%)	4705 (12.81%)	4491 (12.23%)	4415 (12.02%)	4490 (12.23%)	4496 (12.24%)	4522 (12.31%)	4449 (12.11%)
3-15	11008 (29.97%)	10375 (28.25%)	10481 (28.54%)	10270 (27.96%)	10488 (28.56%)	10281 (27.99%)	10324 (28.11%)	11262 (30.66%)	10925 (29.75%)	10758 (29.29%)	10733 (29.22%)	10937 (29.78%)	10672 (29.06%)	10671 (29.05%)
15-60	7091 (19.31%)	6614 (18.01%)	6633 (18.06%)	6566 (17.88%)	6693 (18.22%)	6688 (18.21%)	6784 (18.47%)	7043 (19.18%)	6991 (19.04%)	6901 (18.79%)	6856 (18.67%)	6951 (18.93%)	6951 (18.93%)	6905 (18.80%)
>60	2390 (6.51%)	2030 (5.53%)	1947 (5.30%)	1972 (5.37%)	2079 (5.66%)	2241 (6.10%)	2206 (6.01%)	2387 (6.50%)	2099 (5.71%)	2080 (5.66%)	2121 (5.77%)	2164 (5.89%)	2183 (5.94%)	2119 (5.77%)
>1	25164 (68.52%)	23575 (64.19%)	23600 (64.26%)	23501 (63.99%)	23845 (64.93%)	23966 (65.26%)	24194 (65.88%)	25397 (69.15)	24506 (66.72%)	24154 (65.77%)	24200 (65.89%)	24548 (66.84%)	24328 (66.24%)	24144 (65.74%)

Supplementary Table S5 Annotation and expression comparison of some DEGs identified in Pahang compared with Brazilian during unchallenged conditions (P_untr vs. B_untr)

Locus_ID	Chromosome	Start	End	Function	KEGG	Readcount_P_untr	Readcount_B_untr	Log_2FoldChange (P_untr vs. B_untr)	Pval	Padj	Significant
Ma01_g12160.1	chr01	8836059	8837402	bHLH14 transcription factor	Plant pathogen interaction	511.641088	97.11169051	2.3974	1.13E-17	2.93E-15	High
Ma05_g07880.1	chr05	5822104	5822781	Calcium-binding protein CML17	Plant pathogen interaction	1969.982055	3418.687646	-0.79526	0.0014383	0.011461	Low
Ma08_g00140.1	chr08	125536	126213	Calcium-binding protein CML18	Plant pathogen interaction	740.3209349	298.7596091	1.3092	9.36E-07	2.39E-05	High
Ma10_g19740.1	chr10	30187358	30187843	Calcium-binding protein CML18	Plant pathogen interaction	76.45007374	34.12618842	1.1636	0.0034989	0.023199	High
Ma04_g19290.1	chr04	21892488	21892931	Calcium-binding protein CML19	Plant pathogen interaction	721.644534	325.6387197	1.148	0.0068803	0.039842	High
Ma05_g14170.1	chr05	10303061	10303501	Calcium-binding protein CML19	Plant pathogen interaction	7.752710168	0.421052811	4.2026	0.0072741	0.041656	High
Ma09_g26940.1	chr09	38071431	38072039	Calcium-binding protein CML19	Plant pathogen interaction	205.1852533	62.30606339	1.7195	2.35E-05	0.00038339	High
Ma09_g04810.1	chr09	3103751	3104341	Calcium-binding protein CML27	Plant pathogen interaction	79.30391246	3.187505507	4.6369	2.39E-12	2.31E-10	High
Ma01_g21640.1	chr01	21549922	21550569	Calcium-binding protein CML31	Plant pathogen interaction	344.1912678	125.4817709	1.4557	0.00011165	0.0014043	High
Ma09_g24900.1	chr09	36515883	36516329	Calcium-binding protein CML7	Plant pathogen interaction	2051.738832	3409.127417	-0.73256	0.0026287	0.018606	Low
Ma05_g02560.1	chr05	1680854	1681645	Calcium-dependent protein kinase 1	Plant pathogen interaction	1.566906134	15.89351881	-3.3424	0.0032606	0.021969	Low

Ma05_g29940.1	chr05	40476055	40479321	Calcium-dependent protein kinase 17-like	Plant pathogen interaction	197.4033794	98.4794777	1.0033	0.0012605	0.010297	High
Ma09_g26340.1	chr09	37644970	37653221	Calcium-dependent protein kinase 1-like	Plant pathogen interaction	1155.873389	609.1166558	0.92419	0.00019578	0.0022592	High
Ma02_g12910.1	chr02	21507841	21514308	Calcium-dependent protein kinase 26-like	Plant pathogen interaction	2119.081815	783.2809214	1.4358	1.55E-06	3.70E-05	High
Ma02_g05200.1	chr02	16515000	16517195	Calcium-dependent protein kinase 26-like	Plant pathogen interaction	254.0722964	147.9452527	0.78018	0.006662	0.038882	High
Ma05_g19800.1	chr05	29209253	29270458	Calcium-dependent protein kinase 28-like	Plant pathogen interaction	64.18567552	183.4910741	-1.5154	1.45E-06	3.50E-05	Low
Ma01_g04260.1	chr01	2785438	2795061	Calcium-dependent protein kinase 28-like	Plant pathogen interaction	843.3434952	282.1732224	1.5795	3.54E-05	0.00054063	High
Ma04_g22080.1	chr04	24470794	24473357	Calcium-dependent protein kinase 29-like	Plant pathogen interaction	652.5866558	286.5801537	1.1872	5.30E-06	0.00010643	High
Ma04_g22120.1	chr04	24497832	24499053	Calcium-dependent protein kinase 29-like	Plant pathogen interaction	201.670315	57.58934069	1.8081	1.81E-08	7.24E-07	High
Ma04_g24830.1	chr04	26730849	26737045	Calcium-dependent protein kinase 3-like	Plant pathogen interaction	91.41659105	197.6669215	-1.1125	0.0001532	0.0018282	Low
Ma06_g33600.1	chr06	34169077	34176774	Calcium-dependent protein kinase 3-like	Plant pathogen interaction	1820.983679	847.3834794	1.1036	3.68E-06	7.80E-05	High
Ma09_g05250.1	chr09	3383837	3389450	Calcium-dependent protein kinase 3-like	Plant pathogen interaction	3675.739784	1675.567808	1.1334	1.01E-06	2.53E-05	High
Ma09_g08340.1	chr09	5524151	5529282	Calcium-dependent protein kinase SK5-like	Plant pathogen interaction	2796.925526	893.2853608	1.6466	1.03E-07	3.40E-06	High
Ma10_g16000.1	chr10	27879162	27883534	Calcium-dependent protein kinase SK5-like	Plant pathogen interaction	650.3941378	1093.249294	-0.74924	0.00344	0.022903	Low

Ma01_g07590.1	chr01	5503619	5505792	Calmodulin-2/4-like	Plant pathogen interaction	132.0718355	73.01113658	0.85513	0.0041496	0.026593	High
Ma02_g03180.1	chr02	14515095	14518911	Calmodulin-2-like	Plant pathogen interaction	814.7112141	467.0455079	0.80273	0.0013747	0.011076	High
Ma03_g26590.1	chr03	30254035	30254838	Calmodulin-like	Plant pathogen interaction	69.5065605	14.38435966	2.2726	5.99E-07	1.64E-05	High
Ma05_g08710.1	chr05	6431894	6432902	Calmodulin-like	Plant pathogen interaction	750.4639213	186.0087805	2.0124	2.57E-06	5.76E-05	High
Ma01_g21300.1	chr01	20951748	20953102	Calmodulin-like protein 11	Plant pathogen interaction	579.6714644	53.4677908	3.4385	3.83E-30	4.51E-27	High
Ma07_g01400.1	chr07	1091324	1092381	Calmodulin-like protein 11	Plant pathogen interaction	68.26567039	20.0377031	1.7684	5.08E-05	0.00072974	High
Ma09_g08840.1	chr09	5877064	5877762	Calmodulin-like protein 7	Plant pathogen interaction	379.6982433	204.547082	0.89242	0.0034043	0.022722	High
Ma11_g18400.1	chr11	23466778	23474671	Chitin elicitor receptor kinase 1-like	Plant pathogen interaction	1761.61971	948.6695491	0.89292	0.00019635	0.002264	High
Ma06_g09510.1	chr06	6659998	6663414	Cyclic nucleotide-gated ion channel 2-like	Plant pathogen interaction	97.54707782	26.52290294	1.8789	4.35E-06	8.99E-05	High
Ma05_g28680.1	chr05	39610643	39613347	Cyclic nucleotide-gated ion channel 4-like	Plant pathogen interaction	727.8682014	308.4198736	1.2388	7.89E-07	2.09E-05	High
Ma01_g04010.1	chr01	2616623	2619348	Cyclic nucleotide-gated ion channel 4-like	Plant pathogen interaction	343.768338	203.8415286	0.75399	0.004668	0.029233	High
Ma08_g31160.1	chr08	42161925	42165263	Disease resistance protein RPM1	Plant pathogen interaction	96.04287173	21.80105563	2.1393	0.0044741	0.028294	High
Ma04_g35240.1	chr04	34114225	34117080	Disease resistance protein RPS5	Plant pathogen interaction	1281.307017	340.2942396	1.9128	6.99E-11	4.89E-09	High

Ma07_g21730.1	chr07	29796267	29800161	Disease resistance RPP8-like protein 2~ RPP8L2	Plant pathogen interaction	29.72510499	2.459069064	3.5955	1.67E-06	3.95E-05	High
Ma07_g21750.1	chr07	29827068	29829247	Disease resistance RPP8-like protein 3~ RPP8L2	Plant pathogen interaction	24.08965141	7.425272831	1.6979	0.0075641	0.042891	High
Ma06_g08310.1	chr06	5894297	5895742	Elongation factor Tu, chloroplastic-like	Plant pathogen interaction	177.5475873	339.6590863	-0.93588	0.0011888	0.009817	Low
Ma07_g19020.1	chr07	27018816	27023913	Endoplasmin homolog	Plant pathogen interaction	908.0063982	2452.371789	-1.4334	0.00012354	0.0015279	Low
Ma10_g06370.1	chr10	18933346	18936486	Heat shock protein 81-1-like	Plant pathogen interaction	10327.25792	18231.36887	-0.81997	0.00068642	0.0063263	Low
Ma03_g29390.1	chr03	32310831	32318221	Heat shock protein 83-like	Plant pathogen interaction	959.4266262	1587.18107	-0.72622	0.0044044	0.027967	Low
Ma09_g23200.1	chr09	35116063	35121929	Heat shock protein 83-like	Plant pathogen interaction	354.0610311	794.7744001	-1.1665	3.13E-05	0.00048641	Low
Ma08_g02160.1	chr08	1754295	1757406	Hypothetical protein	Plant pathogen interaction	29.31726846	119.0369131	-2.0216	2.30E-08	8.99E-07	Low
Ma10_g02730.1	chr10	9341603	9349731	Mitogen-activated protein kinase 1-like	Plant pathogen interaction	437.553908	162.4867423	1.4291	0.00054198	0.0052048	High
Ma10_g27400.1	chr10	34714764	34718022	Mitogen-activated protein kinase 2-like	Plant pathogen interaction	117.0850009	53.29964319	1.1354	0.00069767	0.0064036	High
Ma03_g04840.1	chr03	3223761	3224807	Mitogen-activated protein kinase 5-like	Plant pathogen interaction	512.9048077	322.2046706	0.67071	0.005634	0.033994	High
Ma04_g05920.1	chr04	4429644	4430737	Mitogen-activated protein kinase 5-like	Plant pathogen interaction	443.9409385	182.7250081	1.2807	6.75E-05	0.00092913	High

Ma09_g13150.1	chr09	8897283	8898341	Mitogen-activated protein kinase 5-like	Plant pathogen interaction	268.7391863	110.4482291	1.2828	7.93E-06	0.00015035	High
Ma09_g23300.1	chr09	35189203	35190192	Mitogen-activated protein kinase 5-like	Plant pathogen interaction	826.3605081	243.4605576	1.7631	5.69E-11	4.06E-09	High
Ma09_g21380.1	chr09	32368484	32369737	MYC transcription factor	Plant pathogen interaction	20.73694995	114.6657466	-2.4672	0.00027922	0.0030251	Low
Ma04_g16760.1	chr04	16587449	16589190	MYC transcription factor	Plant pathogen interaction	67.65695123	20.8023117	1.7015	3.50E-05	0.00053487	High
Ma09_g05320.1	chr09	3436829	3441924	Respiratory burst oxidase homolog protein A-like	Plant pathogen interaction	990.443748	447.1447913	1.1473	0.0047451	0.029606	High
Ma06_g33550.1	chr06	34139349	34143943	Respiratory burst oxidase homolog protein A-like	Plant pathogen interaction	1576.267225	820.6871395	0.94161	0.00015222	0.0018179	High
Ma06_g13290.1	chr06	9101805	9106002	Respiratory burst oxidase homolog protein B	Plant pathogen interaction	20361.58097	6556.151291	1.6349	3.79E-12	3.46E-10	High
Ma10_g17360.1	chr10	28799994	28803354	Respiratory burst oxidase homolog protein C	Plant pathogen interaction	38.91533956	9.682954575	2.0068	0.00026033	0.0028585	High
Ma10_g17370.1	chr10	28803435	28805699	Respiratory burst oxidase homolog protein C	Plant pathogen interaction	190.7911062	68.48097527	1.4782	2.71E-05	0.00043046	High
Ma01_g11500.1	chr01	8324332	8327803	RPM1-interacting protein 4-like	Plant pathogen interaction	293.4751166	147.7627989	0.98996	0.001399	0.011234	High
Ma03_g12680.1	chr03	9766669	9767713	RPM1-interacting protein 4-like	Plant pathogen interaction	215.4842527	90.73903538	1.2478	3.25E-05	0.00050147	High
Ma06_g01310.1	chr06	1052091	1055343	RPM1-interacting protein 4-like	Plant pathogen interaction	155.7161185	71.53985006	1.1221	0.00057992	0.0055118	High
Ma11_g08140.1	chr11	6495590	6498967	RPM1-interacting protein 4-like	Plant pathogen interaction	640.5810767	236.309841	1.4387	1.03E-06	2.58E-05	High

Ma06_g22230.1	chr06	17458275	17464921	Serine/threonine-protein kinase PBS1-like	Plant pathogen interaction	1839.530276	1092.200451	0.7521	0.0014628	0.011607	High
Ma09_g01180.1	chr09	869660	870118	Serine/threonine-protein kinase PBS1-like	Plant pathogen interaction	239.7452498	89.89298584	1.4152	1.54E-05	0.00026523	High
Ma02_g23650.1	chr02	28577419	28578748	TIFY 10A~ TIFY4B	Plant pathogen interaction	2807.135482	898.3500858	1.6437	0.00017853	0.0020853	High
Ma07_g21360.1	chr07	29492840	29493963	TIFY 10A-like	Plant pathogen interaction	4748.133868	2783.296548	0.77057	0.00108	0.009093	High
Ma01_g15260.1	chr01	11056753	11058649	TIFY 10B~ TIFY7	Plant pathogen interaction	8084.405176	3049.033906	1.4068	2.59E-09	1.28E-07	High
Ma03_g09980.1	chr03	7440453	7441478	TIFY 10B-like	Plant pathogen interaction	6665.154079	3773.555057	0.82071	0.00047172	0.0046629	High
Ma05_g24500.1	chr05	36733367	36734468	TIFY 10B-like	Plant pathogen interaction	1442.982191	535.495154	1.4301	0.0025562	0.018191	High
Ma05_g24410.1	chr05	36664434	36666434	TIFY 3A-like	Plant pathogen interaction	1947.748092	1132.206609	0.78267	0.0056531	0.03408	High
Ma08_g25400.1	chr08	38253457	38258395	TIFY 3B-like	Plant pathogen interaction	1534.807727	781.4947509	0.97375	0.00011032	0.0013927	High
Ma09_g15040.1	chr09	10349843	10352808	TIFY 6B	Plant pathogen interaction	5598.68137	3597.959625	0.63791	0.0039506	0.025562	High
Ma07_g12340.1	chr07	9233446	9235955	TIFY 6B-like	Plant pathogen interaction	2954.536811	1736.038579	0.76713	0.0018404	0.013957	High
Ma09_g01070.1	chr09	801083	802396	TIFY 9~ TIFY6A	Plant pathogen interaction	4011.966351	455.0537782	3.1402	5.55E-13	6.20E-11	High
Ma10_g18410.1	chr10	29339063	29340577	TIFY 9~ TIFY6B	Plant pathogen interaction	207.4443839	15.75313021	3.719	2.96E-09	1.45E-07	High

Ma06_g22180.1	chr06	17425590	17426986	TIFY5A	Plant pathogen interaction	2483.576081	263.1687845	3.2384	6.74E-15	1.11E-12	High
Ma10_g06870.1	chr10	20403729	20404855	WRKY transcription factor 22-like	Plant pathogen interaction	98.5648818	2.876285442	5.0988	0.0024429	0.017556	High
Ma06_g34370.1	chr06	34630643	34632971	WRKY transcription factor 25	Plant pathogen interaction	9928.391412	3956.639242	1.3273	7.41E-09	3.30E-07	High
Ma03_g09720.1	chr03	7230957	7232357	WRKY transcription factor 26	Plant pathogen interaction	56.11448419	11.60730165	2.2733	0.0025009	0.017864	High
Ma06_g01150.1	chr06	930230	932422	WRKY transcription factor 26	Plant pathogen interaction	5434.396725	995.4883459	2.4486	3.15E-09	1.54E-07	High
Ma08_g01650.1	chr08	1399007	1401135	WRKY transcription factor 26	Plant pathogen interaction	557.3467619	280.9068597	0.98848	0.0042424	0.027095	High
Ma11_g18140.1	chr11	23279101	23281141	WRKY transcription factor 26	Plant pathogen interaction	3172.936569	407.8388659	2.9597	2.04E-14	3.16E-12	High
Ma10_g03630.1	chr10	14237185	14242020	WRKY transcription factor 4	Plant pathogen interaction	1664.824986	613.4738723	1.4403	2.83E-06	6.23E-05	High
Novel01290	chr10	29961228	29964781	Caltractin	Plant pathogen interaction	100.2536664	26.52383504	1.9183	1.71E-06	4.02E-05	High

Supplementary Table S6 Gene expression comparison for enzymes involved in phenylalanine biosynthesis pathway

Enzyme	Genes number	Fold change (B_1dpi vs. B_1dmo)	Fold change (B_7dpi vs. B_7dmo)	Fold change (B_14dpi vs. B_14dmo)	Fold change (P_1dpi vs. P_1dmo)	Fold change (P_7dpi vs. P_7dmo)	Fold change (P_14dpi vs. P_14dmo)
PAL	8	1.38	10.74**	4.83**	1.71	3.50*	1.59
C4H_1	3	1.77	171.34**	22.35**	5.84**	8.07**	2.78*
C4H_2	4	1.47	4.38**	4.09**	1.75	2.10*	1.47
4CL	6	0.98	2.74*	1.86	1.24	1.83	1.18
CHS	18	1.18	3.83*	5.07**	1.00	1.57	1.37
CHI (CHIL5)	1	0.85	0.24^{++}	0.43^{+}	0.74	1.16	0.36^{+}
CHI_1	1	1.25	0.68	0.81	0.94	0.95	0.42^{+}
CCR	3	1.08	0.62	0.13^{++}	1.18	0.66	0.72
CAD_ Class I	2	0.89	1.34	0.85	0.96	0.87	0.87
CAD_ Class II	2	1.22	1.27	1.63	1.10	1.43	1.35
CAD_ Class III	3	1.42	7.98**	8.58**	1.65	2.38*	1.51
CAD_ Class IV	3	1.00	1.19	1.36	0.81	0.86	0.89
POD	109	1.28	5.91**	3.20*	1.32	2.16*	0.90
HCT	15	1.05	4.96**	3.65*	1.22	2.08*	1.11
C3'H	1	1.35	3.30*	3.39*	1.28	1.69	0.96
CCoAOMT1	7	1.11	1.83	1.47	1.23	1.28	1.06

Note: The fold change between respective dpi/dmo FPKM values for Brazilian/Pahang samples. ** Fold change higher than 4 and * fold change between 2 and 4, which indicate clearly up-regulated and moderately up-regulated, respectively; $^{++}$ fold change lower than 0.25 and $^{+}$ fold change between 0.25 and 0.5, which indicate clearly down-regulated and moderately down-regulated, respectively.

Supplementary Table S7 Genes name of enzymes involved in phenylalanine biosynthesis

Enzyme	Gene name
PAL	Ma05_g20900, Ma11_g14940, Ma11_g21180 , Ma08_g14940, Ma05_g03720, Ma02_g00760, Ma01_g04420 and Ma09_g15390.
C4H_1	Ma03_g05380, Ma03_g05400 and Ma09_g13580.
C4H_2	Ma07_g19640, Ma10_g13130, Ma06_g10250 and Ma09_g04770.
4CL	Ma03_g13320, Ma04_g05890, Ma07_g05170, Ma01_g18930, Ma04_g12600 and Ma09_g23330.
CHS	Ma10_g20560, Ma10_g20570, Ma10_g20580, Ma10_g20590, Ma10_g20600, Ma02_g16120, Ma02_g24860, Ma03_g01020, Ma03_g23650, Ma05_g32080, Ma06_g09770, Ma06_g09780, Ma06_g10570, Ma06_g12370, Ma06_g17870, Ma10_g12390, Ma10_g12450 and Ma11_g24610.
CHI (CHIL5)	Ma11_g21950.
CHI_1	Ma04_g17380.
CCR	Ma11_g07890, Ma05_g04220 and Ma06_g00250.
CAD_ Class I	Ma04_g12960 and Ma04_g06170.
CAD_ Class II	Ma07_g07170 and Ma09_g2788.
CAD_ Class III	Ma09_g31400, Ma00_g03560 and Ma05_g11250.
CAD_ Class IV	Ma04_g20820, Ma04_g33020 and Ma10_g17990.
POD	Ma00_g02230, Ma00_g03690, Ma01_g03820, Ma01_g03830, Ma01_g15470, Ma01_g16980, Ma01_g21440, Ma01_g21520, Ma02_g04360, Ma02_g04920, Ma02_g04930, Ma02_g05430, Ma02_g06740, Ma02_g17250, Ma02_g17260, Ma02_g17270, Ma02_g17280, Ma02_g22420, Ma02_g22430, Ma02_g22440, Ma02_g22450, Ma02_g23580, Ma03_g02760, Ma03_g03390, Ma03_g03420, Ma03_g04630, Ma03_g06220, Ma03_g10060, Ma03_g13910, Ma03_g18370, Ma03_g21640, Ma04_g02030, Ma04_g04170, Ma04_g05290, Ma04_g06420, Ma04_g13290, Ma04_g16270, Ma04_g16700, Ma04_g16720, Ma04_g19300, Ma04_g30470, Ma04_g34410, Ma05_g15650, Ma05_g15710, Ma05_g22740, Ma05_g27300, Ma05_g27310, Ma05_g31950, Ma06_g00650, Ma06_g00660, Ma06_g02220, Ma06_g07210, Ma06_g10290, Ma06_g12910, Ma06_g12920, Ma06_g16060, Ma06_g16100, Ma06_g16130, Ma06_g19750, Ma06_g23670, Ma06_g23680, Ma06_g24110, Ma06_g32140, Ma07_g02740, Ma07_g04750, Ma07_g05870, Ma07_g05880, Ma07_g09210, Ma07_g09220, Ma07_g11120, Ma07_g23680, Ma07_g23690, Ma07_g25110, Ma08_g10630, Ma08_g12130, Ma08_g12140, Ma08_g12150, Ma08_g26600, Ma08_g26830, Ma08_g32520, Ma09_g04700,

	Ma09_g11490, Ma09_g11510, Ma09_g17630, Ma09_g24310, Ma09_g28800, Ma09_g29300, Ma09_g30120, Ma09_g30760, Ma09_g31450, Ma10_g01510, Ma10_g03120, Ma10_g05180, Ma10_g05350, Ma10_g07300, Ma10_g11670, Ma10_g14320, Ma10_g15940, Ma10_g25840, Ma10_g27800, Ma10_g27810, Ma10_g27820, Ma11_g02610, Ma11_g02620, Ma11_g02630, Ma11_g04630, Ma11_g19280, Ma11_g22010 and Ma11_g23900.
HCT	Ma04_g02000, Ma05_g04310, Ma10_g23880, Ma07_g16250, Ma07_g16240, Ma06_g03660, Ma04_g21340, Ma04_g00240, Ma02_g12390, Ma08_g03070, Ma08_g03090, Ma08_g03100, Ma08_g03110, Ma08_g03120 and Ma08_g20200.
C3'H	Ma08_g24760.
CCoAOMT1	Ma10_g03210, Ma10_g03220, Ma03_g06130, Ma06_g38330, Ma08_g19800, Ma09_g25700 and Ma08_g24230.

Supplementary Table S8 Annotation of DEGs identified in Pahang infected samples compared with mock samples (P_dpi vs. P_dmo)

Identification	DEGs_id	Annotation	Species
P_1dpi vs. P_1dmo	Ma02_g12980	GDSL esterase/lipase	*Arabidopsis thaliana*
	Ma03_g01020	Phenylpropanoylacetyl-CoA synthase	*Curcuma longa*
	Ma03_g03840	Aspartic proteinase nepenthesin-2	*Nepenthes gracilis*
	Ma03_g08140	Pathogenesis-related protein 1	*Asparagus officinalis*
	Ma03_g11650	Protein ASPARTIC PROTEASE IN GUARD CELL 2	*Arabidopsis thaliana*
	Ma05_g26350	Cytochrome P450	*Arabidopsis thaliana*
	Ma06_g10290	Peroxidase 4	*Vitis vinifera*
	Ma08_g28490	Acidic endochitinase	*Vitis vinifera*
	Ma08_g34150	Pathogenesis-related protein 1	*Asparagus officinalis*
	Ma08_g34160	Pathogenesis-related protein 1	*Asparagus officinalis*
	Ma08_g34740	Polyphenol oxidase, chloroplastic	*Malus domestica*
P_7dpi vs. P_7dmo	Ma02_g23450	3-ketoacyl-CoA synthase 6	*Arabidopsis thaliana*
	Ma04_g13440	Unknown	
P_14dpi vs. P_14dmo	Ma03_g01410	Sphinganine C(4)-monooxygenase 2	*Arabidopsis thaliana*
	Ma05_g13280	Putative glucuronosyltransferase	*Arabidopsis thaliana*
	Ma05_g17850	Chitinase 6	*Oryza sativa subsp. japonica*
	Ma06_g23500	Probable WRKY transcription factor 23	*Arabidopsis thaliana*
