# Supplementary material for: Transcriptomic analysis of resistant and susceptible banana corms in response to infection by Fusarium oxysporum f. sp. cubense tropical race 4
Source: Sci Rep. 2019 Jun 3;9:8199. doi: 10.1038/s41598-019-44637-x (PMC6546912; doi:10.1038/s41598-019-44637-x)
Supplement: Supplementary file 2 — Supplementary Dataset [file 41598_2019_44637_MOESM2_ESM.pdf]

Transcriptomic analysis of resistant and susceptible banana corms in response to infection by *Fusarium oxysporum* f. sp. *cubense* tropical race 4

Lei Zhang[1, 2, 6], Alberto Cenci[4], Mathieu Rouard[4], Dong Zhang[5], Yunyue Wang[1,*], Weihua Tang[5,*] ＆ Si-Jun Zheng[2, 3,*]

[1] State Key Laboratory for Conservation and Utilization of Bio-Resources in Yunnan, Ministry of Education Key Laboratory of Agriculture Biodiversity for Plant Disease Management, Yunnan Agricultural University, Kunming, 650201, China. [2]Agricultural Environment and Resources Institute, Yunnan Academy of Agricultural Sciences, Kunming, 650205, China. [3]Bioversity International, 2238 Beijing Road, Kunming 650205, Yunnan, China. [4]Bioversity International, Parc Scientifique Agropolis II, 34397 Montpellier, Cedex 5 France. [5]National Key Laboratory of Plant Molecular Genetics, CAS Center for Excellence in Molecular Plant Sciences, Institute of Plant Physiology and Ecology, Shanghai Institutes for Biological Sciences, Chinese Academy of Sciences, Shanghai 200032, China. [6]Dehong Agricultural Technology Extension Center, Mangshi, 678400, China. *Correspondence and requests for materials should be addressed to S.-J.Z. (email: s.zheng@cgiar.org), W.-H.T. (whtang@sibs.ac.cn) ＆Y.-Y.W. (1371209436@qq.com)

The most enriched pathway terms

Statistic method: hypergeometric test

FDR correction method: Benjamini and Hochberg

Term	Sample number	Background number	P-value	Corrected P-value	UniGenes	KO	Entrez ID	Ensembl ID	Gene name
Plant-pathogen interaction	85	130	6.3356598576e-07	7.53943523055e-05	Ma11_g08140	osa:4334782	4334782		
					Ma07_g01400	osa:4344344	4344344		
					Ma06_g09510	osa:4334172	4334172		
					Ma09_g13150	osa:4340367	4340367		
					Ma10_g17360	osa:4326027	4326027		
					Ma10_g16000	osa:4349899	4349899		
					Ma04_g05920	osa:4330957	4330957		
					Ma08_g01650	osa:4327518	4327518		
					Ma09_g21380	osa:4349484	4349484		
					Ma05_g28680	osa:4339240	4339240		
					Ma04_g22120	osa:4343373	4343373		
					Ma07_g12340	osa:4347000	4347000		
					Ma04_g22080	osa:4343373	4343373		
					Ma10_g27400	osa:4340126	4340126		
					Ma03_g04840	osa:4340367	4340367		
					Ma07_g21730	osa:4342596	4342596		
					Ma06_g22180	osa:4335698	4335698		
					Ma06_g01150	osa:4327518	4327518		
					Ma09_g23300	osa:4330957	4330957		
					Ma01_g04010	osa:4339240	4339240		
					Ma04_g24830	osa:4339751	4339751		
					Ma10_g06870	osa:4328953	4328953		
					Ma09_g05320	osa:4339397	4339397		
					Ma02_g03180	osa:4339172	4339172		
					Ma03_g09980	osa:4333065	4333065		
					Ma05_g19800	osa:4328155	4328155		
					Ma02_g23650	osa:4343899	4343899		
					Ma08_g31160	osa:4348062	4348062		
					Ma09_g15040	osa:4347000	4347000		
					Ma09_g24900	osa:4344525	4344525		
					Ma07_g21750	osa:4342596	4342596		
					Ma05_g24410	osa:4337240	4337240		
					Novel01290	osa:4343922	4343922		
					Ma01_g15260	osa:4343899	4343899		
					Ma06_g33550	osa:4339397	4339397		
					Ma09_g01180	osa:4329463	4329463		
					Ma09_g08840	osa:4336258	4336258		
					Ma05_g07880	osa:4325693	4325693		
					Ma08_g25400	osa:4337240	4337240		
					Ma09_g23200	osa:4345893	4345893		
					Ma06_g13290	osa:4326027	4326027		
					Ma10_g18410	osa:4335698	4335698		
					Ma09_g26340	osa:4342456	4342456		
					Ma03_g09720	osa:4327518	4327518		
					Ma01_g21300	osa:4344344	4344344		
					Ma09_g04810	osa:4332731	4332731		
					Ma05_g08710	osa:4344344	4344344		
					Ma06_g08310	osa:9270837	9270837		
					Ma10_g06370	osa:9267802	9267802		
					Ma04_g09630	osa:4337240	4337240		
					Ma10_g17370	osa:4326027	4326027		
					Ma08_g00140	osa:4325762	4325762		
					Ma10_g02730	osa:4333845	4333845		
					Ma05_g24500	osa:4333065	4333065		
					Ma01_g04260	osa:4328155	4328155		
					Ma04_g35240	osa:4325862	4325862		
					Ma03_g12680	osa:4334782	4334782		
					Ma05_g02560	osa:4352234	4352234		
					Ma06_g22230	osa:4329463	4329463		
					Ma09_g01070	osa:4335698	4335698		
					Ma09_g08340	osa:4349899	4349899		
					Ma10_g03630	osa:4327518	4327518		
					Ma05_g14170	osa:4325408	4325408		
					Ma03_g29390	osa:4345893	4345893		
					Ma03_g26590	osa:4344344	4344344		
					Ma09_g05250	osa:4339751	4339751		
					Ma04_g19290	osa:4325408	4325408		
					Ma10_g19740	osa:4325762	4325762		
					Ma02_g05200	osa:4330351	4330351		

					Ma11_g18140	osa:4327518	4327518		
					Ma11_g18400	osa:4346173	4346173		
					Ma01_g07590	osa:4326903	4326903		
					Ma08_g02160	osa:4338955	4338955		
					Ma05_g29940	osa:4327560	4327560		
					Ma04_g16760	osa:4349484	4349484		
					Ma06_g33600	osa:4339751	4339751		
					Ma01_g11500	osa:4334782	4334782		
					Ma06_g01310	osa:4334782	4334782		
					Ma07_g21360	osa:4333065	4333065		
					Ma02_g12910	osa:4330351	4330351		
					Ma09_g26940	osa:4334250	4334250		
					Ma01_g12160	osa:4349484	4349484		
					Ma07_g19020	osa:4342077	4342077		
					Ma06_g34370	osa:4327518	4327518		
					Ma01_g21640	osa:4325408	4325408		
Plant hormone signal transduction	92	184	0.000520854457399	0.0256333619695	Ma04_g20370	osa:4334864	4334864		
					Ma09_g21380	osa:4349484	4349484		
					Ma11_g22080	osa:4332548	4332548		
					Ma09_g15040	osa:4347000	4347000		
					Ma04_g02770	osa:4325895	4325895		
					Ma02_g18450	osa:4327315	4327315		
					Ma09_g16910	osa:4332697	4332697		
					Ma07_g09020	osa:4327315	4327315		
					Ma02_g14470	osa:4352722	4352722		
					Ma03_g06930	osa:4347257	4347257		
					Ma07_g25420	osa:4339797	4339797		
					Ma09_g01070	osa:4335698	4335698		
					Ma02_g08720	osa:4349800	4349800		
					Ma06_g35720	osa:4324749	4324749		
					Ma07_g09130	osa:9270643	9270643		
					Ma05_g24410	osa:4337240	4337240		
					Ma06_g17510	osa:4339158	4339158		
					Ma01_g11950	osa:4349090	4349090		
					Ma01_g04450	osa:9271447	9271447		
					Ma07_g13570	osa:4339365	4339365		
					Ma05_g11100	osa:4329252	4329252		
					Ma01_g14850	osa:4333017	4333017		
					Ma02_g23650	osa:4343899	4343899		
					Ma02_g00520	osa:4344349	4344349		
					Ma10_g18410	osa:4335698	4335698		
					Ma08_g25810	osa:4339756	4339756		
					Ma01_g15260	osa:4343899	4343899		
					Ma08_g22320	osa:4332697	4332697		
					Ma04_g09630	osa:4337240	4337240		
					Ma05_g24500	osa:4333065	4333065		
					Ma08_g22800	osa:4349742	4349742		
					Ma11_g22370	osa:4327465	4327465		
					Ma06_g30730	osa:4335937	4335937		
					Ma07_g10790	osa:4325895	4325895		
					Ma11_g16000	osa:4336520	4336520		
					Ma02_g24350	osa:4333860	4333860		
					Ma07_g21350	osa:4347154	4347154		
					Ma06_g14500	osa:4335877	4335877		
					Ma05_g31380	osa:4327465	4327465		
					Ma03_g31440	osa:4343785	4343785		
					Ma10_g21790	osa:4328916	4328916		
					Ma03_g31200	osa:4343719	4343719		
					Ma04_g16760	osa:4349484	4349484		
					Ma01_g12160	osa:4349484	4349484		
					Ma05_g10960	osa:4327590	4327590		
					Ma11_g20770	osa:4331518	4331518		
					Ma04_g39240	osa:4343785	4343785		
					Ma03_g09980	osa:4333065	4333065		
					Ma05_g05370	osa:4345817	4345817		
					Ma02_g13450	osa:4332697	4332697		
					Ma08_g33950	osa:4337780	4337780		
					Ma08_g25400	osa:4337240	4337240		
					Ma04_g12040	osa:4334864	4334864		
					Ma06_g32210	osa:4347257	4347257		
					Ma09_g04540	osa:4332548	4332548		
					Ma08_g17700	osa:4333860	4333860		
					Ma08_g05340	osa:4327315	4327315		
					Ma05_g29830	osa:4338764	4338764		
					Ma07_g25770	osa:4329672	4329672		
					Ma02_g13090	osa:4327590	4327590		
					Ma07_g27910	osa:4343785	4343785		
					Ma11_g10390	osa:4335877	4335877		
					Ma04_g31240	osa:4349090	4349090		
					Ma09_g14850	osa:4330855	4330855		
					Ma03_g10900	osa:4347439	4347439		
					Ma09_g27550	osa:4331061	4331061		
					Ma11_g24520	osa:4338933	4338933		
					Ma05_g07890	osa:4349742	4349742		
					Ma10_g19630	osa:4333860	4333860		
					Ma06_g34440	osa:4332548	4332548		

					Ma10_g18590	osa:4343785	4343785		
					Ma07_g12340	osa:4347000	4347000		
					Ma07_g21360	osa:4333065	4333065		
					Ma08_g12900	osa:4335058	4335058		
					Ma06_g22180	osa:4335698	4335698		
					Ma03_g27100	osa:4327315	4327315		
					Ma09_g26000	osa:4352721	4352721		
					Ma04_g15670	osa:4339756	4339756		
					Ma10_g26870	osa:4328647	4328647		
					Ma04_g08500	osa:4339756	4339756		
					Ma09_g27730	osa:4347257	4347257		
					Ma11_g23440	osa:4332697	4332697		
					Ma11_g06370	osa:4352722	4352722		
					Ma04_g10570	osa:4325895	4325895		
					Ma04_g27640	osa:4352722	4352722		
					Ma08_g02160	osa:4338955	4338955		
					Ma05_g15140	osa:4349090	4349090		
					Ma05_g22010	osa:4334864	4334864		
					Ma04_g38570	osa:4325503	4325503		
					Ma05_g24620	osa:4328277	4328277		
					Ma02_g02830	osa:4325078	4325078		
					Ma06_g37840	osa:4324691	4324691		
Biosynthesis of secondary metabolites	312	779	0.000646219209314	0.0256333619695	Ma08_g33280	osa:4325067	4325067		
					Ma10_g17260	osa:4343523	4343523		
					Ma03_g20800	osa:4340547	4340547		
					Ma11_g19280	osa:4344045	4344045		
					Ma09_g06710	osa:4339645	4339645		
					Ma07_g28490	osa:4352223	4352223		
					Ma06_g14390	osa:4337818	4337818		
					Ma10_g15940	osa:4324554	4324554		
					Ma10_g28640	osa:4340677	4340677		
					Ma06_g21190	osa:4337048	4337048		
					Ma11_g08300	osa:4336216	4336216		
					Ma07_g11340	osa:4348582	4348582		
					Ma09_g30120	osa:4337482	4337482		
					Ma02_g07020	osa:4347074	4347074		
					Ma08_g28660	osa:4333107	4333107		
					Ma06_g30180	osa:4350358	4350358		
					Ma10_g27460	osa:4327329	4327329		
					Ma07_g24940	osa:4344470	4344470		
					Ma04_g37810	osa:4333932	4333932		
					Ma09_g11280	osa:4348138	4348138		
					Ma09_g03450	osa:4332375	4332375		
					Ma06_g27120	osa:4329593	4329593		
					Ma06_g31980	osa:4350476	4350476		
					Ma08_g33830	osa:4328446	4328446		
					Ma09_g07380	osa:4332341	4332341		
					Ma10_g18160	osa:4332014	4332014		
					Ma09_g20310	osa:4331152	4331152		
					Ma06_g24480	osa:4332771	4332771		
					Ma11_g20920	osa:4348176	4348176		
					Ma06_g14400	osa:4337818	4337818		
					Ma10_g25720	osa:4345657	4345657		
					Ma04_g00620	osa:4335984	4335984		
					Ma05_g15910	osa:4333177	4333177		
					Ma11_g15760	osa:4343080	4343080		
					Ma06_g09580	osa:4346136	4346136		
					Ma08_g04770	osa:4334003	4334003		
					Ma05_g22300	osa:4349897	4349897		
					Ma08_g06060	osa:4337733	4337733		
					Ma06_g17200	osa:4344386	4344386		
					Ma03_g01840	osa:4343908	4343908		
					Ma03_g16060	osa:4332151	4332151		
					Ma06_g21530	osa:4325444	4325444		
					Ma05_g20350	osa:4334308	4334308		
					Ma11_g07140	osa:4342137	4342137		
					Ma01_g04420	osa:4336415	4336415		
					Ma07_g28480	osa:4352223	4352223		
					Ma10_g24940	osa:4347311	4347311		
					Ma03_g00220	osa:4331150	4331150		
					Ma10_g17210	osa:4352042	4352042		
					Ma02_g22450	osa:4324557	4324557		
					Ma04_g12630	osa:4341663	4341663		
					Ma04_g28070	osa:4352833	4352833		
					Ma09_g03470	osa:4332375	4332375		
					Ma08_g16510	osa:4331854	4331854		
					Ma06_g08980	osa:4351698	4351698		
					Ma08_g07480	osa:4325027	4325027		
					Ma09_g08110	osa:4350053	4350053		
					Ma04_g29400	osa:4333566	4333566		
					Ma03_g09880	osa:4332519	4332519		
					Ma03_g27870	osa:4347691	4347691		
					Ma10_g10490	osa:4329593	4329593		
					Ma05_g21330	osa:4327981	4327981		
					Ma04_g07860	osa:4327859	4327859		
					Ma08_g11970	osa:4336753	4336753		

					Ma10_g20560	osa:4350636	4350636		
					Ma07_g09220	osa:4341249	4341249		
					Ma08_g33290	osa:4325067	4325067		
					Ma09_g25700	osa:4340240	4340240		
					Ma04_g11650	osa:4324809	4324809		
					Ma06_g14310	osa:4351698	4351698		
					Ma08_g08880	osa:4346159	4346159		
					Ma05_g00310	osa:4341792	4341792		
					Ma01_g21520	osa:4344496	4344496		
					Ma03_g26140	osa:4338768	4338768		
					Ma08_g06070	osa:4352549	4352549		
					Ma09_g20570	osa:4337406	4337406		
					Ma07_g28090	osa:4333156	4333156		
					Ma07_g06700	osa:4349111	4349111		
					Ma06_g29250	osa:4332041	4332041		
					Ma01_g17990	osa:4343721	4343721		
					Ma06_g28330	osa:4339812	4339812		
					Ma05_g16190	osa:4331917	4331917		
					Ma04_g18620	osa:4334912	4334912		
					Ma03_g05400	osa:4329329	4329329		
					Ma11_g23980	osa:4344934	4344934		
					Ma10_g23920	osa:4345689	4345689		
					Ma10_g16100	osa:4337818	4337818		
					Ma04_g10090	osa:4326266	4326266		
					Ma08_g29910	osa:4350053	4350053		
					Ma07_g02100	osa:4337733	4337733		
					Ma03_g06130	osa:4340240	4340240		
					Ma01_g20320	osa:4327172	4327172		
					Ma03_g11800	osa:4346529	4346529		
					Ma05_g23060	osa:4349318	4349318		
					Ma09_g07020	osa:4352058	4352058		
					Ma02_g21000	osa:4332373	4332373		
					Ma08_g03130	osa:4333898	4333898		
					Ma02_g09950	osa:4350053	4350053		
					Ma06_g35790	osa:4324810	4324810		
					Ma04_g38030	osa:4333566	4333566		
					Ma07_g10560	osa:4327301	4327301		
					Ma06_g16590	osa:4324619	4324619		
					Ma03_g16070	osa:4339583	4339583		
					Ma07_g16390	osa:4350476	4350476		
					Ma07_g08400	osa:4341445	4341445		
					Ma04_g39130	osa:4334171	4334171		
					Ma04_g02250	osa:4337265	4337265		
					Ma05_g01700	osa:4346642	4346642		
					Ma05_g25700	osa:4327423	4327423		
					Ma06_g24910	osa:4339677	4339677		
					Ma04_g02590	osa:4332425	4332425		
					Ma04_g10150	osa:4345689	4345689		
					Ma06_g29240	osa:4332041	4332041		
					Ma09_g07280	osa:4329858	4329858		
					Ma04_g03820	osa:4335430	4335430		
					Ma08_g19500	osa:4335756	4335756		
					Ma08_g20630	osa:4342508	4342508		
					Ma09_g09840	osa:4324282	4324282		
					Ma07_g28080	osa:4333156	4333156		
					Ma02_g18360	osa:4327301	4327301		
					Ma05_g04020	osa:4336116	4336116		
					Ma04_g32180	osa:4332236	4332236		
					Ma08_g21280	osa:4329007	4329007		
					Ma06_g10250	osa:4338409	4338409		
					Ma03_g03600	osa:4328168	4328168		
					Ma09_g14520	osa:4347311	4347311		
					Ma06_g14410	osa:4337818	4337818		
					Ma06_g01670	osa:4338802	4338802		
					Novel01132	osa:4324282	4324282		
					Ma01_g03570	osa:4340042	4340042		
					Ma11_g15660	osa:4348804	4348804		
					Ma04_g32060	osa:4331540	4331540		
					Ma08_g05120	osa:4342988	4342988		
					Ma09_g23330	osa:4328485	4328485		
					Ma11_g16750	osa:4341770	4341770		
					Ma11_g19740	osa:4330012	4330012		
					Ma06_g09780	osa:4350636	4350636		
					Ma02_g24860	osa:4350636	4350636		
					Ma08_g30510	osa:4344267	4344267		
					Ma05_g27310	osa:4347520	4347520		
					Ma05_g27700	osa:4336216	4336216		
					Ma01_g17610	osa:4337406	4337406		
					Ma05_g30490	osa:4332519	4332519		
					Ma11_g17540	osa:4336216	4336216		
					Ma04_g35390	osa:4349454	4349454		
					Ma03_g00730	osa:9266710	9266710		
					Ma04_g31940	osa:4330090	4330090		
					Ma03_g12070	osa:4331418	4331418		
					Ma09_g12600	osa:4330747	4330747		
					Ma06_g12620	osa:4334188	4334188		
					Ma06_g01900	osa:4341792	4341792		

					Ma10_g14250	osa:4332425	4332425		
					Ma06_g07590	osa:4333918	4333918		
					Ma05_g20810	osa:4332477	4332477		
					Ma09_g24170	osa:4337437	4337437		
					Ma02_g14780	osa:4333566	4333566		
					Ma05_g05050	osa:4340042	4340042		
					Ma08_g29310	osa:4329889	4329889		
					Ma06_g11320	osa:4325687	4325687		
					Ma09_g21880	osa:4339535	4339535		
					Ma06_g23970	osa:4326635	4326635		
					Ma08_g04920	osa:4332153	4332153		
					Ma06_g38960	osa:4336353	4336353		
					Ma09_g02110	osa:4336216	4336216		
					Ma01_g02270	osa:4342142	4342142		
					Ma08_g24890	osa:4330711	4330711		
					Ma08_g27070	osa:4332134	4332134		
					Ma04_g31690	osa:4331611	4331611		
					Ma08_g28590	osa:4324398	4324398		
					Novel00760	osa:4324442	4324442		
					Ma11_g21050	osa:4330016	4330016		
					Ma05_g24340	osa:4341967	4341967		
					Ma03_g05860	osa:4329701	4329701		
					Ma05_g29980	osa:4349683	4349683		
					Ma07_g22000	osa:4345962	4345962		
					Ma07_g06220	osa:4345284	4345284		
					Ma05_g31950	osa:4351300	4351300		
					Ma06_g13460	osa:4352042	4352042		
					Novel01419	osa:4324282	4324282		
					Ma03_g28580	osa:4339885	4339885		
					Ma11_g04940	osa:4348304	4348304		
					Ma07_g11510	osa:4331893	4331893		
					Ma03_g05380	osa:4329324	4329324		
					Ma09_g19150	osa:4336750	4336750		
					Ma03_g06120	osa:4340725	4340725		
					Ma02_g13970	osa:4328168	4328168		
					Ma09_g23530	osa:4336928	4336928		
					Ma04_g07830	osa:4327859	4327859		
					Ma05_g18650	osa:4329532	4329532		
					Novel01203	osa:4334425	4334425		
					Ma01_g16200	osa:4332563	4332563		
					Ma10_g25840	osa:4332174	4332174		
					Ma10_g20600	osa:4350636	4350636		
					Ma03_g31530	osa:4352146	4352146		
					Ma04_g01300	osa:4352871	4352871		
					Ma08_g15070	osa:4348230	4348230		
					Ma08_g14810	osa:4349044	4349044		
					Ma05_g16480	osa:4332311	4332311		
					Ma06_g30380	osa:4329938	4329938		
					Ma06_g08130	osa:4352058	4352058		
					Ma09_g13580	osa:4329329	4329329		
					Ma05_g18090	osa:4344854	4344854		
					Ma06_g31540	osa:4352747	4352747		
					Ma04_g23630	osa:9267465	9267465		
					Ma10_g05100	osa:4326901	4326901		
					Ma11_g15810	osa:4330012	4330012		
					Ma04_g25530	osa:4331130	4331130		
					Ma06_g16100	osa:4341249	4341249		
					Ma06_g35980	osa:4332375	4332375		
					Ma01_g14880	osa:4343946	4343946		
					Ma09_g14110	osa:4341997	4341997		
					Ma03_g32060	osa:4334116	4334116		
					Ma10_g12270	osa:4350614	4350614		
					Ma03_g03400	osa:4341249	4341249		
					Ma09_g08470	osa:4329593	4329593		
					Ma08_g04450	osa:4348656	4348656		
					Ma04_g04720	osa:4342117	4342117		
					Novel00441	osa:4341792	4341792		
					Ma03_g06970	osa:4330843	4330843		
					Ma03_g01370	osa:4325687	4325687		
					Ma07_g01260	osa:4333566	4333566		
					Ma08_g33410	osa:4335756	4335756		
					Ma11_g13600	osa:4332563	4332563		
					Ma11_g24010	osa:4328439	4328439		
					Ma06_g28940	osa:4339718	4339718		
					Ma07_g06750	osa:4328012	4328012		
					Ma04_g07660	osa:4328124	4328124		
					Ma10_g09700	osa:4334425	4334425		
					Ma05_g27130	osa:4328684	4328684		
					Ma08_g04900	osa:4332153	4332153		
					Ma06_g15020	osa:4351698	4351698		
					Ma01_g03820	osa:4343453	4343453		
					Ma10_g16940	osa:4336153	4336153		
					Ma10_g20860	osa:4336960	4336960		
					Ma06_g19750	osa:4344496	4344496		
					Ma08_g10630	osa:4337732	4337732		
					Ma10_g14440	osa:4325563	4325563		
					Ma05_g31060	osa:4325211	4325211		

					Ma04_g22350<br>Ma06_g17860<br>Ma06_g16060<br>Ma01_g21440<br>Ma07_g28990<br>Ma07_g13520<br>Ma03_g16830<br>Ma03_g17510<br>Ma11_g24610<br>Ma03_g02700<br>Ma07_g22060<br>Ma08_g32440<br>Ma07_g11120<br>Ma10_g26330<br>Ma05_g05920<br>Ma01_g01610<br>Ma08_g33300<br>Ma09_g29570<br>Ma08_g25110<br>Ma07_g06760<br>Ma08_g01380<br>Ma04_g36760<br>Ma01_g20340<br>Ma03_g03630<br>Ma09_g06900<br>Ma01_g00130<br>Ma11_g05360<br>Ma07_g09040<br>Ma03_g29220<br>Ma10_g08510<br>Ma02_g05120<br>Ma09_g24600<br>Ma11_g15990<br>Ma03_g14690<br>Ma10_g13290<br>Ma02_g04920<br>Ma06_g10290<br>Ma06_g14670<br>Ma03_g15360<br>Ma02_g14500<br>Ma10_g11420<br>Ma08_g16120<br>Ma09_g31400<br>Ma07_g11500<br>Ma07_g12740<br>Ma10_g27820<br>Ma05_g14980<br>Ma04_g05290<br>Ma10_g28390<br>Ma01_g20350<br>Ma11_g21180<br>Ma09_g06650<br>Ma01_g14940<br>Ma06_g01700<br>Ma03_g13720<br>Ma03_g16650<br>Ma11_g02800<br>Ma05_g23890<br>Ma08_g14830<br>Ma06_g14370<br>Ma10_g12590<br>Ma03_g13910<br>Ma01_g19240<br>Ma00_g05010<br>Ma05_g05010<br>Ma06_g09160<br>Ma11_g23060<br>Ma02_g01570<br>Ma06_g00500<br>Ma04_g13550<br>Ma09_g21200<br>Ma03_g16840<br>Ma10_g20580<br>Ma10_g18290	osa:4332236<br>osa:4324261<br>osa:4341249<br>osa:4347520<br>osa:4342690<br>osa:4327301<br>osa:4340025<br>osa:4339892<br>osa:4350636<br>osa:4337818<br>osa:4336146<br>osa:4337265<br>osa:4332174<br>osa:4327178<br>osa:4340090<br>osa:4349114<br>osa:4325067<br>osa:4329101<br>osa:4336146<br>osa:4328012<br>osa:4337100<br>osa:4340251<br>osa:4325067<br>osa:9266241<br>osa:4332041<br>osa:4346656<br>osa:4338560<br>osa:4341325<br>osa:4341663<br>osa:4347717<br>osa:4340026<br>osa:4347691<br>osa:4349097<br>osa:4328073<br>osa:4344386<br>osa:4341861<br>osa:4351300<br>osa:4334535<br>osa:4338718<br>osa:4352791<br>osa:4335756<br>osa:4349897<br>osa:4348690<br>osa:4331893<br>osa:4349723<br>osa:4341247<br>osa:4342614<br>osa:4351300<br>osa:4341445<br>osa:4325067<br>osa:4336415<br>osa:4339718<br>osa:4331917<br>osa:4324914<br>osa:4331150<br>osa:4339812<br>osa:4336753<br>osa:4338560<br>osa:4332286<br>osa:4337818<br>osa:4329518<br>osa:4346726<br>osa:4341792<br>osa:4335335<br>osa:4340725<br>osa:4329532<br>osa:4343164<br>osa:4334841<br>osa:4328118<br>osa:4335430<br>osa:4328124<br>osa:4340025<br>osa:4350636<br>osa:4352146	4332236<br>4324261<br>4341249<br>4347520<br>4342690<br>4327301<br>4340025<br>4339892<br>4350636<br>4337818<br>4336146<br>4337265<br>4332174<br>4327178<br>4340090<br>4349114<br>4325067<br>4329101<br>4336146<br>4328012<br>4337100<br>4340251<br>4325067<br>9266241<br>4332041<br>4346656<br>4338560<br>4341325<br>4341663<br>4347717<br>4340026<br>4347691<br>4349097<br>4328073<br>4344386<br>4341861<br>4351300<br>4334535<br>4338718<br>4352791<br>4335756<br>4349897<br>4348690<br>4331893<br>4349723<br>4341247<br>4342614<br>4351300<br>4341445<br>4325067<br>4336415<br>4339718<br>4331917<br>4324914<br>4331150<br>4339812<br>4336753<br>4338560<br>4332286<br>4337818<br>4329518<br>4346726<br>4341792<br>4335335<br>4340725<br>4329532<br>4343164<br>4334841<br>4328118<br>4335430<br>4328124<br>4340025<br>4350636<br>4352146		
Starch and sucrose metabolism	72	145	0.00228365097945	0.0679386166387	Ma02_g23910<br>Ma04_g01750<br>Ma04_g04720<br>Ma02_g24020<br>Ma10_g28640<br>Ma07_g22060<br>Ma03_g05660<br>Ma08_g25110<br>Ma04_g18460<br>Ma08_g28660<br>Ma02_g20450<br>Ma04_g40060	osa:4343975<br>osa:4330753<br>osa:4342117<br>osa:4330753<br>osa:4340677<br>osa:4336146<br>osa:4345722<br>osa:4336146<br>osa:4330975<br>osa:4333107<br>osa:4349333<br>osa:4325861	4343975<br>4330753<br>4342117<br>4330753<br>4340677<br>4336146<br>4345722<br>4336146<br>4330975<br>4333107<br>4349333<br>4325861		

					Ma07_g11010<br>Ma09_g06900<br>Ma05_g13530<br>Ma03_g07150<br>Ma10_g15780<br>Ma05_g18650<br>Ma02_g23730<br>Ma11_g05360<br>Ma06_g29240<br>Ma03_g01820<br>Ma03_g31530<br>Ma08_g34870<br>Ma07_g28080<br>Ma04_g06090<br>Ma11_g14380<br>Ma10_g19040<br>Ma07_g06220<br>Ma09_g20960<br>Ma03_g28390<br>Ma09_g14520<br>Ma04_g39130<br>Ma07_g02790<br>Ma11_g08450<br>Ma06_g29250<br>Ma06_g04440<br>Ma08_g23170<br>Ma10_g16460<br>Ma03_g32060<br>Ma05_g08040<br>Ma05_g15200<br>Ma03_g19950<br>Ma11_g24070<br>Ma09_g06650<br>Novel01421<br>Ma08_g28360<br>Ma04_g22000<br>Ma04_g20070<br>Ma02_g20460<br>Ma03_g15100<br>Ma06_g28940<br>Ma04_g31170<br>Ma05_g23890<br>Ma05_g05600<br>Ma10_g24460<br>Ma06_g32030<br>Ma01_g00130<br>Ma09_g12560<br>Ma08_g00910<br>Ma04_g16060<br>Ma08_g15070<br>Ma06_g09160<br>Ma07_g28090<br>Ma05_g13480<br>Ma08_g23180<br>Ma10_g24940<br>Ma05_g10310<br>Ma10_g15800<br>Ma06_g16590<br>Ma10_g18290<br>Ma06_g35790	osa:4346981<br>osa:4332041<br>osa:4344268<br>osa:4347265<br>osa:4335789<br>osa:4329532<br>osa:4343910<br>osa:4338560<br>osa:4332041<br>osa:4343910<br>osa:4352146<br>osa:4352536<br>osa:4333156<br>osa:4345220<br>osa:4344268<br>osa:4330753<br>osa:4345284<br>osa:4335447<br>osa:4347903<br>osa:4347311<br>osa:4334171<br>osa:4346094<br>osa:4324962<br>osa:4332041<br>osa:4337166<br>osa:4345722<br>osa:4324962<br>osa:4334116<br>osa:4347265<br>osa:4349333<br>osa:4327285<br>osa:4341824<br>osa:4339718<br>osa:4347265<br>osa:4324621<br>osa:4335737<br>osa:4349333<br>osa:4349333<br>osa:4335447<br>osa:4339718<br>osa:4349333<br>osa:4338560<br>osa:4340579<br>osa:4330753<br>osa:4330753<br>osa:4346656<br>osa:4330753<br>osa:4346094<br>osa:4324962<br>osa:4348230<br>osa:4329532<br>osa:4333156<br>osa:4349333<br>osa:4332788<br>osa:4347311<br>osa:4347262<br>osa:4335789<br>osa:4324619<br>osa:4352146<br>osa:4324810	4346981<br>4332041<br>4344268<br>4347265<br>4335789<br>4329532<br>4343910<br>4338560<br>4332041<br>4343910<br>4352146<br>4352536<br>4333156<br>4345220<br>4344268<br>4330753<br>4345284<br>4335447<br>4347903<br>4347311<br>4334171<br>4346094<br>4324962<br>4332041<br>4337166<br>4345722<br>4324962<br>4334116<br>4347265<br>4349333<br>4327285<br>4341824<br>4339718<br>4347265<br>4324621<br>4335737<br>4349333<br>4349333<br>4335447<br>4339718<br>4349333<br>4338560<br>4340579<br>4330753<br>4330753<br>4346656<br>4330753<br>4346094<br>4324962<br>4348230<br>4329532<br>4333156<br>4349333<br>4332788<br>4347311<br>4347262<br>4335789<br>4324619<br>4352146<br>4324810		
Flavonoid biosynthesis	16	19	0.00496398077969	0.118142742557	Ma03_g05400<br>Ma09_g25700<br>Ma11_g24610<br>Ma03_g06130<br>Ma06_g09780<br>Ma10_g20600<br>Ma02_g24860<br>Ma05_g16480<br>Ma11_g04940<br>Ma06_g10250<br>Ma03_g05380<br>Ma03_g06970<br>Ma09_g13580<br>Ma08_g01380<br>Ma10_g20580<br>Ma10_g20560	osa:4329329<br>osa:4340240<br>osa:4350636<br>osa:4340240<br>osa:4350636<br>osa:4350636<br>osa:4350636<br>osa:4332311<br>osa:4348304<br>osa:4338409<br>osa:4329324<br>osa:4330843<br>osa:4329329<br>osa:4337100<br>osa:4350636<br>osa:4350636	4329329<br>4340240<br>4350636<br>4340240<br>4350636<br>4350636<br>4350636<br>4332311<br>4348304<br>4338409<br>4329324<br>4330843<br>4329329<br>4337100<br>4350636<br>4350636		
Biosynthesis of amino acids	92	207	0.00750037243971	0.148757386721	Ma08_g07480<br>Ma03_g20800<br>Ma04_g04160<br>Ma11_g08300<br>Ma07_g11340<br>Novel00240<br>Ma09_g07380<br>Ma05_g15910<br>Ma08_g06060<br>Ma11_g14450	osa:4325027<br>osa:4340547<br>osa:4337230<br>osa:4336216<br>osa:4348582<br>osa:4337230<br>osa:4332341<br>osa:4333177<br>osa:4337733<br>osa:4352839	4325027<br>4340547<br>4337230<br>4336216<br>4348582<br>4337230<br>4332341<br>4333177<br>4337733<br>4352839		

					Ma06_g21530	osa:4325444	4325444		
					Ma00_g04030	osa:4330649	4330649		
					Ma08_g28170	osa:4324655	4324655		
					Ma04_g28070	osa:4352833	4352833		
					Ma10_g11420	osa:4335756	4335756		
					Ma03_g03600	osa:4328168	4328168		
					Ma03_g16060	osa:4332151	4332151		
					Ma09_g26210	osa:4352839	4352839		
					Ma05_g00310	osa:4341792	4341792		
					Ma05_g01700	osa:4346642	4346642		
					Ma09_g20570	osa:4337406	4337406		
					Novel01132	osa:4324282	4324282		
					Ma04_g18620	osa:4334912	4334912		
					Ma07_g02100	osa:4337733	4337733		
					Ma06_g08130	osa:4352058	4352058		
					Ma03_g11800	osa:4346529	4346529		
					Ma11_g23060	osa:4343164	4343164		
					Ma08_g03130	osa:4333898	4333898		
					Novel01419	osa:4324282	4324282		
					Ma07_g10560	osa:4327301	4327301		
					Ma09_g29570	osa:4329101	4329101		
					Ma03_g32130	osa:4334101	4334101		
					Ma03_g16070	osa:4339583	4339583		
					Ma09_g07280	osa:4329858	4329858		
					Ma04_g03820	osa:4335430	4335430		
					Ma09_g09840	osa:4324282	4324282		
					Ma08_g33830	osa:4328446	4328446		
					Ma02_g18360	osa:4327301	4327301		
					Ma05_g21880	osa:4335048	4335048		
					Ma08_g14830	osa:4332286	4332286		
					Ma01_g03570	osa:4340042	4340042		
					Ma05_g27700	osa:4336216	4336216		
					Ma04_g35390	osa:4349454	4349454		
					Ma02_g08140	osa:4350964	4350964		
					Ma08_g19500	osa:4335756	4335756		
					Ma09_g12600	osa:4330747	4330747		
					Ma06_g12620	osa:4334188	4334188		
					Ma06_g01900	osa:4341792	4341792		
					Ma06_g07590	osa:4333918	4333918		
					Ma06_g26610	osa:4331941	4331941		
					Ma05_g20810	osa:4332477	4332477		
					Ma05_g31060	osa:4325211	4325211		
					Ma11_g17540	osa:4336216	4336216		
					Ma06_g38960	osa:4336353	4336353		
					Ma09_g02110	osa:4336216	4336216		
					Ma03_g21060	osa:4330649	4330649		
					Ma08_g28590	osa:4324398	4324398		
					Novel00760	osa:4324442	4324442		
					Ma06_g24910	osa:4339677	4339677		
					Ma08_g02290	osa:4338979	4338979		
					Ma09_g07020	osa:4352058	4352058		
					Ma01_g19240	osa:4341792	4341792		
					Ma01_g16200	osa:4332563	4332563		
					Ma06_g30380	osa:4329938	4329938		
					Ma04_g27940	osa:4334101	4334101		
					Ma11_g07780	osa:4341713	4341713		
					Ma07_g13520	osa:4327301	4327301		
					Ma01_g14880	osa:4343946	4343946		
					Ma05_g22300	osa:4349897	4349897		
					Ma08_g16120	osa:4349897	4349897		
					Ma11_g20920	osa:4348176	4348176		
					Novel00441	osa:4341792	4341792		
					Ma08_g33410	osa:4335756	4335756		
					Ma11_g13600	osa:4332563	4332563		
					Ma10_g07330	osa:4341713	4341713		
					Ma10_g09700	osa:4334425	4334425		
					Ma11_g07790	osa:4337230	4337230		
					Ma01_g17610	osa:4337406	4337406		
					Ma02_g13970	osa:4328168	4328168		
					Novel01203	osa:4334425	4334425		
					Ma06_g17860	osa:4324261	4324261		
					Ma03_g16830	osa:4340025	4340025		
					Ma05_g05050	osa:4340042	4340042		
					Ma03_g27870	osa:4347691	4347691		
					Ma10_g08510	osa:4347717	4347717		
					Ma02_g05120	osa:4340026	4340026		
					Ma09_g24600	osa:4347691	4347691		
					Ma07_g12740	osa:4349723	4349723		
					Ma02_g11180	osa:4339425	4339425		
					Ma02_g18420	osa:4341321	4341321		
					Ma04_g13550	osa:4335430	4335430		
					Ma03_g16840	osa:4340025	4340025		
Degradation of aromatic compounds	11	12	0.012695145403	0.198165049352	Ma03_g05400	osa:4329329	4329329		
					Ma04_g25530	osa:4331130	4331130		
					Ma08_g29910	osa:4350053	4350053		
					Ma07_g11500	osa:4331893	4331893		

					Ma09_g08110<br>Ma06_g10250<br>Ma03_g05380<br>Ma02_g09950<br>Ma09_g13580<br>Ma04_g31940<br>Ma07_g11510	osa:4350053<br>osa:4338409<br>osa:4329324<br>osa:4350053<br>osa:4329329<br>osa:4330090<br>osa:4331893	4350053<br>4338409<br>4329324<br>4350053<br>4329329<br>4330090<br>4331893		
alpha-Linolenic acid metabolism	21	33	0.0133220201245	0.198165049352	Ma08_g23400<br>Ma03_g17130<br>Ma03_g07770<br>Novel01373<br>Ma03_g11520<br>Ma08_g14010<br>Ma04_g02560<br>Ma11_g15660<br>Ma02_g24010<br>Ma03_g33110<br>Ma07_g02270<br>Ma02_g22720<br>Ma09_g12090<br>Ma04_g38880<br>Ma09_g18930<br>Ma02_g15160<br>Ma09_g15420<br>Ma07_g11670<br>Ma03_g00220<br>Ma06_g15030<br>Ma03_g13720	osa:4331824<br>osa:4333201<br>osa:4331824<br>osa:4339846<br>osa:4328603<br>osa:4331515<br>osa:4333201<br>osa:4348804<br>osa:4340986<br>osa:4333201<br>osa:4345762<br>osa:4340485<br>osa:4345994<br>osa:4334233<br>osa:4352160<br>osa:4334233<br>osa:4331824<br>osa:4333862<br>osa:4331150<br>osa:4328040<br>osa:4331150	4331824<br>4333201<br>4331824<br>4339846<br>4328603<br>4331515<br>4333201<br>4348804<br>4340986<br>4333201<br>4345762<br>4340485<br>4345994<br>4334233<br>4352160<br>4334233<br>4331824<br>4333862<br>4331150<br>4328040<br>4331150		
Amino sugar and nucleotide sugar metabolism	46	103	0.0450392112642	0.573630420867	Ma03_g03630<br>Ma05_g24340<br>Ma08_g33300<br>Ma10_g24940<br>Ma05_g29980<br>Ma10_g28640<br>Ma07_g06220<br>Ma04_g18460<br>Ma02_g04620<br>Ma01_g20320<br>Ma02_g21000<br>Ma01_g20340<br>Ma06_g31980<br>Ma08_g15070<br>Ma01_g00130<br>Ma10_g14250<br>Ma08_g33280<br>Ma10_g18160<br>Ma03_g31530<br>Ma07_g16390<br>Ma07_g28080<br>Ma11_g23980<br>Ma09_g14520<br>Ma07_g02790<br>Ma11_g08450<br>Ma04_g23630<br>Ma06_g04440<br>Ma10_g16460<br>Ma01_g20350<br>Ma09_g06650<br>Ma03_g15360<br>Ma06_g28940<br>Ma08_g33290<br>Ma05_g05600<br>Ma06_g28330<br>Ma09_g24170<br>Ma04_g02590<br>Ma03_g16650<br>Ma08_g00910<br>Ma04_g16060<br>Ma06_g01670<br>Ma07_g28090<br>Ma09_g23530<br>Ma06_g01700<br>Ma10_g18290<br>Ma08_g28660	osa:9266241<br>osa:4341967<br>osa:4325067<br>osa:4347311<br>osa:4349683<br>osa:4340677<br>osa:4345284<br>osa:4330975<br>osa:4340050<br>osa:4327172<br>osa:4332373<br>osa:4325067<br>osa:4350476<br>osa:4348230<br>osa:4346656<br>osa:4332425<br>osa:4325067<br>osa:4332014<br>osa:4352146<br>osa:4350476<br>osa:4333156<br>osa:4344934<br>osa:4347311<br>osa:4346094<br>osa:4324962<br>osa:9267465<br>osa:4337166<br>osa:4324962<br>osa:4325067<br>osa:4339718<br>osa:4338718<br>osa:4339718<br>osa:4325067<br>osa:4340579<br>osa:4339812<br>osa:4337437<br>osa:4332425<br>osa:4339812<br>osa:4346094<br>osa:4324962<br>osa:4338802<br>osa:4333156<br>osa:4336928<br>osa:4324914<br>osa:4352146<br>osa:4333107	9266241<br>4341967<br>4325067<br>4347311<br>4349683<br>4340677<br>4345284<br>4330975<br>4340050<br>4327172<br>4332373<br>4325067<br>4350476<br>4348230<br>4346656<br>4332425<br>4325067<br>4332014<br>4352146<br>4350476<br>4333156<br>4344934<br>4347311<br>4346094<br>4324962<br>9267465<br>4337166<br>4324962<br>4325067<br>4339718<br>4338718<br>4339718<br>4325067<br>4340579<br>4339812<br>4337437<br>4332425<br>4339812<br>4346094<br>4324962<br>4338802<br>4333156<br>4336928<br>4324914<br>4352146<br>4333107		
Zeatin biosynthesis	10	14	0.0482042370477	0.573630420867	Ma05_g21640<br>Ma07_g08820<br>Ma09_g21880<br>Ma04_g23150<br>Ma08_g03900<br>Ma03_g08660<br>Ma08_g23380<br>Ma01_g02270<br>Ma04_g21780<br>Ma09_g16350	osa:4348932<br>osa:4327887<br>osa:4339535<br>osa:4336629<br>osa:4348932<br>osa:4326515<br>osa:4326515<br>osa:4342142<br>osa:4348932<br>osa:4326515	4348932<br>4327887<br>4339535<br>4336629<br>4348932<br>4326515<br>4326515<br>4342142<br>4348932<br>4326515		

Carotenoid biosynthesis	15	26	0.056975480684	0.616371109218	Ma11_g07140<br>Ma11_g02800<br>Ma09_g20310<br>Ma10_g17210<br>Ma04_g28360<br>Ma04_g00620<br>Ma08_g16510<br>Ma06_g13460<br>Ma07_g01260<br>Ma04_g29400<br>Ma02_g14780<br>Ma04_g38030<br>Ma06_g14670<br>Ma09_g14710<br>Ma08_g11970	osa:4342137<br>osa:4336753<br>osa:4331152<br>osa:4352042<br>osa:4347261<br>osa:4335984<br>osa:4331854<br>osa:4352042<br>osa:4333566<br>osa:4333566<br>osa:4333566<br>osa:4333566<br>osa:4334535<br>osa:4347261<br>osa:4336753	4342137<br>4336753<br>4331152<br>4352042<br>4347261<br>4335984<br>4331854<br>4352042<br>4333566<br>4333566<br>4333566<br>4333566<br>4334535<br>4347261<br>4336753		
Cysteine and methionine metabolism	38	85	0.0631406540487	0.618522934881	Ma06_g22100<br>Ma10_g16100<br>Ma07_g27160<br>Ma07_g02100<br>Ma06_g14390<br>Ma03_g02700<br>Ma07_g20920<br>Ma09_g29570<br>Ma09_g19150<br>Ma07_g06760<br>Ma06_g05530<br>Ma03_g32130<br>Ma06_g14410<br>Ma05_g25700<br>Ma09_g13000<br>Ma10_g25720<br>Ma04_g01300<br>Ma06_g31540<br>Ma08_g06060<br>Ma03_g04640<br>Ma04_g27940<br>Ma11_g14450<br>Ma08_g28170<br>Ma04_g28070<br>Ma08_g04450<br>Ma09_g01600<br>Ma05_g21880<br>Ma02_g11180<br>Ma06_g14400<br>Ma06_g12620<br>Ma07_g06750<br>Ma06_g14370<br>Ma02_g18420<br>Ma06_g26610<br>Ma09_g26210<br>Ma05_g01700<br>Ma04_g06200<br>Ma05_g26570	osa:4350524<br>osa:4337818<br>osa:4331707<br>osa:4337733<br>osa:4337818<br>osa:4337818<br>osa:4329955<br>osa:4329101<br>osa:4336750<br>osa:4328012<br>osa:4329955<br>osa:4334101<br>osa:4337818<br>osa:4327423<br>osa:4347103<br>osa:4345657<br>osa:4352871<br>osa:4352747<br>osa:4337733<br>osa:4329955<br>osa:4334101<br>osa:4352839<br>osa:4324655<br>osa:4352833<br>osa:4348656<br>osa:4350455<br>osa:4335048<br>osa:4339425<br>osa:4337818<br>osa:4334188<br>osa:4328012<br>osa:4337818<br>osa:4341321<br>osa:4331941<br>osa:4352839<br>osa:4346642<br>osa:4342996<br>osa:4329955	4350524<br>4337818<br>4331707<br>4337733<br>4337818<br>4337818<br>4329955<br>4329101<br>4336750<br>4328012<br>4329955<br>4334101<br>4337818<br>4327423<br>4347103<br>4345657<br>4352871<br>4352747<br>4337733<br>4329955<br>4334101<br>4352839<br>4324655<br>4352833<br>4348656<br>4350455<br>4335048<br>4339425<br>4337818<br>4334188<br>4328012<br>4337818<br>4341321<br>4331941<br>4352839<br>4346642<br>4342996<br>4329955		
Glycolysis / Gluconeogenesis	50	119	0.0747531448936	0.618522934881	Ma08_g07480<br>Ma10_g12590<br>Ma08_g29910<br>Ma07_g13520<br>Ma05_g00310<br>Ma10_g28640<br>Ma04_g25530<br>Ma11_g08300<br>Ma07_g11340<br>Ma05_g30490<br>Ma07_g10560<br>Ma07_g06760<br>Ma09_g11280<br>Ma08_g15070<br>Ma08_g33830<br>Ma07_g12740<br>Ma11_g20920<br>Ma03_g27870<br>Ma03_g06120<br>Ma09_g24600<br>Ma02_g18360<br>Novel00441<br>Ma09_g14520<br>Ma01_g19240<br>Ma01_g03570<br>Ma08_g32440<br>Ma07_g11500<br>Ma11_g16750<br>Ma05_g22300<br>Ma08_g16120<br>Ma05_g27700<br>Ma09_g08110<br>Ma03_g09880	osa:4325027<br>osa:4329518<br>osa:4350053<br>osa:4327301<br>osa:4341792<br>osa:4340677<br>osa:4331130<br>osa:4336216<br>osa:4348582<br>osa:4332519<br>osa:4327301<br>osa:4328012<br>osa:4348138<br>osa:4348230<br>osa:4328446<br>osa:4349723<br>osa:4348176<br>osa:4347691<br>osa:4340725<br>osa:4347691<br>osa:4327301<br>osa:4341792<br>osa:4347311<br>osa:4341792<br>osa:4340042<br>osa:4337265<br>osa:4331893<br>osa:4341770<br>osa:4349897<br>osa:4349897<br>osa:4336216<br>osa:4350053<br>osa:4332519	4325027<br>4329518<br>4350053<br>4327301<br>4341792<br>4340677<br>4331130<br>4336216<br>4348582<br>4332519<br>4327301<br>4328012<br>4348138<br>4348230<br>4328446<br>4349723<br>4348176<br>4347691<br>4340725<br>4347691<br>4327301<br>4341792<br>4347311<br>4341792<br>4340042<br>4337265<br>4331893<br>4341770<br>4349897<br>4349897<br>4336216<br>4350053<br>4332519		

					Ma02_g09950<br>Ma11_g17540<br>Ma04_g35390<br>Ma04_g02250<br>Ma07_g11510<br>Ma09_g12600<br>Ma06_g01900<br>Ma07_g06750<br>Ma05_g31060<br>Ma08_g08880<br>Ma05_g05050<br>Ma05_g05010<br>Ma09_g20570<br>Ma10_g24940<br>Ma09_g02110<br>Ma01_g17610<br>Ma04_g31940	osa:4350053<br>osa:4336216<br>osa:4349454<br>osa:4337265<br>osa:4331893<br>osa:4330747<br>osa:4341792<br>osa:4328012<br>osa:4325211<br>osa:4346159<br>osa:4340042<br>osa:4340725<br>osa:4337406<br>osa:4347311<br>osa:4336216<br>osa:4337406<br>osa:4330090	4350053<br>4336216<br>4349454<br>4337265<br>4331893<br>4330747<br>4341792<br>4328012<br>4325211<br>4346159<br>4340042<br>4340725<br>4337406<br>4347311<br>4336216<br>4337406<br>4330090		
Pentose and glucuronate interconversions	20	41	0.0899779708502	0.618522934881	Ma05_g13530<br>Ma05_g05010<br>Ma08_g23170<br>Ma05_g24340<br>Ma03_g05660<br>Ma08_g34870<br>Ma04_g26280<br>Ma04_g33260<br>Ma03_g31530<br>Ma03_g06120<br>Ma07_g28080<br>Ma07_g28090<br>Ma03_g19950<br>Ma03_g13710<br>Ma03_g08830<br>Ma04_g40060<br>Ma03_g00210<br>Ma11_g14380<br>Ma10_g18290<br>Ma04_g26260	osa:4344268<br>osa:4340725<br>osa:4345722<br>osa:4341967<br>osa:4345722<br>osa:4352536<br>osa:4346217<br>osa:4335013<br>osa:4352146<br>osa:4340725<br>osa:4333156<br>osa:4333156<br>osa:4327285<br>osa:4335013<br>osa:4344231<br>osa:4325861<br>osa:4335013<br>osa:4344268<br>osa:4352146<br>osa:4346217	4344268<br>4340725<br>4345722<br>4341967<br>4345722<br>4352536<br>4346217<br>4335013<br>4352146<br>4340725<br>4333156<br>4333156<br>4327285<br>4335013<br>4344231<br>4325861<br>4335013<br>4344268<br>4352146<br>4346217		
Valine, leucine and isoleucine degradation	18	36	0.0909306505528	0.618522934881	Ma11_g21350<br>Ma03_g12070<br>Ma08_g15550<br>Ma11_g21360<br>Ma03_g00220<br>Ma04_g10090<br>Ma04_g28920<br>Ma03_g06120<br>Ma05_g05010<br>Ma08_g30510<br>Ma04_g09570<br>Ma08_g20630<br>Ma11_g15660<br>Ma08_g25460<br>Ma01_g14700<br>Ma03_g13720<br>Ma03_g16070<br>Ma03_g16060	osa:4330184<br>osa:4331418<br>osa:4351929<br>osa:4330184<br>osa:4331150<br>osa:4326266<br>osa:4342610<br>osa:4340725<br>osa:4340725<br>osa:4344267<br>osa:4352741<br>osa:4342508<br>osa:4348804<br>osa:4352741<br>osa:4345605<br>osa:4331150<br>osa:4339583<br>osa:4332151	4330184<br>4331418<br>4351929<br>4330184<br>4331150<br>4326266<br>4342610<br>4340725<br>4340725<br>4344267<br>4352741<br>4342508<br>4348804<br>4352741<br>4345605<br>4331150<br>4339583<br>4332151		
Thiamine metabolism	7	10	0.0979543332004	0.618522934881	Ma04_g30490<br>Ma05_g14980<br>Ma06_g35590<br>Ma08_g09930<br>Ma03_g26140<br>Ma02_g15890<br>Ma05_g05920	osa:4343443<br>osa:4342614<br>osa:4333719<br>osa:4326818<br>osa:4338768<br>osa:4343443<br>osa:4340090	4343443<br>4342614<br>4333719<br>4326818<br>4338768<br>4343443<br>4340090		
Metabolic pathways	534	1549	0.0982383032286	0.618522934881	Ma03_g20800<br>Ma10_g18160<br>Ma10_g28640<br>Ma04_g22620<br>Ma02_g24010<br>Ma09_g06640<br>Ma02_g21000<br>Ma04_g38030<br>Ma04_g37810<br>Ma04_g14940<br>Ma08_g33830<br>Ma09_g20310<br>Ma08_g02290<br>Ma04_g06090<br>Ma08_g06060<br>Ma06_g17200<br>Ma09_g30540<br>Ma11_g14450<br>Ma07_g02790<br>Ma00_g04030<br>Ma11_g07140<br>Ma01_g21520<br>Ma07_g17020	osa:4340547<br>osa:4332014<br>osa:4340677<br>osa:4339874<br>osa:4340986<br>osa:4324599<br>osa:4332373<br>osa:4333566<br>osa:4333932<br>osa:4324599<br>osa:4328446<br>osa:4331152<br>osa:4338979<br>osa:4345220<br>osa:4337733<br>osa:4344386<br>osa:4349859<br>osa:4352839<br>osa:4346094<br>osa:4330649<br>osa:4342137<br>osa:4344496<br>osa:4335701	4340547<br>4332014<br>4340677<br>4339874<br>4340986<br>4324599<br>4332373<br>4333566<br>4333932<br>4324599<br>4328446<br>4331152<br>4338979<br>4345220<br>4337733<br>4344386<br>4349859<br>4352839<br>4346094<br>4330649<br>4342137<br>4344496<br>4335701		

					Ma03_g00220	osa:4331150	4331150		
					Ma10_g17210	osa:4352042	4352042		
					Ma02_g22450	osa:4324557	4324557		
					Ma05_g26570	osa:4329955	4329955		
					Ma05_g21880	osa:4335048	4335048		
					Novel01421	osa:4347265	4347265		
					Ma05_g21330	osa:4327981	4327981		
					Ma03_g16060	osa:4332151	4332151		
					Ma07_g10180	osa:4350917	4350917		
					Ma02_g05150	osa:4340008	4340008		
					Ma05_g05600	osa:4340579	4340579		
					Ma06_g28330	osa:4339812	4339812		
					Ma05_g00310	osa:4341792	4341792		
					Ma04_g28920	osa:4342610	4342610		
					Ma06_g14310	osa:4351698	4351698		
					Ma07_g28090	osa:4333156	4333156		
					Ma03_g28390	osa:4347903	4347903		
					Ma04_g18620	osa:4334912	4334912		
					Ma10_g16100	osa:4337818	4337818		
					mito3_g00150	osa:6450138	6450138		
					Ma08_g03130	osa:4333898	4333898		
					Ma08_g05390	osa:4327833	4327833		
					Ma04_g16060	osa:4324962	4324962		
					Ma06_g05530	osa:4329955	4329955		
					Ma03_g27870	osa:4347691	4347691		
					Ma06_g32030	osa:4330753	4330753		
					Ma11_g16750	osa:4341770	4341770		
					Ma04_g10150	osa:4345689	4345689		
					Ma09_g07280	osa:4329858	4329858		
					Ma04_g04160	osa:4337230	4337230		
					Ma08_g20630	osa:4342508	4342508		
					Ma09_g09840	osa:4324282	4324282		
					Ma05_g04020	osa:4336116	4336116		
					Ma11_g20450	osa:4349278	4349278		
					Ma10_g16940	osa:4336153	4336153		
					Ma03_g14310	osa:4346332	4346332		
					Ma06_g04440	osa:4337166	4337166		
					Ma03_g27370	osa:4347947	4347947		
					Ma07_g05720	osa:4352511	4352511		
					Ma04_g33980	osa:4330413	4330413		
					Ma11_g24070	osa:4341824	4341824		
					Ma01_g17610	osa:4337406	4337406		
					Ma05_g24340	osa:4341967	4341967		
					Ma11_g15990	osa:4349097	4349097		
					Ma02_g08140	osa:4350964	4350964		
					Ma11_g21350	osa:4330184	4330184		
					Ma06_g01900	osa:4341792	4341792		
					Ma07_g27160	osa:4331707	4331707		
					Ma06_g11320	osa:4325687	4325687		
					Ma05_g27310	osa:4347520	4347520		
					Ma08_g04920	osa:4332153	4332153		
					Ma07_g15870	osa:4328326	4328326		
					Ma08_g24890	osa:4330711	4330711		
					Ma08_g28590	osa:4324398	4324398		
					Novel00760	osa:4324442	4324442		
					Ma03_g17130	osa:4333201	4333201		
					Ma01_g16340	osa:4339248	4339248		
					Ma06_g24910	osa:4339677	4339677		
					Ma04_g00620	osa:4335984	4335984		
					Ma06_g31540	osa:4352747	4352747		
					Ma05_g31950	osa:4351300	4351300		
					Ma09_g07020	osa:4352058	4352058		
					Ma09_g19150	osa:4336750	4336750		
					Ma05_g02000	osa:4334276	4334276		
					Ma04_g07830	osa:4327859	4327859		
					Novel01203	osa:4334425	4334425		
					Ma10_g25840	osa:4332174	4332174		
					Ma08_g14810	osa:4349044	4349044		
					Ma09_g13580	osa:4329329	4329329		
					Ma04_g31170	osa:4349333	4349333		
					Ma07_g24600	osa:4324933	4324933		
					Ma08_g25460	osa:4352741	4352741		
					Ma07_g25460	osa:4325937	4325937		
					Ma08_g02270	osa:4332054	4332054		
					Ma03_g11520	osa:4328603	4328603		
					Ma06_g19810	osa:4338825	4338825		
					Ma08_g09930	osa:4326818	4326818		
					Ma03_g33110	osa:4333201	4333201		
					Ma11_g20920	osa:4348176	4348176		
					Ma04_g04720	osa:4342117	4342117		
					Ma07_g20920	osa:4329955	4329955		
					Ma04_g07920	osa:4324599	4324599		
					Ma03_g05860	osa:4329701	4329701		
					Ma10_g10490	osa:4329593	4329593		
					Ma10_g24460	osa:4330753	4330753		
					Ma01_g03820	osa:4343453	4343453		
					Ma03_g27120	osa:4326346	4326346		

					Ma10_g20860	osa:4336960	4336960		
					Ma05_g31060	osa:4325211	4325211		
					Ma02_g23910	osa:4343975	4343975		
					Ma02_g13970	osa:4328168	4328168		
					Ma04_g13410	osa:4337284	4337284		
					Ma08_g27570	osa:4334073	4334073		
					Ma06_g16100	osa:4341249	4341249		
					Ma07_g22060	osa:4336146	4336146		
					Ma01_g04250	osa:4332745	4332745		
					Ma07_g28480	osa:4352223	4352223		
					Ma10_g08510	osa:4347717	4347717		
					Ma02_g05120	osa:4340026	4340026		
					Ma06_g30010	osa:4344539	4344539		
					Ma06_g10290	osa:4351300	4351300		
					Ma06_g14670	osa:4334535	4334535		
					Ma02_g14500	osa:4352791	4352791		
					Ma10_g24730	osa:4329733	4329733		
					Ma07_g11500	osa:4331893	4331893		
					Ma06_g10670	osa:4347680	4347680		
					Ma05_g29560	osa:4351119	4351119		
					Ma08_g29310	osa:4329889	4329889		
					Ma01_g14940	osa:4331917	4331917		
					Ma06_g37750	osa:4327647	4327647		
					Ma03_g15100	osa:4335447	4335447		
					Ma04_g30490	osa:4343443	4343443		
					Ma02_g18420	osa:4341321	4341321		
					Ma09_g15520	osa:4345606	4345606		
					Ma07_g02100	osa:4337733	4337733		
					Ma05_g05010	osa:4340725	4340725		
					Ma06_g09160	osa:4329532	4329532		
					Ma02_g01570	osa:4334841	4334841		
					Ma09_g08810	osa:4345212	4345212		
					Ma08_g07480	osa:4325027	4325027		
					Ma04_g01750	osa:4330753	4330753		
					Ma07_g28490	osa:4352223	4352223		
					Ma10_g15940	osa:4324554	4324554		
					Ma10_g11420	osa:4335756	4335756		
					Ma07_g11340	osa:4348582	4348582		
					Ma02_g15160	osa:4334233	4334233		
					Ma02_g04620	osa:4340050	4340050		
					Ma02_g23730	osa:4343910	4343910		
					Ma10_g15780	osa:4335789	4335789		
					Ma06_g14410	osa:4337818	4337818		
					Ma02_g03830	osa:4338750	4338750		
					Ma04_g36700	osa:4348876	4348876		
					Ma04_g07860	osa:4327859	4327859		
					Ma07_g06700	osa:4349111	4349111		
					Ma11_g10950	osa:4344095	4344095		
					Ma03_g06120	osa:4340725	4340725		
					Ma08_g04770	osa:4334003	4334003		
					Ma06_g01540	osa:4346185	4346185		
					Ma10_g19040	osa:4330753	4330753		
					Ma05_g20350	osa:4334308	4334308		
					Ma02_g24020	osa:4330753	4330753		
					Ma09_g27580	osa:4345212	4345212		
					Ma04_g10990	osa:4343681	4343681		
					Ma03_g01820	osa:4343910	4343910		
					Ma04_g02560	osa:4333201	4333201		
					Ma04_g12630	osa:4341663	4341663		
					Ma04_g28070	osa:4352833	4352833		
					Ma03_g19950	osa:4327285	4327285		
					Ma10_g10450	osa:4349835	4349835		
					Ma04_g02250	osa:4337265	4337265		
					Ma08_g15550	osa:4351929	4351929		
					Ma03_g28580	osa:4339885	4339885		
					Ma08_g08880	osa:4346159	4346159		
					Ma09_g29260	osa:4340093	4340093		
					Ma10_g20600	osa:4350636	4350636		
					Ma08_g06070	osa:4352549	4352549		
					Ma10_g24940	osa:4347311	4347311		
					Ma05_g10310	osa:4347262	4347262		
					Ma10_g23920	osa:4345689	4345689		
					Ma08_g29910	osa:4350053	4350053		
					Ma05_g23060	osa:4349318	4349318		
					Novel01419	osa:4324282	4324282		
					Ma07_g10560	osa:4327301	4327301		
					Ma04_g26360	osa:4330302	4330302		
					Ma03_g16070	osa:4339583	4339583		
					Ma10_g14640	osa:4331360	4331360		
					Ma05_g25700	osa:4327423	4327423		
					Ma10_g09000	osa:4331914	4331914		
					Ma08_g00920	osa:4337297	4337297		
					Ma04_g20190	osa:4331295	4331295		
					Ma07_g28080	osa:4333156	4333156		
					Ma11_g15760	osa:4343080	4343080		
					Ma06_g10250	osa:4338409	4338409		
					Ma06_g29250	osa:4332041	4332041		

					Ma06_g09780	osa:4350636	4350636		
					Ma10_g12550	osa:4336044	4336044		
					Ma05_g26380	osa:4347160	4347160		
					Ma05_g27700	osa:4336216	4336216		
					Ma09_g01600	osa:4350455	4350455		
					Ma04_g19680	osa:4333020	4333020		
					Ma07_g11510	osa:4331893	4331893		
					Ma03_g12070	osa:4331418	4331418		
					Ma03_g05400	osa:4329329	4329329		
					Ma05_g20810	osa:4332477	4332477		
					Ma09_g24170	osa:4337437	4337437		
					Ma04_g25940	osa:4345286	4345286		
					Ma11_g17540	osa:4336216	4336216		
					Ma06_g23970	osa:4326635	4326635		
					Ma10_g21000	osa:4325336	4325336		
					Ma06_g38960	osa:4336353	4336353		
					Ma09_g02110	osa:4336216	4336216		
					Ma05_g08470	osa:4334266	4334266		
					Ma03_g21060	osa:4330649	4330649		
					Ma11_g04940	osa:4348304	4348304		
					Ma10_g20580	osa:4350636	4350636		
					Ma03_g23680	osa:4324793	4324793		
					Ma11_g21360	osa:4330184	4330184		
					Ma11_g19670	osa:4334994	4334994		
					Ma01_g16200	osa:4332563	4332563		
					Ma10_g26480	osa:4351226	4351226		
					Ma06_g30380	osa:4329938	4329938		
					Ma04_g27940	osa:4334101	4334101		
					Ma11_g07780	osa:4341713	4341713		
					Ma00_g01820	osa:4330439	4330439		
					Ma08_g23170	osa:4345722	4345722		
					Ma05_g05050	osa:4340042	4340042		
					Ma05_g22300	osa:4349897	4349897		
					Ma03_g03400	osa:4341249	4341249		
					Ma10_g26890	osa:4327647	4327647		
					Ma07_g01260	osa:4333566	4333566		
					Ma05_g18380	osa:4337563	4337563		
					Ma11_g24010	osa:4328439	4328439		
					Ma09_g25530	osa:4325633	4325633		
					Ma05_g27130	osa:4328684	4328684		
					Ma05_g13480	osa:4349333	4349333		
					Ma04_g18600	osa:4331075	4331075		
					Ma06_g16060	osa:4341249	4341249		
					Ma07_g28990	osa:4342690	4342690		
					Ma06_g17860	osa:4324261	4324261		
					Ma08_g04460	osa:4340334	4340334		
					Novel01453	osa:6450140	6450140		
					Ma06_g35980	osa:4332375	4332375		
					Ma00_g01500	osa:4351694	4351694		
					Ma01_g14880	osa:4343946	4343946		
					Ma03_g07150	osa:4347265	4347265		
					Ma03_g29220	osa:4341663	4341663		
					Ma09_g23510	osa:4341751	4341751		
					Ma02_g15890	osa:4343443	4343443		
					Ma06_g19820	osa:4350171	4350171		
					Ma08_g16120	osa:4349897	4349897		
					Ma04_g20070	osa:4349333	4349333		
					Ma07_g12740	osa:4349723	4349723		
					Ma03_g32890	osa:4326769	4326769		
					Ma10_g12210	osa:4350591	4350591		
					Ma04_g05290	osa:4351300	4351300		
					Ma02_g11180	osa:4339425	4339425		
					Ma04_g22000	osa:4335737	4335737		
					Ma03_g21780	osa:4339546	4339546		
					Ma05_g23890	osa:4338560	4338560		
					Ma05_g16030	osa:4343288	4343288		
					Ma03_g13910	osa:4346726	4346726		
					Ma00_g05010	osa:4335335	4335335		
					Ma10_g13290	osa:4344386	4344386		
					Ma04_g13550	osa:4335430	4335430		
					Ma03_g33280	osa:4339675	4339675		
					Ma01_g07600	osa:4334073	4334073		
					Ma06_g21190	osa:4337048	4337048		
					Ma06_g08980	osa:4351698	4351698		
					Ma06_g19750	osa:4344496	4344496		
					Ma01_g08380	osa:4347745	4347745		
					Ma09_g19460	osa:4342702	4342702		
					Ma01_g07340	osa:4339593	4339593		
					Ma08_g34680	osa:4328991	4328991		
					Novel00240	osa:4337230	4337230		
					Ma06_g30180	osa:4350358	4350358		
					Ma09_g11280	osa:4348138	4348138		
					Ma04_g11720	osa:4347802	4347802		
					Ma06_g32150	osa:4329733	4329733		
					Ma10_g25720	osa:4345657	4345657		
					Ma05_g15910	osa:4333177	4333177		
					Ma06_g09580	osa:4346136	4346136		

					Ma10_g21340	osa:4338007	4338007		
					Ma06_g21100	osa:4337051	4337051		
					Ma06_g28550	osa:4345387	4345387		
					Ma06_g21530	osa:4325444	4325444		
					Ma08_g23400	osa:4331824	4331824		
					Ma05_g01630	osa:4352670	4352670		
					Ma10_g14420	osa:4338088	4338088		
					Ma09_g20960	osa:4335447	4335447		
					Ma03_g01410	osa:9268279	9268279		
					Ma09_g03520	osa:4332372	4332372		
					Ma08_g16510	osa:4331854	4331854		
					Ma09_g28900	osa:4338045	4338045		
					Ma03_g25580	osa:4351119	4351119		
					Ma04_g29400	osa:4333566	4333566		
					Ma10_g27460	osa:4327329	4327329		
					Ma06_g14400	osa:4337818	4337818		
					Ma08_g34870	osa:4352536	4352536		
					Ma10_g20560	osa:4350636	4350636		
					Ma06_g16830	osa:4324526	4324526		
					Ma04_g11650	osa:4324809	4324809		
					Ma09_g26210	osa:4352839	4352839		
					Ma03_g06130	osa:4340240	4340240		
					Ma05_g01700	osa:4346642	4346642		
					Novel01132	osa:4324282	4324282		
					Ma04_g31940	osa:4330090	4330090		
					Ma04_g10090	osa:4326266	4326266		
					Ma06_g27690	osa:4351671	4351671		
					Ma06_g08130	osa:4352058	4352058		
					Ma00_g01810	osa:4330439	4330439		
					Ma09_g25040	osa:4343570	4343570		
					Ma05_g12640	osa:4349006	4349006		
					Ma06_g35790	osa:4324810	4324810		
					Ma08_g29090	osa:4336162	4336162		
					Ma05_g05920	osa:4340090	4340090		
					Ma09_g29570	osa:4329101	4329101		
					Ma07_g11670	osa:4333862	4333862		
					Ma02_g04920	osa:4341861	4341861		
					Ma07_g18790	osa:4347745	4347745		
					Ma08_g28360	osa:4324621	4324621		
					Ma08_g14010	osa:4331515	4331515		
					Ma05_g10890	osa:4334626	4334626		
					Ma05_g08040	osa:4347265	4347265		
					Ma02_g18360	osa:4327301	4327301		
					Ma08_g21280	osa:4329007	4329007		
					Ma03_g04640	osa:4329955	4329955		
					Ma09_g14520	osa:4347311	4347311		
					Ma06_g01670	osa:4338802	4338802		
					Ma09_g31420	osa:4347851	4347851		
					Ma08_g14830	osa:4332286	4332286		
					Ma01_g03570	osa:4340042	4340042		
					Ma04_g13500	osa:4324151	4324151		
					Ma09_g23330	osa:4328485	4328485		
					Ma08_g02250	osa:4332372	4332372		
					Ma02_g24860	osa:4350636	4350636		
					Ma11_g24380	osa:4331065	4331065		
					Ma05_g30490	osa:4332519	4332519		
					Ma05_g23990	osa:4329497	4329497		
					Ma04_g35390	osa:4349454	4349454		
					Ma03_g00730	osa:9266710	9266710		
					Ma02_g20460	osa:4349333	4349333		
					Ma08_g19500	osa:4335756	4335756		
					Ma06_g12620	osa:4334188	4334188		
					Ma03_g13760	osa:4334994	4334994		
					Ma06_g07590	osa:4333918	4333918		
					Ma06_g26610	osa:4331941	4331941		
					Ma02_g14780	osa:4333566	4333566		
					Ma08_g00910	osa:4346094	4346094		
					Ma10_g21240	osa:4338045	4338045		
					Ma09_g03480	osa:4342171	4342171		
					Ma01_g02270	osa:4342142	4342142		
					Ma06_g24830	osa:3131463	3131463		
					Ma09_g19700	osa:4345078	4345078		
					Ma07_g22000	osa:4345962	4345962		
					Ma09_g08110	osa:4350053	4350053		
					Ma02_g22720	osa:4340485	4340485		
					Ma08_g29610	osa:4329692	4329692		
					Ma04_g18460	osa:4330975	4330975		
					Ma03_g05380	osa:4329324	4329324		
					Ma01_g19240	osa:4341792	4341792		
					Ma05_g18650	osa:4329532	4329532		
					Ma03_g31530	osa:4352146	4352146		
					Ma04_g01300	osa:4352871	4352871		
					Ma06_g35590	osa:4333719	4333719		
					Novel00441	osa:4341792	4341792		
					Ma10_g05100	osa:4326901	4326901		
					Ma09_g03470	osa:4332375	4332375		
					Ma10_g16460	osa:4324962	4324962		

					Ma03_g32060	osa:4334116	4334116		
					Ma11_g23980	osa:4344934	4344934		
					Ma08_g13290	osa:4330077	4330077		
					Ma07_g26160	osa:4326680	4326680		
					Ma09_g03450	osa:4332375	4332375		
					Ma09_g14110	osa:4341997	4341997		
					Ma01_g14700	osa:4345605	4345605		
					Ma08_g33410	osa:4335756	4335756		
					Ma10_g07330	osa:4341713	4341713		
					Ma07_g06750	osa:4328012	4328012		
					Ma08_g04900	osa:4332153	4332153		
					Ma03_g00940	osa:4346459	4346459		
					Ma09_g24200	osa:4349012	4349012		
					Ma11_g07790	osa:4337230	4337230		
					Ma04_g38880	osa:4334233	4334233		
					Ma09_g21880	osa:4339535	4339535		
					Ma01_g21440	osa:4347520	4347520		
					Ma05_g03450	osa:4330413	4330413		
					Ma09_g31410	osa:4347851	4347851		
					Ma03_g16830	osa:4340025	4340025		
					Ma11_g24610	osa:4350636	4350636		
					Ma08_g32440	osa:4337265	4337265		
					Ma07_g02270	osa:4345762	4345762		
					Ma07_g06760	osa:4328012	4328012		
					Ma08_g25110	osa:4336146	4336146		
					Ma04_g39070	osa:4352670	4352670		
					Ma05_g13530	osa:4344268	4344268		
					Ma01_g00130	osa:4346656	4346656		
					Ma07_g09040	osa:4341325	4341325		
					Ma09_g06900	osa:4332041	4332041		
					Ma06_g22930	osa:3131406	3131406		
					Ma04_g09570	osa:4352741	4352741		
					Ma04_g18630	osa:4330910	4330910		
					Ma10_g27820	osa:4341247	4341247		
					Ma05_g14980	osa:4342614	4342614		
					Ma04_g06200	osa:4342996	4342996		
					Ma03_g01840	osa:4343908	4343908		
					Ma11_g21180	osa:4336415	4336415		
					Ma09_g06650	osa:4339718	4339718		
					Ma09_g12090	osa:4345994	4345994		
					Ma03_g13720	osa:4331150	4331150		
					Ma06_g14370	osa:4337818	4337818		
					Ma11_g05360	osa:4338560	4338560		
					Ma09_g12560	osa:4330753	4330753		
					Ma05_g23550	osa:4344180	4344180		
					Ma06_g00500	osa:4328118	4328118		
					Ma06_g01700	osa:4324914	4324914		
					Ma10_g17260	osa:4343523	4343523		
					Ma04_g37240	osa:4327731	4327731		
					Ma11_g19280	osa:4344045	4344045		
					Ma06_g14390	osa:4337818	4337818		
					Ma11_g08300	osa:4336216	4336216		
					Ma03_g05660	osa:4345722	4345722		
					Ma02_g07020	osa:4347074	4347074		
					Ma07_g24940	osa:4344470	4344470		
					Ma04_g26260	osa:4346217	4346217		
					Ma04_g39490	osa:4331021	4331021		
					Ma09_g07380	osa:4332341	4332341		
					Ma06_g24480	osa:4332771	4332771		
					Ma11_g21050	osa:4330016	4330016		
					Ma01_g10240	osa:4324933	4324933		
					Ma05_g09350	osa:4339457	4339457		
					Ma11_g15660	osa:4348804	4348804		
					Ma08_g25440	osa:4328260	4328260		
					Ma08_g28170	osa:4324655	4324655		
					Ma01_g04420	osa:4336415	4336415		
					Ma07_g09220	osa:4341249	4341249		
					Ma09_g30120	osa:4337482	4337482		
					Ma10_g23010	osa:4337696	4337696		
					Ma06_g13460	osa:4352042	4352042		
					Ma09_g13000	osa:4347103	4347103		
					Ma08_g11970	osa:4336753	4336753		
					Ma11_g17480	osa:4351008	4351008		
					Ma01_g08090	osa:4327647	4327647		
					Ma09_g25700	osa:4340240	4340240		
					Ma03_g16650	osa:4339812	4339812		
					Ma03_g26140	osa:4338768	4338768		
					Ma09_g20570	osa:4337406	4337406		
					Ma09_g15420	osa:4331824	4331824		
					Ma06_g15020	osa:4351698	4351698		
					Ma05_g16190	osa:4331917	4331917		
					Ma08_g05120	osa:4342988	4342988		
					Ma03_g11800	osa:4346529	4346529		
					Ma11_g23060	osa:4343164	4343164		
					Ma10_g15800	osa:4335789	4335789		
					Ma09_g31400	osa:4348690	4348690		
					Ma03_g32130	osa:4334101	4334101		

					Ma04_g39130<br>Ma06_g29240<br>Ma04_g03820<br>Ma11_g19510<br>Ma03_g03600<br>Ma06_g25950<br>Novel01373<br>Ma08_g30510<br>Ma04_g23230<br>Ma09_g12600<br>Ma03_g07770<br>Ma03_g14250<br>Ma10_g18010<br>Ma05_g14380<br>Ma08_g23180<br>Ma06_g12590<br>Ma08_g27070<br>Ma09_g31020<br>Ma06_g27120<br>Ma05_g06070<br>Ma11_g14310<br>Ma11_g08450<br>Ma10_g18940<br>Ma11_g03900<br>Ma07_g11010<br>Ma08_g20550<br>Ma03_g02590<br>Ma08_g15070<br>Ma11_g14380<br>Ma05_g18090<br>Ma09_g23530<br>Ma07_g13520<br>Ma04_g25530<br>Ma03_g10630<br>Ma01_g17210<br>Ma10_g12270<br>Ma10_g17250<br>Ma05_g15200<br>Ma09_g08470<br>Ma03_g16840<br>Ma03_g06970<br>Ma11_g13600<br>Ma06_g28940<br>Ma10_g09700<br>Ma03_g26340<br>Ma08_g10630<br>Ma06_g22100<br>Ma06_g26480<br>Ma10_g15370<br>Ma01_g17990<br>Ma03_g02700<br>Ma07_g11120<br>Ma10_g26330<br>Ma02_g20450<br>Ma02_g12240<br>Ma04_g40060<br>Ma03_g01370<br>Ma05_g11820<br>Ma01_g03420<br>Ma09_g24600<br>Ma04_g31640<br>Ma03_g08830<br>Ma10_g11950<br>Ma04_g26280<br>Ma05_g22240<br>Ma06_g16590<br>Ma03_g17510<br>Ma03_g09880<br>Ma02_g09950<br>Ma10_g29140<br>Ma02_g10050<br>Ma11_g02800<br>Ma10_g12590<br>Ma08_g04450<br>Ma03_g21800<br>Ma10_g18290	osa:4334171<br>osa:4332041<br>osa:4335430<br>osa:4330104<br>osa:4328168<br>osa:4334104<br>osa:4339846<br>osa:4344267<br>osa:4348834<br>osa:4330747<br>osa:4331824<br>osa:4346337<br>osa:4333888<br>osa:4332274<br>osa:4332788<br>osa:4352649<br>osa:4332134<br>osa:4344899<br>osa:4329593<br>osa:4339793<br>osa:4326794<br>osa:4324962<br>osa:4331779<br>osa:4330104<br>osa:4346981<br>osa:4343598<br>osa:4352615<br>osa:4348230<br>osa:4344268<br>osa:4344854<br>osa:4336928<br>osa:4327301<br>osa:4331130<br>osa:4351695<br>osa:4330737<br>osa:4350614<br>osa:4342517<br>osa:4349333<br>osa:4329593<br>osa:4340025<br>osa:4330843<br>osa:4332563<br>osa:4339718<br>osa:4334425<br>osa:4339675<br>osa:4337732<br>osa:4350524<br>osa:4334196<br>osa:4324599<br>osa:4343721<br>osa:4337818<br>osa:4332174<br>osa:4327178<br>osa:4349333<br>osa:4330164<br>osa:4325861<br>osa:4325687<br>osa:4349012<br>osa:4340008<br>osa:4347691<br>osa:4343366<br>osa:4344231<br>osa:4326465<br>osa:4346217<br>osa:4343249<br>osa:4324619<br>osa:4339892<br>osa:4332519<br>osa:4350053<br>osa:4339127<br>osa:4351610<br>osa:4336753<br>osa:4329518<br>osa:4348656<br>osa:4339554<br>osa:4352146	4334171<br>4332041<br>4335430<br>4330104<br>4328168<br>4334104<br>4339846<br>4344267<br>4348834<br>4330747<br>4331824<br>4346337<br>4333888<br>4332274<br>4332788<br>4352649<br>4332134<br>4344899<br>4329593<br>4339793<br>4326794<br>4324962<br>4331779<br>4330104<br>4346981<br>4343598<br>4352615<br>4348230<br>4344268<br>4344854<br>4336928<br>4327301<br>4331130<br>4351695<br>4330737<br>4350614<br>4342517<br>4349333<br>4329593<br>4340025<br>4330843<br>4332563<br>4339718<br>4334425<br>4339675<br>4337732<br>4350524<br>4334196<br>4324599<br>4343721<br>4337818<br>4332174<br>4327178<br>4349333<br>4330164<br>4325861<br>4325687<br>4349012<br>4340008<br>4347691<br>4343366<br>4344231<br>4326465<br>4346217<br>4343249<br>4324619<br>4339892<br>4332519<br>4350053<br>4339127<br>4351610<br>4336753<br>4329518<br>4348656<br>4339554<br>4352146		
Sphingolipid metabolism	12	22	0.105698179473	0.618522934881	Ma04_g18600<br>Ma06_g19820<br>Ma00_g01820<br>Ma04_g36700<br>Ma03_g01410<br>Ma00_g01810<br>Ma10_g12210<br>Ma11_g19510<br>Ma11_g03900<br>Ma05_g14380	osa:4331075<br>osa:4350171<br>osa:4330439<br>osa:4348876<br>osa:9268279<br>osa:4330439<br>osa:4350591<br>osa:4330104<br>osa:4330104<br>osa:4332274	4331075<br>4350171<br>4330439<br>4348876<br>9268279<br>4330439<br>4350591<br>4330104<br>4330104<br>4332274		

					Ma05_g09350	osa:4339457	4339457		
					Ma07_g26160	osa:4326680	4326680		
Carbon metabolism	86	225	0.108422165896	0.618522934881	Ma08_g07480	osa:4325027	4325027		
					Ma04_g04160	osa:4337230	4337230		
					Ma10_g28640	osa:4340677	4340677		
					Ma11_g08300	osa:4336216	4336216		
					Ma07_g11340	osa:4348582	4348582		
					Novel00240	osa:4337230	4337230		
					Ma02_g03830	osa:4338750	4338750		
					Ma10_g25720	osa:4345657	4345657		
					Ma11_g21050	osa:4330016	4330016		
					Ma10_g21340	osa:4338007	4338007		
					Ma06_g21100	osa:4337051	4337051		
					Ma06_g28550	osa:4345387	4345387		
					Ma11_g14450	osa:4352839	4352839		
					Ma08_g28170	osa:4324655	4324655		
					Ma03_g03600	osa:4328168	4328168		
					Ma08_g15550	osa:4351929	4351929		
					Ma09_g26210	osa:4352839	4352839		
					Ma08_g08880	osa:4346159	4346159		
					Ma05_g00310	osa:4341792	4341792		
					Ma04_g28920	osa:4342610	4342610		
					Ma09_g20570	osa:4337406	4337406		
					Ma07_g11510	osa:4331893	4331893		
					Ma05_g07850	osa:4326513	4326513		
					Ma06_g08130	osa:4352058	4352058		
					Ma08_g05120	osa:4342988	4342988		
					Ma08_g03130	osa:4333898	4333898		
					Ma07_g10560	osa:4327301	4327301		
					Ma03_g32130	osa:4334101	4334101		
					Ma03_g27870	osa:4347691	4347691		
					Ma05_g25700	osa:4327423	4327423		
					Ma09_g07280	osa:4329858	4329858		
					Ma04_g03820	osa:4335430	4335430		
					Ma08_g33830	osa:4328446	4328446		
					Ma02_g18360	osa:4327301	4327301		
					Ma05_g21880	osa:4335048	4335048		
					Ma09_g14520	osa:4347311	4347311		
					Ma03_g14310	osa:4346332	4346332		
					Ma01_g03570	osa:4340042	4340042		
					Ma11_g16750	osa:4341770	4341770		
					Ma10_g12550	osa:4336044	4336044		
					Ma04_g33980	osa:4330413	4330413		
					Ma08_g30510	osa:4344267	4344267		
					Ma05_g27700	osa:4336216	4336216		
					Ma04_g35390	osa:4349454	4349454		
					Ma02_g08140	osa:4350964	4350964		
					Ma09_g12600	osa:4330747	4330747		
					Ma06_g01900	osa:4341792	4341792		
					Ma06_g26610	osa:4331941	4331941		
					Ma05_g31060	osa:4325211	4325211		
					Ma03_g17510	osa:4339892	4339892		
					Ma06_g38960	osa:4336353	4336353		
					Ma09_g02110	osa:4336216	4336216		
					Ma06_g24830	osa:3131463	3131463		
					Novel00760	osa:4324442	4324442		
					Ma07_g22000	osa:4345962	4345962		
					Ma09_g07020	osa:4352058	4352058		
					Ma01_g19240	osa:4341792	4341792		
					Ma04_g01300	osa:4352871	4352871		
					Ma04_g27940	osa:4334101	4334101		
					Ma11_g07780	osa:4341713	4341713		
					Ma07_g13520	osa:4327301	4327301		
					Ma04_g25530	osa:4331130	4331130		
					Ma05_g05050	osa:4340042	4340042		
					Ma05_g22300	osa:4349897	4349897		
					Ma08_g16120	osa:4349897	4349897		
					Ma11_g20920	osa:4348176	4348176		
					Novel00441	osa:4341792	4341792		
					Ma10_g07330	osa:4341713	4341713		
					Ma11_g07790	osa:4337230	4337230		
					Ma01_g17610	osa:4337406	4337406		
					Ma02_g13970	osa:4328168	4328168		
					Ma05_g03450	osa:4330413	4330413		
					Ma10_g24940	osa:4347311	4347311		
					Ma03_g16830	osa:4340025	4340025		
					Ma11_g17540	osa:4336216	4336216		
					Ma09_g24600	osa:4347691	4347691		
					Ma07_g15870	osa:4328326	4328326		
					Ma07_g11500	osa:4331893	4331893		
					Ma07_g12740	osa:4349723	4349723		
					Ma03_g32890	osa:4326769	4326769		
					Ma08_g29310	osa:4329889	4329889		
					Ma02_g11180	osa:4339425	4339425		
					Ma02_g18420	osa:4341321	4341321		
					Ma10_g12590	osa:4329518	4329518		

					Ma04_g13550<br>Ma03_g16840	osa:4335430<br>osa:4340025	4335430<br>4340025		
Histidine metabolism	9	15	0.10962965231	0.618522934881	Ma10_g10490<br>Ma06_g27120<br>Ma06_g17860<br>Ma09_g08470<br>Ma09_g09840<br>Ma05_g05010<br>Novel01132<br>Ma03_g06120<br>Novel01419	osa:4329593<br>osa:4329593<br>osa:4324261<br>osa:4329593<br>osa:4324282<br>osa:4340725<br>osa:4324282<br>osa:4340725<br>osa:4324282	4329593<br>4329593<br>4324261<br>4329593<br>4324282<br>4340725<br>4324282<br>4340725<br>4324282		
Carbon fixation in photosynthetic organisms	33	77	0.110094955804	0.618522934881	Ma08_g07480<br>Ma05_g03450<br>Ma03_g16830<br>Ma11_g17540<br>Ma11_g08300<br>Ma06_g28550<br>Ma01_g19240<br>Ma03_g27870<br>Ma02_g03830<br>Ma05_g25700<br>Ma10_g25720<br>Ma04_g03820<br>Ma04_g01300<br>Ma09_g24600<br>Ma10_g21340<br>Novel00441<br>Ma03_g14310<br>Ma11_g16750<br>Ma05_g22300<br>Ma03_g32890<br>Ma08_g16120<br>Ma10_g12550<br>Ma04_g33980<br>Ma05_g27700<br>Ma03_g16840<br>Ma06_g01900<br>Ma08_g33830<br>Ma05_g31060<br>Ma05_g00310<br>Ma07_g15870<br>Ma09_g02110<br>Ma04_g13550<br>Ma06_g24830	osa:4325027<br>osa:4330413<br>osa:4340025<br>osa:4336216<br>osa:4336216<br>osa:4345387<br>osa:4341792<br>osa:4347691<br>osa:4338750<br>osa:4327423<br>osa:4345657<br>osa:4335430<br>osa:4352871<br>osa:4347691<br>osa:4338007<br>osa:4341792<br>osa:4346332<br>osa:4341770<br>osa:4349897<br>osa:4326769<br>osa:4349897<br>osa:4336044<br>osa:4330413<br>osa:4336216<br>osa:4340025<br>osa:4341792<br>osa:4328446<br>osa:4325211<br>osa:4341792<br>osa:4328326<br>osa:4336216<br>osa:4335430<br>osa:3131463	4325027<br>4330413<br>4340025<br>4336216<br>4336216<br>4345387<br>4341792<br>4347691<br>4338750<br>4327423<br>4345657<br>4335430<br>4352871<br>4347691<br>4338007<br>4341792<br>4346332<br>4341770<br>4349897<br>4326769<br>4349897<br>4336044<br>4330413<br>4336216<br>4340025<br>4341792<br>4328446<br>4325211<br>4341792<br>4328326<br>4336216<br>4335430<br>3131463		
Sulfur metabolism	17	35	0.114348777877	0.618522934881	Ma08_g28170<br>Ma03_g32130<br>Ma04_g10990<br>Ma02_g18420<br>Ma06_g26610<br>Ma09_g26210<br>Ma08_g27570<br>Ma04_g39880<br>Ma11_g24380<br>Ma01_g16340<br>Ma05_g21880<br>Ma04_g27940<br>Ma06_g19810<br>Ma11_g14450<br>Ma01_g21430<br>Ma02_g11180<br>Ma01_g07600	osa:4324655<br>osa:4334101<br>osa:4343681<br>osa:4341321<br>osa:4331941<br>osa:4352839<br>osa:4334073<br>osa:4343348<br>osa:4331065<br>osa:4339248<br>osa:4335048<br>osa:4334101<br>osa:4338825<br>osa:4352839<br>osa:4326319<br>osa:4339425<br>osa:4334073	4324655<br>4334101<br>4343681<br>4341321<br>4331941<br>4352839<br>4334073<br>4343348<br>4331065<br>4339248<br>4335048<br>4334101<br>4338825<br>4352839<br>4326319<br>4339425<br>4334073		
Pentose phosphate pathway	21	48	0.156688558194	0.792845497268	Ma07_g13520<br>Ma08_g07480<br>Ma08_g05120<br>Ma02_g13970<br>Ma11_g16750<br>Ma05_g22300<br>Ma03_g16830<br>Ma04_g03820<br>Ma10_g28640<br>Ma08_g16120<br>Ma03_g17510<br>Ma07_g11340<br>Ma08_g15070<br>Ma02_g18360<br>Ma08_g29310<br>Ma10_g24940<br>Ma04_g13550<br>Ma07_g10560<br>Ma03_g03600<br>Ma09_g14520<br>Ma03_g16840	osa:4327301<br>osa:4325027<br>osa:4342988<br>osa:4328168<br>osa:4341770<br>osa:4349897<br>osa:4340025<br>osa:4335430<br>osa:4340677<br>osa:4349897<br>osa:4339892<br>osa:4348582<br>osa:4348230<br>osa:4327301<br>osa:4329889<br>osa:4347311<br>osa:4335430<br>osa:4327301<br>osa:4328168<br>osa:4347311<br>osa:4340025	4327301<br>4325027<br>4342988<br>4328168<br>4341770<br>4349897<br>4340025<br>4335430<br>4340677<br>4349897<br>4339892<br>4348582<br>4348230<br>4327301<br>4329889<br>4347311<br>4335430<br>4327301<br>4328168<br>4347311<br>4340025		
Nitrogen metabolism	13	27	0.159901612894	0.792845497268	Ma02_g01040<br>Ma02_g22240<br>Ma02_g08780	osa:4345604<br>osa:4329556<br>osa:4329556	4345604<br>4329556<br>4329556		

					Ma03_g32240<br>Ma09_g14720<br>Ma02_g12240<br>Novel00953<br>Ma03_g06990<br>Ma05_g05300<br>Ma06_g29120<br>Ma03_g21060<br>Ma00_g04030<br>Ma08_g28590	osa:4326583<br>osa:4326014<br>osa:4330164<br>osa:4345604<br>osa:4345604<br>osa:4347305<br>osa:4326583<br>osa:4330649<br>osa:4330649<br>osa:4324398	4326583<br>4326014<br>4330164<br>4345604<br>4345604<br>4347305<br>4326583<br>4330649<br>4330649<br>4324398		
Glycine, serine and threonine metabolism	23	55	0.184451104833	0.877987259004	Novel01203<br>Ma06_g08130<br>Ma07_g22000<br>Ma10_g18940<br>Ma09_g07020<br>Ma01_g01610<br>Novel00240<br>Ma04_g04160<br>Ma06_g21100<br>Ma11_g07780<br>Ma07_g12740<br>Ma09_g12600<br>Ma05_g21330<br>Ma02_g08140<br>Ma10_g07330<br>Ma06_g12620<br>Ma09_g15520<br>Ma10_g09700<br>Ma08_g03130<br>Ma00_g05010<br>Ma05_g01700<br>Ma10_g16940<br>Ma11_g07790	osa:4334425<br>osa:4352058<br>osa:4345962<br>osa:4331779<br>osa:4352058<br>osa:4349114<br>osa:4337230<br>osa:4337230<br>osa:4337051<br>osa:4341713<br>osa:4349723<br>osa:4330747<br>osa:4327981<br>osa:4350964<br>osa:4341713<br>osa:4334188<br>osa:4345606<br>osa:4334425<br>osa:4333898<br>osa:4335335<br>osa:4346642<br>osa:4336153<br>osa:4337230	4334425<br>4352058<br>4345962<br>4331779<br>4352058<br>4349114<br>4337230<br>4337230<br>4337051<br>4341713<br>4349723<br>4330747<br>4327981<br>4350964<br>4341713<br>4334188<br>4345606<br>4334425<br>4333898<br>4335335<br>4346642<br>4336153<br>4337230		
Fructose and mannose metabolism	22	53	0.19854317177	0.90871682464	Ma08_g07480<br>Ma11_g08450<br>Ma07_g11340<br>Ma07_g10560<br>Ma02_g04620<br>Ma02_g21000<br>Ma03_g27870<br>Ma04_g26260<br>Ma05_g22300<br>Ma10_g18160<br>Ma09_g24600<br>Ma02_g18360<br>Ma03_g08830<br>Ma04_g26280<br>Ma07_g13520<br>Ma11_g16750<br>Ma10_g16460<br>Ma08_g16120<br>Ma05_g05600<br>Ma05_g31060<br>Ma04_g16060<br>Ma09_g24170	osa:4325027<br>osa:4324962<br>osa:4348582<br>osa:4327301<br>osa:4340050<br>osa:4332373<br>osa:4347691<br>osa:4346217<br>osa:4349897<br>osa:4332014<br>osa:4347691<br>osa:4327301<br>osa:4344231<br>osa:4346217<br>osa:4327301<br>osa:4341770<br>osa:4324962<br>osa:4349897<br>osa:4340579<br>osa:4325211<br>osa:4324962<br>osa:4337437	4325027<br>4324962<br>4348582<br>4327301<br>4340050<br>4332373<br>4347691<br>4346217<br>4349897<br>4332014<br>4347691<br>4327301<br>4344231<br>4346217<br>4327301<br>4341770<br>4324962<br>4349897<br>4340579<br>4325211<br>4324962<br>4337437		
Propanoate metabolism	7	14	0.23901912012	0.999999998471	Ma08_g15550<br>Ma07_g06750<br>Ma11_g21050<br>Ma04_g28920<br>Ma10_g12590<br>Ma08_g30510<br>Ma07_g06760	osa:4351929<br>osa:4328012<br>osa:4330016<br>osa:4342610<br>osa:4329518<br>osa:4344267<br>osa:4328012	4351929<br>4328012<br>4330016<br>4342610<br>4329518<br>4344267<br>4328012		
Peroxisome	30	78	0.244710891779	0.999999998471	Ma04_g10090<br>Ma06_g21190<br>Ma02_g04310<br>Ma07_g22000<br>Ma02_g24010<br>Ma07_g03790<br>Ma07_g06830<br>Ma03_g00220<br>Ma03_g14690<br>Ma09_g30420<br>Ma09_g20440<br>Ma03_g00940<br>Ma10_g14370<br>Ma11_g15660<br>Ma02_g10050<br>Ma02_g03420<br>Ma09_g30400<br>Ma10_g07820<br>Ma03_g19080<br>Novel01373<br>Ma04_g08370<br>Ma03_g01840<br>Ma03_g13720	osa:4326266<br>osa:4337048<br>osa:4332846<br>osa:4345962<br>osa:4340986<br>osa:4332846<br>osa:4346360<br>osa:4331150<br>osa:4328073<br>osa:4346301<br>osa:4346329<br>osa:4346459<br>osa:4328861<br>osa:4348804<br>osa:4351610<br>osa:4336980<br>osa:4346301<br>osa:4346329<br>osa:4338417<br>osa:4339846<br>osa:4324427<br>osa:4343908<br>osa:4331150	4326266<br>4337048<br>4332846<br>4345962<br>4340986<br>4332846<br>4346360<br>4331150<br>4328073<br>4346301<br>4346329<br>4346459<br>4328861<br>4348804<br>4351610<br>4336980<br>4346301<br>4346329<br>4338417<br>4339846<br>4324427<br>4343908<br>4331150		

					Ma11_g16120<br>Ma09_g11860<br>Ma08_g26760<br>Ma04_g14180<br>Ma06_g38960<br>Ma09_g30410<br>Ma04_g06850	osa:4330220<br>osa:4347528<br>osa:4328779<br>osa:4352306<br>osa:4336353<br>osa:4346301<br>osa:4332082	4330220<br>4347528<br>4328779<br>4352306<br>4336353<br>4346301<br>4332082		
Cyanoamino acid metabolism	15	36	0.251976195133	0.999999998471	Ma06_g16830<br>Ma05_g23890<br>Ma11_g05360<br>Ma06_g08130<br>Ma06_g29240<br>Ma07_g22060<br>Ma08_g13290<br>Ma09_g07020<br>Ma05_g21880<br>Ma06_g29250<br>Ma03_g32060<br>Ma08_g25110<br>Ma06_g16590<br>Ma03_g00730<br>Ma09_g06900	osa:4324526<br>osa:4338560<br>osa:4338560<br>osa:4352058<br>osa:4332041<br>osa:4336146<br>osa:4330077<br>osa:4352058<br>osa:4335048<br>osa:4332041<br>osa:4334116<br>osa:4336146<br>osa:4324619<br>osa:9266710<br>osa:4332041	4324526<br>4338560<br>4338560<br>4352058<br>4332041<br>4336146<br>4330077<br>4352058<br>4335048<br>4332041<br>4334116<br>4336146<br>4324619<br>9266710<br>4332041		
Phenylpropanoid biosynthesis	46	125	0.257265480493	0.999999998471	Ma06_g16060<br>Ma01_g21440<br>Ma06_g19750<br>Ma03_g06130<br>Ma06_g16100<br>Ma07_g22060<br>Ma01_g21520<br>Ma05_g31950<br>Ma07_g11120<br>Ma02_g07020<br>Ma03_g05380<br>Ma08_g25110<br>Ma06_g16590<br>Ma02_g04920<br>Ma09_g06900<br>Ma11_g05360<br>Ma04_g10150<br>Ma06_g29240<br>Ma10_g25840<br>Ma06_g10250<br>Ma10_g15940<br>Ma09_g13580<br>Ma01_g04420<br>Ma09_g31400<br>Ma09_g23330<br>Ma10_g27820<br>Ma03_g32060<br>Ma02_g22450<br>Ma03_g03400<br>Ma11_g19280<br>Ma04_g05290<br>Ma05_g27310<br>Ma11_g21180<br>Ma06_g10290<br>Ma09_g30120<br>Ma07_g09220<br>Ma05_g23890<br>Ma09_g25700<br>Ma05_g27130<br>Ma03_g13910<br>Ma01_g03820<br>Ma06_g29250<br>Ma08_g10630<br>Ma03_g05400<br>Ma11_g15990<br>Ma10_g23920	osa:4341249<br>osa:4347520<br>osa:4344496<br>osa:4340240<br>osa:4341249<br>osa:4336146<br>osa:4344496<br>osa:4351300<br>osa:4332174<br>osa:4347074<br>osa:4329324<br>osa:4336146<br>osa:4324619<br>osa:4341861<br>osa:4332041<br>osa:4338560<br>osa:4345689<br>osa:4332041<br>osa:4332174<br>osa:4338409<br>osa:4324554<br>osa:4329329<br>osa:4336415<br>osa:4348690<br>osa:4328485<br>osa:4341247<br>osa:4334116<br>osa:4324557<br>osa:4341249<br>osa:4344045<br>osa:4351300<br>osa:4347520<br>osa:4336415<br>osa:4351300<br>osa:4337482<br>osa:4341249<br>osa:4338560<br>osa:4340240<br>osa:4328684<br>osa:4346726<br>osa:4343453<br>osa:4332041<br>osa:4337732<br>osa:4329329<br>osa:4349097<br>osa:4345689	4341249<br>4347520<br>4344496<br>4340240<br>4341249<br>4336146<br>4344496<br>4351300<br>4332174<br>4347074<br>4329324<br>4336146<br>4324619<br>4341861<br>4332041<br>4338560<br>4345689<br>4332041<br>4332174<br>4338409<br>4324554<br>4329329<br>4336415<br>4348690<br>4328485<br>4341247<br>4334116<br>4324557<br>4341249<br>4344045<br>4351300<br>4347520<br>4336415<br>4351300<br>4337482<br>4341249<br>4338560<br>4340240<br>4328684<br>4346726<br>4343453<br>4332041<br>4337732<br>4329329<br>4349097<br>4345689		
Endocytosis	38	103	0.277408890598	0.999999998471	Ma09_g07430<br>Ma06_g13850<br>Ma05_g03640<br>Ma01_g05510<br>Ma02_g18000<br>Ma01_g08380<br>Ma05_g05940<br>Ma05_g02240<br>Ma06_g33480<br>Ma04_g02360<br>Ma07_g18790<br>Ma07_g16530<br>Ma10_g29250<br>Ma09_g28540<br>Ma04_g11640<br>Ma08_g21660<br>Ma09_g03950<br>Ma10_g26890	osa:4351875<br>osa:4351875<br>osa:4348891<br>osa:4339623<br>osa:4351208<br>osa:4347745<br>osa:4344016<br>osa:4333538<br>osa:4341266<br>osa:4327591<br>osa:4347745<br>osa:4352862<br>osa:4327591<br>osa:4330565<br>osa:4331873<br>osa:4351208<br>osa:4351208<br>osa:4327647	4351875<br>4351875<br>4348891<br>4339623<br>4351208<br>4347745<br>4344016<br>4333538<br>4341266<br>4327591<br>4347745<br>4352862<br>4327591<br>4330565<br>4331873<br>4351208<br>4351208<br>4327647		

					Ma08_g34730<br>Ma05_g08750<br>Ma01_g04080<br>Ma03_g19140<br>Ma03_g12870<br>Ma06_g30090<br>Ma09_g23230<br>Ma02_g11360<br>Ma06_g17930<br>Ma07_g15130<br>Ma06_g37750<br>Ma01_g08090<br>Ma04_g02580<br>Ma05_g25590<br>Ma06_g06700<br>Ma04_g06320<br>Ma08_g21650<br>Ma09_g05400<br>Ma09_g18500<br>Ma10_g06890	osa:4339012<br>osa:4327852<br>osa:4343612<br>osa:4327591<br>osa:4341638<br>osa:4351875<br>osa:4341494<br>osa:4341266<br>osa:4337536<br>osa:4332427<br>osa:4327647<br>osa:4327647<br>osa:4341266<br>osa:4341894<br>osa:4342791<br>osa:4341494<br>osa:4351208<br>osa:4327180<br>osa:4340850<br>osa:4327180	4339012<br>4327852<br>4343612<br>4327591<br>4341638<br>4351875<br>4341494<br>4341266<br>4337536<br>4332427<br>4327647<br>4327647<br>4341266<br>4341894<br>4342791<br>4341494<br>4351208<br>4327180<br>4340850<br>4327180		
Linoleic acid metabolism	7	15	0.281521390223	0.999999998471	Ma08_g23400<br>Ma03_g07770<br>Ma03_g11520<br>Ma06_g26870<br>Ma09_g15420<br>Ma07_g11670<br>Ma09_g12090	osa:4331824<br>osa:4331824<br>osa:4328603<br>osa:4333821<br>osa:4331824<br>osa:4333862<br>osa:4345994	4331824<br>4331824<br>4328603<br>4333821<br>4331824<br>4333862<br>4345994		
Steroid biosynthesis	11	26	0.284251847108	0.999999998471	Ma07_g28990<br>Ma09_g03480<br>Ma10_g12270<br>Ma08_g04900<br>Ma04_g32060<br>Ma10_g26480<br>Ma08_g04920<br>Ma06_g11320<br>Ma11_g20450<br>Ma03_g01370<br>Ma10_g14440	osa:4342690<br>osa:4342171<br>osa:4350614<br>osa:4332153<br>osa:4331540<br>osa:4351226<br>osa:4332153<br>osa:4325687<br>osa:4349278<br>osa:4325687<br>osa:4325563	4342690<br>4342171<br>4350614<br>4332153<br>4331540<br>4351226<br>4332153<br>4325687<br>4349278<br>4325687<br>4325563		
Stilbenoid, diarylheptanoid and gingerol biosynthesis	8	18	0.2937445041	0.999999998471	Ma03_g05400<br>Ma09_g25700<br>Ma07_g09040<br>Ma07_g06700<br>Ma03_g06130<br>Ma06_g10250<br>Ma03_g05380<br>Ma09_g13580	osa:4329329<br>osa:4340240<br>osa:4341325<br>osa:4349111<br>osa:4340240<br>osa:4338409<br>osa:4329324<br>osa:4329329	4329329<br>4340240<br>4341325<br>4349111<br>4340240<br>4338409<br>4329324<br>4329329		
Ribosome	114	330	0.296883553899	0.999999998471	Ma11_g15870<br>Ma05_g14780<br>Ma05_g19200<br>Ma10_g13230<br>Ma09_g07060<br>Ma04_g29740<br>Ma04_g28630<br>Ma11_g20970<br>Novel00326<br>Ma01_g21100<br>Ma07_g21220<br>Ma04_g11020<br>Ma05_g16500<br>Ma08_g29860<br>Ma02_g21120<br>Ma02_g08870<br>Ma06_g17050<br>Ma11_g23780<br>Ma06_g17110<br>Ma11_g13860<br>Ma09_g00010<br>Ma09_g25400<br>Ma04_g17690<br>Ma05_g24650<br>Ma01_g10140<br>Ma03_g28710<br>Ma01_g15830<br>Ma04_g29360<br>Ma03_g22300<br>Ma10_g06200<br>Ma08_g17750<br>Ma10_g07390<br>Novel01247<br>Ma08_g33500<br>Ma10_g18960<br>Ma11_g24100<br>Ma04_g06070<br>Ma04_g24620<br>Ma00_g02380	osa:4331459<br>osa:4348607<br>osa:4334131<br>osa:4332545<br>osa:4335547<br>osa:9272292<br>osa:4326469<br>osa:4352560<br>osa:4351513<br>osa:4327257<br>osa:4346086<br>osa:4331942<br>osa:4343909<br>osa:4348846<br>osa:4344590<br>osa:4350869<br>osa:4332466<br>osa:4332802<br>osa:4339104<br>osa:4350516<br>osa:4325555<br>osa:9266960<br>osa:4334580<br>osa:4334438<br>osa:4327004<br>osa:4339923<br>osa:4332089<br>osa:9266562<br>osa:4351374<br>osa:4335607<br>osa:4351513<br>osa:4336914<br>osa:4348843<br>osa:4341869<br>osa:4328635<br>osa:4342408<br>osa:4348846<br>osa:4325273<br>osa:4327483	4331459<br>4348607<br>4334131<br>4332545<br>4335547<br>9272292<br>4326469<br>4352560<br>4351513<br>4327257<br>4346086<br>4331942<br>4343909<br>4348846<br>4344590<br>4350869<br>4332466<br>4332802<br>4339104<br>4350516<br>4325555<br>9266960<br>4334580<br>4334438<br>4327004<br>4339923<br>4332089<br>9266562<br>4351374<br>4335607<br>4351513<br>4336914<br>4348843<br>4341869<br>4328635<br>4342408<br>4348846<br>4325273<br>4327483		

					Ma05_g02510<br>Ma03_g01330<br>Ma03_g03800<br>Ma09_g22950<br>Ma10_g00610<br>Ma06_g24940<br>Ma00_g03400<br>Ma02_g23940<br>Ma10_g26340<br>Ma02_g03590<br>Ma06_g26930<br>Ma01_g00800<br>Ma05_g21010<br>Ma02_g17430<br>Ma04_g10730<br>Ma11_g23830<br>Ma11_g16510<br>Ma05_g28030<br>Novel00874<br>Ma01_g20160<br>Ma11_g14090<br>Ma08_g02490<br>Ma07_g12470<br>Ma10_g29570<br>Ma02_g10840<br>Ma08_g31060<br>Ma06_g32850<br>Ma11_g18070<br>Ma02_g03280<br>Ma09_g25820<br>Ma04_g08190<br>Ma06_g05060<br>Ma10_g00920<br>Ma08_g34120<br>Ma09_g23430<br>Ma05_g08080<br>Ma01_g15300<br>Ma03_g01510<br>Ma06_g24450<br>Ma10_g11380<br>Ma09_g21730<br>Ma04_g27000<br>Ma01_g13930<br>Ma03_g01060<br>Ma06_g07140<br>Ma02_g16430<br>Ma06_g10220<br>Ma06_g35440<br>Ma04_g15270<br>Ma04_g10490<br>Ma10_g17150<br>Ma04_g12660<br>Ma03_g19680<br>Ma00_g02740<br>Ma05_g06910<br>Ma03_g13020<br>Ma09_g20220<br>Ma08_g24590<br>Ma03_g21390<br>Ma06_g11370<br>Ma05_g22670<br>Ma01_g17660<br>Ma10_g29730<br>Ma08_g21210<br>Ma06_g28790<br>Ma05_g18200<br>Ma03_g00680<br>Novel00357<br>Ma04_g36730<br>Ma06_g24090<br>Ma04_g24910<br>Ma07_g26990<br>Ma10_g22710<br>Ma11_g02570<br>Ma01_g08150	osa:9266562<br>osa:4351513<br>osa:4337653<br>osa:4348846<br>osa:4328797<br>osa:4330946<br>osa:4351604<br>osa:4351374<br>osa:9267955<br>osa:4338571<br>osa:4349726<br>osa:4341664<br>osa:4346540<br>osa:4328797<br>osa:4329171<br>osa:4328016<br>osa:4334651<br>osa:4350406<br>osa:4352797<br>osa:4343869<br>osa:4329362<br>osa:4335607<br>osa:4330763<br>osa:4343869<br>osa:4339648<br>osa:4328472<br>osa:9266562<br>osa:4329362<br>osa:4342408<br>osa:4348607<br>osa:4349409<br>osa:4340887<br>osa:4343869<br>osa:4332802<br>osa:4336914<br>osa:4329361<br>osa:4333083<br>osa:4351374<br>osa:4334154<br>osa:4338938<br>osa:4336914<br>osa:4336317<br>osa:4341262<br>osa:4334580<br>osa:4328797<br>osa:6450193<br>osa:4337928<br>osa:4327674<br>osa:4349409<br>osa:4349409<br>osa:4346086<br>osa:4336914<br>osa:4352308<br>osa:4332773<br>osa:4327391<br>osa:4331014<br>osa:4339552<br>osa:4330425<br>osa:4324446<br>osa:4351513<br>osa:4331459<br>osa:4332773<br>osa:4345669<br>osa:4328932<br>osa:4326865<br>osa:4333233<br>osa:4328016<br>osa:4330946<br>osa:4348839<br>osa:4328826<br>osa:4327674<br>osa:4338938<br>osa:4327391<br>osa:4331346<br>osa:4342771	9266562<br>4351513<br>4337653<br>4348846<br>4328797<br>4330946<br>4351604<br>4351374<br>9267955<br>4338571<br>4349726<br>4341664<br>4346540<br>4328797<br>4329171<br>4328016<br>4334651<br>4350406<br>4352797<br>4343869<br>4329362<br>4335607<br>4330763<br>4343869<br>4339648<br>4328472<br>9266562<br>4329362<br>4342408<br>4348607<br>4349409<br>4340887<br>4343869<br>4332802<br>4336914<br>4329361<br>4333083<br>4351374<br>4334154<br>4338938<br>4336914<br>4336317<br>4341262<br>4334580<br>4328797<br>6450193<br>4337928<br>4327674<br>4349409<br>4349409<br>4346086<br>4336914<br>4352308<br>4332773<br>4327391<br>4331014<br>4339552<br>4330425<br>4324446<br>4351513<br>4331459<br>4332773<br>4345669<br>4328932<br>4326865<br>4333233<br>4328016<br>4330946<br>4348839<br>4328826<br>4327674<br>4338938<br>4327391<br>4331346<br>4342771		
Fatty acid degradation	16	41	0.311289265065	0.999999998471	Ma04_g25530<br>Ma08_g29910<br>Ma07_g11500<br>Ma03_g00220<br>Ma02_g24010<br>Ma08_g30510<br>Ma09_g08110<br>Ma03_g06120<br>Ma05_g05010<br>Novel01373<br>Ma11_g15660	osa:4331130<br>osa:4350053<br>osa:4331893<br>osa:4331150<br>osa:4340986<br>osa:4344267<br>osa:4350053<br>osa:4340725<br>osa:4340725<br>osa:4339846<br>osa:4348804	4331130<br>4350053<br>4331893<br>4331150<br>4340986<br>4344267<br>4350053<br>4340725<br>4340725<br>4339846<br>4348804		

					Ma02_g09950<br>Ma04_g31940<br>Ma02_g10050<br>Ma07_g11510<br>Ma03_g13720	osa:4350053<br>osa:4330090<br>osa:4351610<br>osa:4331893<br>osa:4331150	4350053<br>4330090<br>4351610<br>4331893<br>4331150		
Ascorbate and aldarate metabolism	14	36	0.332436313342	0.999999998471	Ma06_g14310<br>Ma07_g28480<br>Ma05_g24340<br>Ma07_g28490<br>Ma04_g08270<br>Ma03_g31530<br>Ma03_g06120<br>Ma07_g28080<br>Ma07_g28090<br>Ma05_g05010<br>Ma06_g08980<br>Ma07_g07290<br>Ma10_g18290<br>Ma06_g15020	osa:4351698<br>osa:4352223<br>osa:4341967<br>osa:4352223<br>osa:4341305<br>osa:4352146<br>osa:4340725<br>osa:4333156<br>osa:4333156<br>osa:4340725<br>osa:4351698<br>osa:4336886<br>osa:4352146<br>osa:4351698	4351698<br>4352223<br>4341967<br>4352223<br>4341305<br>4352146<br>4340725<br>4333156<br>4333156<br>4340725<br>4351698<br>4336886<br>4352146<br>4351698		
Phenylalanine, tyrosine and tryptophan biosynthesis	16	42	0.339162436572	0.999999998471	Ma08_g19500<br>Ma09_g07380<br>Ma03_g20800<br>Ma01_g14880<br>Ma06_g07590<br>Ma05_g20810<br>Ma10_g09700<br>Ma10_g08510<br>Ma02_g05120<br>Ma10_g11420<br>Novel01203<br>Ma03_g11800<br>Ma09_g29570<br>Ma06_g21530<br>Ma08_g33410<br>Ma08_g14830	osa:4335756<br>osa:4332341<br>osa:4340547<br>osa:4343946<br>osa:4333918<br>osa:4332477<br>osa:4334425<br>osa:4347717<br>osa:4340026<br>osa:4335756<br>osa:4334425<br>osa:4346529<br>osa:4329101<br>osa:4325444<br>osa:4335756<br>osa:4332286	4335756<br>4332341<br>4340547<br>4343946<br>4333918<br>4332477<br>4334425<br>4347717<br>4340026<br>4335756<br>4334425<br>4346529<br>4329101<br>4325444<br>4335756<br>4332286		
Arginine and proline metabolism	23	63	0.353380096863	0.999999998471	Ma06_g26480<br>Ma08_g02290<br>Ma05_g23060<br>Ma07_g20920<br>Ma02_g12240<br>Ma11_g23060<br>Ma06_g05530<br>Ma05_g11820<br>Ma01_g03420<br>Ma05_g15910<br>Ma03_g06120<br>Ma00_g04600<br>Ma03_g04640<br>Ma00_g04030<br>Ma05_g26570<br>Ma09_g13000<br>Ma02_g05150<br>Ma05_g00590<br>Ma05_g05010<br>Ma09_g24200<br>Ma04_g06200<br>Ma04_g18620<br>Ma03_g21060	osa:4334196<br>osa:4338979<br>osa:4349318<br>osa:4329955<br>osa:4330164<br>osa:4343164<br>osa:4329955<br>osa:4349012<br>osa:4340008<br>osa:4333177<br>osa:4340725<br>osa:4337360<br>osa:4329955<br>osa:4330649<br>osa:4329955<br>osa:4347103<br>osa:4340008<br>osa:4337046<br>osa:4340725<br>osa:4349012<br>osa:4342996<br>osa:4334912<br>osa:4330649	4334196<br>4338979<br>4349318<br>4329955<br>4330164<br>4343164<br>4329955<br>4349012<br>4340008<br>4333177<br>4340725<br>4337360<br>4329955<br>4330649<br>4329955<br>4347103<br>4340008<br>4337046<br>4340725<br>4349012<br>4342996<br>4334912<br>4330649		
Other glycan degradation	6	14	0.362929600983	0.999999998471	Ma06_g19820<br>Ma04_g36700<br>Ma05_g17580<br>Ma06_g01700<br>Ma05_g09350<br>Ma06_g01670	osa:4350171<br>osa:4348876<br>osa:4348080<br>osa:4324914<br>osa:4339457<br>osa:4338802	4350171<br>4348876<br>4348080<br>4324914<br>4339457<br>4338802		
Glycerolipid metabolism	16	43	0.36755053441	0.999999998471	Ma05_g05010<br>Ma09_g27580<br>Ma03_g02590<br>Ma08_g02250<br>Ma05_g29560<br>Ma08_g02270<br>Ma07_g10180<br>Ma03_g25580<br>Ma03_g06120<br>Ma10_g10450<br>Ma05_g12640<br>Ma09_g28900<br>Ma10_g21240<br>Ma04_g26360<br>Ma09_g08810<br>Ma09_g03520	osa:4340725<br>osa:4345212<br>osa:4352615<br>osa:4332372<br>osa:4351119<br>osa:4332054<br>osa:4350917<br>osa:4351119<br>osa:4340725<br>osa:4349835<br>osa:4349006<br>osa:4338045<br>osa:4338045<br>osa:4330302<br>osa:4345212<br>osa:4332372	4340725<br>4345212<br>4352615<br>4332372<br>4351119<br>4332054<br>4350917<br>4351119<br>4340725<br>4349835<br>4349006<br>4338045<br>4338045<br>4330302<br>4345212<br>4332372		
Cutin, suberine and wax biosynthesis	7	17	0.37072341628	0.999999998471	Ma11_g15810<br>Ma09_g12850<br>Ma02_g13310<br>Ma11_g19740<br>Ma09_g30420	osa:4330012<br>osa:4350569<br>osa:4348947<br>osa:4330012<br>osa:4346301	4330012<br>4350569<br>4348947<br>4330012<br>4346301		

					Ma09_g30410<br>Ma09_g30400	osa:4346301<br>osa:4346301	4346301<br>4346301		
Glycosphingolipid biosynthesis - ganglio series	3	6	0.385526948481	0.999999998471	Ma05_g09350<br>Ma06_g01700<br>Ma06_g01670	osa:4339457<br>osa:4324914<br>osa:4338802	4339457<br>4324914<br>4338802		
Ubiquinone and other terpenoid-quinone biosynthesis	12	32	0.390088561412	0.999999998471	Ma02_g14500<br>Ma04_g07830<br>Ma09_g23330<br>Ma03_g05400<br>Ma04_g12630<br>Ma03_g29220<br>Ma08_g21280<br>Ma06_g10250<br>Ma03_g05380<br>Ma09_g29570<br>Ma09_g13580<br>Ma04_g07860	osa:4352791<br>osa:4327859<br>osa:4328485<br>osa:4329329<br>osa:4341663<br>osa:4341663<br>osa:4329007<br>osa:4338409<br>osa:4329324<br>osa:4329101<br>osa:4329329<br>osa:4327859	4352791<br>4327859<br>4328485<br>4329329<br>4341663<br>4341663<br>4329007<br>4338409<br>4329324<br>4329101<br>4329329<br>4327859		
ABC transporters	4	9	0.399232758074	0.999999998471	Ma01_g17310<br>Ma04_g04450<br>Ma04_g26720<br>Ma08_g30300	osa:9268765<br>osa:4346344<br>osa:4328568<br>osa:9268765	9268765<br>4346344<br>4328568<br>9268765		
Brassinosteroid biosynthesis	4	9	0.399232758074	0.999999998471	Ma04_g11650<br>Ma08_g27070<br>Ma05_g04020<br>Ma10_g27460	osa:4324809<br>osa:4332134<br>osa:4336116<br>osa:4327329	4324809<br>4332134<br>4336116<br>4327329		
Phenylalanine metabolism	34	99	0.42090950187	0.999999998471	Ma06_g16060<br>Ma01_g21440<br>Ma06_g19750<br>Ma03_g13910<br>Ma06_g16100<br>Ma01_g21520<br>Ma05_g31950<br>Ma07_g11120<br>Ma03_g05380<br>Ma09_g29570<br>Ma02_g04920<br>Ma11_g19280<br>Ma10_g25840<br>Ma06_g10250<br>Ma10_g15940<br>Ma09_g13580<br>Ma01_g04420<br>Ma09_g23330<br>Ma10_g27820<br>Ma02_g22450<br>Ma03_g03400<br>Ma04_g05290<br>Ma05_g27310<br>Ma11_g21180<br>Ma06_g10290<br>Ma09_g30120<br>Ma07_g09220<br>Ma09_g25700<br>Ma03_g06130<br>Ma00_g05010<br>Ma01_g03820<br>Ma10_g16940<br>Ma08_g10630<br>Ma03_g05400	osa:4341249<br>osa:4347520<br>osa:4344496<br>osa:4346726<br>osa:4341249<br>osa:4344496<br>osa:4351300<br>osa:4332174<br>osa:4329324<br>osa:4329101<br>osa:4341861<br>osa:4344045<br>osa:4332174<br>osa:4338409<br>osa:4324554<br>osa:4329329<br>osa:4336415<br>osa:4328485<br>osa:4341247<br>osa:4324557<br>osa:4341249<br>osa:4351300<br>osa:4347520<br>osa:4336415<br>osa:4351300<br>osa:4337482<br>osa:4341249<br>osa:4340240<br>osa:4340240<br>osa:4335335<br>osa:4343453<br>osa:4336153<br>osa:4337732<br>osa:4329329	4341249<br>4347520<br>4344496<br>4346726<br>4341249<br>4344496<br>4351300<br>4332174<br>4329324<br>4329101<br>4341861<br>4344045<br>4332174<br>4338409<br>4324554<br>4329329<br>4336415<br>4328485<br>4341247<br>4324557<br>4341249<br>4351300<br>4347520<br>4336415<br>4351300<br>4337482<br>4341249<br>4340240<br>4340240<br>4335335<br>4343453<br>4336153<br>4337732<br>4329329		
beta-Alanine metabolism	12	33	0.42381584305	0.999999998471	Ma05_g22240<br>Ma08_g15550<br>Ma05_g00590<br>Ma04_g28920<br>Ma00_g05010<br>Ma05_g05010<br>Ma00_g04600<br>Ma08_g30510<br>Ma10_g16940<br>Ma04_g06200<br>Ma04_g37810<br>Ma03_g06120	osa:4343249<br>osa:4351929<br>osa:4337046<br>osa:4342610<br>osa:4335335<br>osa:4340725<br>osa:4337360<br>osa:4344267<br>osa:4336153<br>osa:4342996<br>osa:4333932<br>osa:4340725	4343249<br>4351929<br>4337046<br>4342610<br>4335335<br>4340725<br>4337360<br>4344267<br>4336153<br>4342996<br>4333932<br>4340725		
Biosynthesis of unsaturated fatty acids	14	40	0.455859534555	0.999999998471	Ma08_g20550<br>Ma03_g03560<br>Novel01373<br>Ma03_g00220<br>Ma08_g32940<br>Ma08_g04460<br>Ma10_g03470<br>Ma02_g24010<br>Ma06_g02940<br>Ma11_g15660<br>Ma06_g25950<br>Ma03_g13720<br>Ma11_g23050<br>Ma08_g21900	osa:4343598<br>osa:4330523<br>osa:4339846<br>osa:4331150<br>osa:4324844<br>osa:4340334<br>osa:4330523<br>osa:4340986<br>osa:4327738<br>osa:4348804<br>osa:4334104<br>osa:4331150<br>osa:4344389<br>osa:4344389	4343598<br>4330523<br>4339846<br>4331150<br>4324844<br>4340334<br>4330523<br>4340986<br>4327738<br>4348804<br>4334104<br>4331150<br>4344389<br>4344389		

Circadian rhythm - plant	9	25	0.45988514401	0.999999998471	Ma11_g24610<br>Ma02_g24860<br>Ma10_g20600<br>Ma06_g09780<br>Ma02_g08710<br>Ma01_g01670<br>Ma04_g16680<br>Ma10_g20580<br>Ma10_g20560	osa:4350636<br>osa:4350636<br>osa:4350636<br>osa:4350636<br>osa:4349831<br>osa:4344703<br>osa:4344703<br>osa:4350636<br>osa:4350636	4350636<br>4350636<br>4350636<br>4350636<br>4349831<br>4344703<br>4344703<br>4350636<br>4350636		
Ether lipid metabolism	8	22	0.46058215883	0.999999998471	Ma01_g08090<br>Ma01_g08380<br>Ma10_g26890<br>Ma04_g26360<br>Ma07_g11670<br>Ma10_g11950<br>Ma06_g37750<br>Ma07_g18790	osa:4327647<br>osa:4347745<br>osa:4327647<br>osa:4330302<br>osa:4333862<br>osa:4326465<br>osa:4327647<br>osa:4347745	4327647<br>4347745<br>4327647<br>4330302<br>4333862<br>4326465<br>4327647<br>4347745		
Tropane, piperidine and pyridine alkaloid biosynthesis	6	16	0.461608444493	0.999999998471	Ma09_g03470<br>Ma06_g35980<br>Ma00_g05010<br>Ma10_g16940<br>Ma09_g29570<br>Ma09_g03450	osa:4332375<br>osa:4332375<br>osa:4335335<br>osa:4336153<br>osa:4329101<br>osa:4332375	4332375<br>4332375<br>4335335<br>4336153<br>4329101<br>4332375		
SNARE interactions in vesicular transport	13	38	0.488784767123	0.999999998471	Ma04_g05470<br>Ma10_g29500<br>Ma05_g03860<br>Ma11_g17320<br>Ma01_g05280<br>Ma05_g21960<br>Ma09_g26290<br>Ma09_g00140<br>Ma08_g02910<br>Ma06_g32840<br>Ma07_g16800<br>Ma11_g15620<br>Ma09_g19180	osa:9271327<br>osa:4341413<br>osa:4327713<br>osa:4325070<br>osa:4327678<br>osa:4331172<br>osa:4334384<br>osa:4339549<br>osa:4334356<br>osa:4334356<br>osa:4331172<br>osa:4331172<br>osa:4328159	9271327<br>4341413<br>4327713<br>4325070<br>4327678<br>4331172<br>4334384<br>4339549<br>4334356<br>4334356<br>4331172<br>4331172<br>4328159		
Valine, leucine and isoleucine biosynthesis	5	14	0.514612819123	0.999999998471	Ma06_g24910<br>Ma08_g03130<br>Ma03_g16070<br>Ma06_g30380<br>Ma03_g16060	osa:4339677<br>osa:4333898<br>osa:4339583<br>osa:4329938<br>osa:4332151	4339677<br>4333898<br>4339583<br>4329938<br>4332151		
Glycosaminoglycan degradation	3	8	0.529522374345	0.999999998471	Ma05_g09350<br>Ma06_g01700<br>Ma06_g01670	osa:4339457<br>osa:4324914<br>osa:4338802	4339457<br>4324914<br>4338802		
Riboflavin metabolism	3	8	0.529522374345	0.999999998471	Ma10_g24730<br>Ma04_g23230<br>Ma06_g32150	osa:4329733<br>osa:4348834<br>osa:4329733	4329733<br>4348834<br>4329733		
Glutathione metabolism	26	80	0.535694730501	0.999999998471	Ma08_g05120<br>Ma11_g12290<br>Ma04_g04960<br>Ma02_g04250<br>Ma06_g34840<br>Ma02_g05000<br>Ma09_g26530<br>Ma01_g16820<br>Ma09_g26570<br>Ma11_g18850<br>Ma05_g07440<br>Ma08_g21800<br>Ma10_g09500<br>Ma07_g07290<br>Ma05_g26600<br>Ma08_g29310<br>Ma09_g26490<br>Ma10_g23010<br>Ma11_g17480<br>Ma06_g16830<br>Ma02_g13070<br>Ma03_g14250<br>Ma03_g17510<br>Ma09_g26520<br>Ma06_g38960<br>Ma04_g06200	osa:4342988<br>osa:4325710<br>osa:4325707<br>osa:4325710<br>osa:4332455<br>osa:4347319<br>osa:4346877<br>osa:4349192<br>osa:4346877<br>osa:4326828<br>osa:4325764<br>osa:4332455<br>osa:4346877<br>osa:4336886<br>osa:4336627<br>osa:4329889<br>osa:4346877<br>osa:4337696<br>osa:4351008<br>osa:4324526<br>osa:4331535<br>osa:4346337<br>osa:4339892<br>osa:4346877<br>osa:4336353<br>osa:4342996	4342988<br>4325710<br>4325707<br>4325710<br>4332455<br>4347319<br>4346877<br>4349192<br>4346877<br>4326828<br>4325764<br>4332455<br>4346877<br>4336886<br>4336627<br>4329889<br>4346877<br>4337696<br>4351008<br>4324526<br>4331535<br>4346337<br>4339892<br>4346877<br>4336353<br>4342996		
Phagosome	26	80	0.535694730501	0.999999998471	Ma02_g03320<br>Ma04_g37240<br>Ma04_g13410<br>Ma04_g12700<br>Ma08_g05390<br>Ma07_g16530<br>Ma05_g29040<br>Ma09_g23510<br>Ma07_g03930<br>Ma03_g06030<br>Ma05_g03860	osa:4341810<br>osa:4327731<br>osa:4337284<br>osa:4345097<br>osa:4327833<br>osa:4352862<br>osa:4328420<br>osa:4341751<br>osa:4329132<br>osa:4330693<br>osa:4327713	4341810<br>4327731<br>4337284<br>4345097<br>4327833<br>4352862<br>4328420<br>4341751<br>4329132<br>4330693<br>4327713		

					Ma10_g29910<br>Ma03_g19260<br>Ma09_g26290<br>Ma07_g25660<br>Ma05_g16910<br>Ma07_g20040<br>Ma05_g18380<br>Ma04_g05470<br>Ma09_g13100<br>Ma06_g10030<br>Ma04_g11950<br>Ma06_g06270<br>Ma11_g13680<br>Ma09_g05400<br>Ma10_g06890	osa:4345628<br>osa:4326917<br>osa:4334384<br>osa:4327929<br>osa:4326917<br>osa:4345628<br>osa:4337563<br>osa:9271327<br>osa:4341707<br>osa:4326917<br>osa:4327550<br>osa:4350197<br>osa:4339262<br>osa:4327180<br>osa:4327180	4345628<br>4326917<br>4334384<br>4327929<br>4326917<br>4345628<br>4337563<br>9271327<br>4341707<br>4326917<br>4327550<br>4350197<br>4339262<br>4327180<br>4327180	
Sesquiterpenoid and triterpenoid biosynthesis	2	5	0.542764831954	0.999999998471	Ma08_g04920<br>Ma08_g04900	osa:4332153<br>osa:4332153	4332153<br>4332153	
Tyrosine metabolism	12	37	0.556204777175	0.999999998471	Ma04_g25530<br>Ma08_g29910<br>Ma07_g11500<br>Ma09_g08110<br>Ma00_g05010<br>Ma11_g15760<br>Ma08_g29610<br>Ma10_g16940<br>Ma02_g09950<br>Ma09_g29570<br>Ma04_g31940<br>Ma07_g11510	osa:4331130<br>osa:4350053<br>osa:4331893<br>osa:4350053<br>osa:4335335<br>osa:4343080<br>osa:4329692<br>osa:4336153<br>osa:4350053<br>osa:4329101<br>osa:4330090<br>osa:4331893	4331130<br>4350053<br>4331893<br>4350053<br>4335335<br>4343080<br>4329692<br>4336153<br>4350053<br>4329101<br>4330090<br>4331893	
Galactose metabolism	17	54	0.589734685756	0.999999998471	Ma07_g13520<br>Ma10_g15780<br>Ma07_g01870<br>Ma05_g24340<br>Ma06_g37010<br>Ma06_g28330<br>Ma03_g16650<br>Ma07_g11340<br>Ma08_g15070<br>Ma02_g18360<br>Ma05_g09350<br>Ma10_g15800<br>Ma11_g24070<br>Ma07_g10560<br>Ma06_g10450<br>Ma09_g11280<br>Ma09_g23530	osa:4327301<br>osa:4335789<br>osa:4342703<br>osa:4341967<br>osa:4345925<br>osa:4339812<br>osa:4339812<br>osa:4348582<br>osa:4348230<br>osa:4327301<br>osa:4339457<br>osa:4335789<br>osa:4341824<br>osa:4327301<br>osa:4345925<br>osa:4348138<br>osa:4336928	4327301<br>4335789<br>4342703<br>4341967<br>4345925<br>4339812<br>4339812<br>4348582<br>4348230<br>4327301<br>4339457<br>4335789<br>4341824<br>4327301<br>4345925<br>4348138<br>4336928	
Porphyrin and chlorophyll metabolism	11	35	0.593958950972	0.999999998471	Ma10_g05100<br>Ma06_g06810<br>Ma06_g24480<br>Ma09_g14110<br>Ma06_g09580<br>Ma10_g26330<br>Ma08_g14810<br>Ma06_g23970<br>Ma06_g00500<br>Ma08_g24890<br>Ma04_g31690	osa:4326901<br>osa:4341462<br>osa:4332771<br>osa:4341997<br>osa:4346136<br>osa:4327178<br>osa:4349044<br>osa:4326635<br>osa:4328118<br>osa:4330711<br>osa:4331611	4326901<br>4341462<br>4332771<br>4341997<br>4346136<br>4327178<br>4349044<br>4326635<br>4328118<br>4330711<br>4331611	
Folate biosynthesis	6	19	0.600027438467	0.999999998471	Ma10_g14640<br>Ma09_g31410<br>Ma08_g00920<br>Ma10_g02550<br>Ma08_g25440<br>Ma09_g31420	osa:4331360<br>osa:4347851<br>osa:4337297<br>osa:4326358<br>osa:4328260<br>osa:4347851	4331360<br>4347851<br>4337297<br>4326358<br>4328260<br>4347851	
Limonene and pinene degradation	4	14	0.677447351546	0.999999998471	Ma05_g05010<br>Ma07_g09040<br>Ma07_g06700<br>Ma03_g06120	osa:4340725<br>osa:4341325<br>osa:4349111<br>osa:4340725	4340725<br>4341325<br>4349111<br>4340725	
Tryptophan metabolism	6	21	0.680613005808	0.999999998471	Ma03_g13760<br>Ma03_g10630<br>Ma11_g19670<br>Ma03_g06120<br>Ma05_g05010<br>Ma03_g14690	osa:4334994<br>osa:4351695<br>osa:4334994<br>osa:4340725<br>osa:4340725<br>osa:4328073	4334994<br>4351695<br>4334994<br>4340725<br>4340725<br>4328073	
Synthesis and degradation of ketone bodies	2	7	0.687785698892	0.999999998471	Ma03_g12070<br>Ma04_g10090	osa:4331418<br>osa:4326266	4331418<br>4326266	
Lysine biosynthesis	4	15	0.720869893526	0.999999998471	Ma05_g01700<br>Ma01_g16200<br>Ma06_g12620<br>Ma11_g13600	osa:4346642<br>osa:4332563<br>osa:4334188<br>osa:4332563	4346642<br>4332563<br>4334188<br>4332563	
Photosynthesis - antenna proteins	4	15	0.720869893526	0.999999998471	Ma10_g15370<br>Ma09_g06640<br>Ma04_g07920	osa:4324599<br>osa:4324599<br>osa:4324599	4324599<br>4324599<br>4324599	

					Ma04_g14940	osa:4324599	4324599		
Glycosphingolipid biosynthesis - globo series	2	8	0.744829693731	0.999999998471	Ma06_g01700<br>Ma06_g01670	osa:4324914<br>osa:4338802	4324914<br>4338802		
Arachidonic acid metabolism	3	12	0.750526267163	0.999999998471	Ma06_g16830<br>Ma07_g11670<br>Ma05_g26600	osa:4324526<br>osa:4333862<br>osa:4336627	4324526<br>4333862<br>4336627		
Pyruvate metabolism	21	75	0.761605341575	0.999999998471	Ma10_g21340<br>Ma02_g03830<br>Ma05_g25700<br>Ma04_g35390<br>Ma07_g06750<br>Ma06_g07450<br>Ma11_g01870<br>Ma03_g32890<br>Ma03_g06120<br>Ma10_g25720<br>Ma09_g30880<br>Ma04_g01300<br>Ma05_g05010<br>Ma09_g20570<br>Ma08_g08880<br>Ma06_g28550<br>Ma07_g06760<br>Ma01_g17610<br>Ma06_g21080<br>Ma03_g14310<br>Ma10_g12590	osa:4338007<br>osa:4338750<br>osa:4327423<br>osa:4349454<br>osa:4328012<br>osa:4338161<br>osa:4338324<br>osa:4326769<br>osa:4340725<br>osa:4345657<br>osa:4344858<br>osa:4352871<br>osa:4340725<br>osa:4337406<br>osa:4346159<br>osa:4345387<br>osa:4328012<br>osa:4337406<br>osa:4344858<br>osa:4346332<br>osa:4329518	4338007<br>4338750<br>4327423<br>4349454<br>4328012<br>4338161<br>4338324<br>4326769<br>4340725<br>4345657<br>4344858<br>4352871<br>4340725<br>4337406<br>4346159<br>4345387<br>4328012<br>4337406<br>4344858<br>4346332<br>4329518		
Biotin metabolism	5	20	0.769922665216	0.999999998471	Ma08_g04460<br>Ma06_g01540<br>Ma06_g25950<br>Ma08_g20550<br>Ma04_g25940	osa:4340334<br>osa:4346185<br>osa:4334104<br>osa:4343598<br>osa:4345286	4340334<br>4346185<br>4334104<br>4343598<br>4345286		
Inositol phosphate metabolism	12	45	0.773637394749	0.999999998471	Ma11_g24010<br>Ma03_g27370<br>Ma10_g29910<br>Ma03_g27870<br>Ma05_g31060<br>Ma04_g08270<br>Ma04_g28920<br>Ma09_g24600<br>Ma01_g14940<br>Ma05_g16190<br>Ma10_g11950<br>Ma07_g20040	osa:4328439<br>osa:4347947<br>osa:4345628<br>osa:4347691<br>osa:4325211<br>osa:4341305<br>osa:4342610<br>osa:4347691<br>osa:4331917<br>osa:4331917<br>osa:4326465<br>osa:4345628	4328439<br>4347947<br>4345628<br>4347691<br>4325211<br>4341305<br>4342610<br>4347691<br>4331917<br>4331917<br>4326465<br>4345628		
Pantothenate and CoA biosynthesis	6	24	0.780052862267	0.999999998471	Ma05_g22240<br>Ma06_g24910<br>Ma06_g10670<br>Ma06_g30380<br>Ma03_g16070<br>Ma03_g16060	osa:4343249<br>osa:4339677<br>osa:4347680<br>osa:4329938<br>osa:4339583<br>osa:4332151	4343249<br>4339677<br>4347680<br>4329938<br>4339583<br>4332151		
Phosphatidylinositol signaling system	11	43	0.805201457506	0.999999998471	Ma05_g08710<br>Ma07_g01400<br>Ma11_g24010<br>Ma03_g27370<br>Ma10_g29910<br>Ma03_g02590<br>Ma03_g26590<br>Ma01_g07590<br>Ma07_g20040<br>Ma01_g21300<br>Ma02_g03180	osa:4344344<br>osa:4344344<br>osa:4328439<br>osa:4347947<br>osa:4345628<br>osa:4352615<br>osa:4344344<br>osa:4326903<br>osa:4345628<br>osa:4344344<br>osa:4339172	4344344<br>4344344<br>4328439<br>4347947<br>4345628<br>4352615<br>4344344<br>4326903<br>4345628<br>4344344<br>4339172		
Glycerophospholipid metabolism	18	68	0.814914948312	0.999999998471	Ma07_g10180<br>Ma01_g08090<br>Ma11_g18880<br>Ma03_g02590<br>Ma03_g21800<br>Ma09_g25530<br>Ma05_g29560<br>Ma01_g08380<br>Ma03_g25580<br>Ma01_g00390<br>Ma05_g12640<br>Ma05_g26380<br>Ma07_g11670<br>Ma10_g26890<br>Ma04_g26360<br>Ma06_g37750<br>Ma10_g11950<br>Ma07_g18790	osa:4350917<br>osa:4327647<br>osa:4326817<br>osa:4352615<br>osa:4339554<br>osa:4325633<br>osa:4351119<br>osa:4347745<br>osa:4351119<br>osa:4337344<br>osa:4349006<br>osa:4347160<br>osa:4333862<br>osa:4327647<br>osa:4330302<br>osa:4327647<br>osa:4326465<br>osa:4347745	4350917<br>4327647<br>4326817<br>4352615<br>4339554<br>4325633<br>4351119<br>4347745<br>4351119<br>4337344<br>4349006<br>4347160<br>4333862<br>4327647<br>4330302<br>4327647<br>4326465<br>4347745		
C5-Branched dibasic acid metabolism	1	5	0.814977325051	0.999999998471	Ma06_g30380	osa:4329938	4329938		

Glyoxylate and dicarboxylate metabolism	15	58	0.821874423293	0.999999998471	Ma06_g21190<br>Ma06_g08130<br>Ma07_g22000<br>Ma03_g01840<br>Ma09_g07020<br>Ma04_g01300<br>Ma01_g01610<br>Ma10_g25720<br>Ma07_g15870<br>Ma05_g25700<br>Ma06_g24830<br>Ma05_g21330<br>Ma03_g21060<br>Ma00_g04030<br>Ma03_g14690	osa:4337048<br>osa:4352058<br>osa:4345962<br>osa:4343908<br>osa:4352058<br>osa:4352871<br>osa:4349114<br>osa:4345657<br>osa:4328326<br>osa:4327423<br>osa:3131463<br>osa:4327981<br>osa:4330649<br>osa:4330649<br>osa:4328073	4337048<br>4352058<br>4345962<br>4343908<br>4352058<br>4352871<br>4349114<br>4345657<br>4328326<br>4327423<br>3131463<br>4327981<br>4330649<br>4330649<br>4328073		
Protein processing in endoplasmic reticulum	58	203	0.82635791996	0.999999998471	Ma09_g07430<br>Novel00479<br>Ma01_g14540<br>Ma05_g23160<br>Ma09_g23200<br>Ma10_g21080<br>Ma09_g19460<br>Ma06_g13850<br>Ma01_g14490<br>Ma04_g11590<br>Ma07_g24310<br>Ma10_g26270<br>Ma09_g12320<br>Ma10_g16680<br>Ma10_g20390<br>Ma05_g18410<br>Ma04_g26080<br>Ma07_g19070<br>Ma11_g07190<br>Novel00994<br>Ma10_g11310<br>Ma08_g21660<br>Ma02_g18000<br>Ma09_g00500<br>Ma01_g05370<br>Ma06_g30090<br>Ma08_g34730<br>Ma02_g04060<br>Ma10_g06370<br>Ma08_g14870<br>Ma07_g17020<br>Ma10_g14420<br>Ma08_g02190<br>Ma04_g37750<br>Ma07_g14960<br>Ma08_g21650<br>Ma06_g07350<br>Ma10_g06820<br>Ma03_g29390<br>Ma04_g02540<br>Ma07_g25660<br>Ma11_g22310<br>Ma06_g31680<br>Ma08_g10290<br>Ma09_g26390<br>Ma05_g07260<br>Ma09_g13100<br>Ma09_g00490<br>Ma07_g19020<br>Ma10_g29000<br>Ma04_g12700<br>Ma09_g24030<br>Ma03_g17190<br>Ma11_g13680<br>Ma03_g23000<br>Ma09_g03950<br>Ma01_g10230<br>Ma02_g05870	osa:4351875<br>osa:4329219<br>osa:4332357<br>osa:4336483<br>osa:4345893<br>osa:4325341<br>osa:4342702<br>osa:4351875<br>osa:4332361<br>osa:4332360<br>osa:4334781<br>osa:4332538<br>osa:4346639<br>osa:4332237<br>osa:4326152<br>osa:4335881<br>osa:4337331<br>osa:4328114<br>osa:4336483<br>osa:4342827<br>osa:4350180<br>osa:4351208<br>osa:4351208<br>osa:4332361<br>osa:4324566<br>osa:4351875<br>osa:4339012<br>osa:4326806<br>osa:9267802<br>osa:4327971<br>osa:4335701<br>osa:4338088<br>osa:4332363<br>osa:4333580<br>osa:4332391<br>osa:4351208<br>osa:4328940<br>osa:4328940<br>osa:4345893<br>osa:4328940<br>osa:4327929<br>osa:4339013<br>osa:4333580<br>osa:4327708<br>osa:4333580<br>osa:4337331<br>osa:4341707<br>osa:4332360<br>osa:4342077<br>osa:4334781<br>osa:4345097<br>osa:4331645<br>osa:4328940<br>osa:4339262<br>osa:4350002<br>osa:4351208<br>osa:4350002<br>osa:4336483	4351875<br>4329219<br>4332357<br>4336483<br>4345893<br>4325341<br>4342702<br>4351875<br>4332361<br>4332360<br>4334781<br>4332538<br>4346639<br>4332237<br>4326152<br>4335881<br>4337331<br>4328114<br>4336483<br>4342827<br>4350180<br>4351208<br>4351208<br>4332361<br>4324566<br>4351875<br>4339012<br>4326806<br>9267802<br>4327971<br>4335701<br>4338088<br>4332363<br>4333580<br>4332391<br>4351208<br>4328940<br>4328940<br>4345893<br>4328940<br>4327929<br>4339013<br>4333580<br>4327708<br>4333580<br>4337331<br>4341707<br>4332360<br>4342077<br>4334781<br>4345097<br>4331645<br>4328940<br>4339262<br>4350002<br>4351208<br>4350002<br>4336483		
Regulation of autophagy	6	26	0.832074498419	0.999999998471	Ma05_g15170<br>Ma11_g23590<br>Ma03_g20520<br>Ma11_g05750<br>Ma02_g07930<br>Ma06_g27130	osa:4336527<br>osa:4349525<br>osa:4325756<br>osa:4325756<br>osa:4334090<br>osa:4336527	4336527<br>4349525<br>4325756<br>4325756<br>4334090<br>4336527		
One carbon pool by folate	3	15	0.854536604203	0.999999998471	Ma06_g08130<br>Ma06_g21100<br>Ma09_g07020	osa:4352058<br>osa:4337051<br>osa:4352058	4352058<br>4337051<br>4352058		
N-Glycan biosynthesis	8	35	0.861321660802	0.999999998471	Ma04_g39490<br>Ma07_g17020<br>Ma10_g14420	osa:4331021<br>osa:4335701<br>osa:4338088	4331021<br>4335701<br>4338088		

					Ma05_g10890<br>Ma09_g19460<br>Ma05_g23550<br>Ma06_g12590<br>Ma08_g29090	osa:4334626<br>osa:4342702<br>osa:4344180<br>osa:4352649<br>osa:4336162	4334626<br>4342702<br>4344180<br>4352649<br>4336162		
Fatty acid metabolism	18	72	0.870285548073	0.999999998471	Ma08_g20550<br>Ma03_g03560<br>Ma03_g00220<br>Novel01373<br>Ma08_g32940<br>Ma08_g04460<br>Ma10_g03470<br>Ma04_g25940<br>Ma02_g24010<br>Ma09_g29260<br>Ma06_g02940<br>Ma08_g30510<br>Ma11_g15660<br>Ma02_g10050<br>Ma06_g25950<br>Ma03_g13720<br>Ma11_g23050<br>Ma08_g21900	osa:4343598<br>osa:4330523<br>osa:4331150<br>osa:4339846<br>osa:4324844<br>osa:4340334<br>osa:4330523<br>osa:4345286<br>osa:4340986<br>osa:4340093<br>osa:4327738<br>osa:4344267<br>osa:4348804<br>osa:4351610<br>osa:4334104<br>osa:4331150<br>osa:4344389<br>osa:4344389	4343598<br>4330523<br>4331150<br>4339846<br>4324844<br>4340334<br>4330523<br>4345286<br>4340986<br>4340093<br>4327738<br>4344267<br>4348804<br>4351610<br>4334104<br>4331150<br>4344389<br>4344389		
Fatty acid elongation	5	24	0.873077299041	0.999999998471	Ma10_g28390<br>Ma04_g32180<br>Ma07_g08400<br>Ma09_g06710<br>Ma04_g22350	osa:4341445<br>osa:4332236<br>osa:4341445<br>osa:4339645<br>osa:4332236	4341445<br>4332236<br>4341445<br>4339645<br>4332236		
Isoquinoline alkaloid biosynthesis	4	20	0.874496623126	0.999999998471	Ma09_g29570<br>Ma11_g15760<br>Ma10_g16940<br>Ma00_g05010	osa:4329101<br>osa:4343080<br>osa:4336153<br>osa:4335335	4329101<br>4343080<br>4336153<br>4335335		
Butanoate metabolism	4	20	0.874496623126	0.999999998471	Ma03_g12070<br>Ma04_g10090<br>Ma04_g37810<br>Ma06_g30380	osa:4331418<br>osa:4326266<br>osa:4333932<br>osa:4329938	4331418<br>4326266<br>4333932<br>4329938		
Selenocompound metabolism	3	16	0.879599112455	0.999999998471	Ma04_g28070<br>Ma08_g27570<br>Ma01_g07600	osa:4352833<br>osa:4334073<br>osa:4334073	4352833<br>4334073<br>4334073		
Taurine and hypotaurine metabolism	3	16	0.879599112455	0.999999998471	Ma04_g37810<br>Ma06_g16830<br>Ma05_g06070	osa:4333932<br>osa:4324526<br>osa:4339793	4333932<br>4324526<br>4339793		
Non-homologous end-joining	1	7	0.894593071931	0.999999998471	Ma04_g11140	osa:4334058	4334058		
2-Oxocarboxylic acid metabolism	10	46	0.909844060255	0.999999998471	Novel00760<br>Ma06_g12620<br>Ma06_g24910<br>Ma09_g07280<br>Ma05_g15910<br>Ma06_g30380<br>Ma11_g23060<br>Ma03_g16060<br>Ma03_g16070<br>Ma06_g38960	osa:4324442<br>osa:4334188<br>osa:4339677<br>osa:4329858<br>osa:4333177<br>osa:4329938<br>osa:4343164<br>osa:4332151<br>osa:4339583<br>osa:4336353	4324442<br>4334188<br>4339677<br>4329858<br>4333177<br>4329938<br>4343164<br>4332151<br>4339583<br>4336353		
Nicotinate and nicotinamide metabolism	2	13	0.913616942477	0.999999998471	Ma03_g23860<br>Ma04_g11720	osa:4346302<br>osa:4347802	4346302<br>4347802		
Vitamin B6 metabolism	1	8	0.920443993561	0.999999998471	Ma08_g32190	osa:4342162	4342162		
Terpenoid backbone biosynthesis	10	47	0.920636693493	0.999999998471	Ma03_g12070<br>Ma10_g17260<br>Ma01_g17990<br>Ma05_g14980<br>Ma03_g26140<br>Ma08_g04770<br>Ma10_g03310<br>Ma05_g05920<br>Ma08_g24890<br>Ma04_g36760	osa:4331418<br>osa:4343523<br>osa:4343721<br>osa:4342614<br>osa:4338768<br>osa:4334003<br>osa:4327435<br>osa:4340090<br>osa:4330711<br>osa:4340251	4331418<br>4343523<br>4343721<br>4342614<br>4338768<br>4334003<br>4327435<br>4340090<br>4330711<br>4340251		
Fatty acid biosynthesis	8	40	0.929561563742	0.999999998471	Ma08_g20550<br>Ma08_g32940<br>Ma08_g04460<br>Ma04_g25940<br>Ma09_g29260<br>Ma06_g02940<br>Ma06_g25950<br>Ma02_g10050	osa:4343598<br>osa:4324844<br>osa:4340334<br>osa:4345286<br>osa:4340093<br>osa:4327738<br>osa:4334104<br>osa:4351610	4343598<br>4324844<br>4340334<br>4345286<br>4340093<br>4327738<br>4334104<br>4351610		
Sulfur relay system	2	14	0.931202210704	0.999999998471	Ma08_g25440<br>Ma09_g01760	osa:4328260<br>osa:4329515	4328260<br>4329515		
Protein export	12	56	0.932416741078	0.999999998471	Ma09_g26120<br>Ma09_g13100<br>Ma04_g12700	osa:4324712<br>osa:4341707<br>osa:4345097	4324712<br>4341707<br>4345097		

					Novel00479<br>Ma04_g27690<br>Ma02_g14460<br>Ma01_g07050<br>Ma07_g25660<br>Ma07_g08190<br>Ma10_g28590<br>Ma09_g03980<br>Ma10_g25180	osa:4329219<br>osa:4333514<br>osa:4333514<br>osa:4352133<br>osa:4327929<br>osa:4347239<br>osa:4347239<br>osa:4324524<br>osa:4324904	4329219<br>4333514<br>4333514<br>4352133<br>4327929<br>4347239<br>4347239<br>4324524<br>4324904		
Alanine, aspartate and glutamate metabolism	8	41	0.938992046977	0.999999998471	Ma07_g22000<br>Ma10_g18940<br>Ma02_g12240<br>Ma03_g27120<br>Ma04_g37810<br>Ma03_g21060<br>Ma00_g04030<br>Ma08_g28590	osa:4345962<br>osa:4331779<br>osa:4330164<br>osa:4326346<br>osa:4333932<br>osa:4330649<br>osa:4330649<br>osa:4324398	4345962<br>4331779<br>4330164<br>4326346<br>4333932<br>4330649<br>4330649<br>4324398		
Mismatch repair	8	42	0.947294033432	0.999999998471	Ma00_g02630<br>Ma09_g22290<br>Ma03_g03030<br>Ma03_g01100<br>Ma05_g26770<br>Ma07_g18410<br>Ma07_g04480<br>Ma08_g16890	osa:4327706<br>osa:4326564<br>osa:4326359<br>osa:4326359<br>osa:4325353<br>osa:4337442<br>osa:4331062<br>osa:4334389	4327706<br>4326564<br>4326359<br>4326359<br>4325353<br>4337442<br>4331062<br>4334389		
Ubiquitin mediated proteolysis	28	119	0.953260347946	0.999999998471	Ma01_g23000<br>Ma01_g22700<br>Ma11_g19850<br>Ma09_g16250<br>Ma06_g19950<br>Ma09_g12320<br>Ma07_g14460<br>Novel00180<br>Ma04_g26080<br>Ma07_g19070<br>Ma10_g26170<br>Ma09_g00210<br>Ma01_g23720<br>Ma03_g05450<br>Ma06_g31510<br>Ma10_g20390<br>Novel01258<br>Ma01_g22160<br>Ma06_g28570<br>Ma06_g07350<br>Ma10_g06820<br>Ma04_g02540<br>Ma01_g19370<br>Ma11_g22310<br>Ma08_g12680<br>Ma08_g00590<br>Ma03_g17190<br>Ma05_g07260	osa:4330430<br>osa:4336870<br>osa:4331448<br>osa:4344172<br>osa:4338850<br>osa:4346639<br>osa:4329066<br>osa:4326397<br>osa:4337331<br>osa:4328114<br>osa:4329066<br>osa:4324290<br>osa:4330430<br>osa:4331446<br>osa:4333728<br>osa:4326152<br>osa:4330430<br>osa:4330430<br>osa:4325905<br>osa:4328940<br>osa:4328940<br>osa:4328940<br>osa:4330430<br>osa:4339013<br>osa:4348796<br>osa:4327237<br>osa:4328940<br>osa:4337331	4330430<br>4336870<br>4331448<br>4344172<br>4338850<br>4346639<br>4329066<br>4326397<br>4337331<br>4328114<br>4329066<br>4324290<br>4330430<br>4331446<br>4333728<br>4326152<br>4330430<br>4330430<br>4325905<br>4328940<br>4328940<br>4328940<br>4330430<br>4339013<br>4348796<br>4327237<br>4328940<br>4337331		
Diterpenoid biosynthesis	4	27	0.965093654715	0.999999998471	Ma03_g28580<br>Ma02_g01570<br>Ma10_g20860<br>Ma10_g21550	osa:4339885<br>osa:4334841<br>osa:4336960<br>osa:4325145	4339885<br>4334841<br>4336960<br>4325145		
Citrate cycle (TCA cycle)	9	50	0.970263586407	0.999999998471	Novel00760<br>Ma05_g25700<br>Ma11_g21050<br>Ma10_g25720<br>Ma08_g08880<br>Ma04_g01300<br>Ma08_g06070<br>Ma06_g38960<br>Ma09_g07280	osa:4324442<br>osa:4327423<br>osa:4330016<br>osa:4345657<br>osa:4346159<br>osa:4352871<br>osa:4352549<br>osa:4336353<br>osa:4329858	4324442<br>4327423<br>4330016<br>4345657<br>4346159<br>4352871<br>4352549<br>4336353<br>4329858		
Lysine degradation	3	25	0.981067811724	0.999999998471	Ma05_g05010<br>Ma05_g29990<br>Ma03_g06120	osa:4340725<br>osa:4339168<br>osa:4340725	4340725<br>4339168<br>4340725		
RNA polymerase	6	44	0.991457022413	0.999999998471	Ma07_g05720<br>Ma06_g27690<br>Ma10_g17250<br>Ma04_g20190<br>Ma10_g18010<br>Ma10_g21000	osa:4352511<br>osa:4351671<br>osa:4342517<br>osa:4331295<br>osa:4333888<br>osa:4325336	4352511<br>4351671<br>4342517<br>4331295<br>4333888<br>4325336		
RNA degradation	17	92	0.991470079071	0.999999998471	Ma09_g30300<br>Ma01_g03570<br>Ma09_g00960<br>Ma11_g06010<br>Ma07_g17220<br>Ma03_g23620<br>Ma10_g13820<br>Ma07_g05920<br>Ma09_g17850	osa:4337457<br>osa:4340042<br>osa:4336331<br>osa:4339910<br>osa:4331095<br>osa:4325074<br>osa:4325469<br>osa:4331379<br>osa:4339910	4337457<br>4340042<br>4336331<br>4339910<br>4331095<br>4325074<br>4325469<br>4331379<br>4339910		

					Ma05_g05050<br>Ma03_g28660<br>Ma01_g00690<br>Ma11_g08900<br>Ma02_g07050<br>Ma11_g20920<br>Ma10_g09070<br>Novel01023	osa:4340042<br>osa:4339910<br>osa:4331006<br>osa:4335963<br>osa:4331006<br>osa:4348176<br>osa:4348824<br>osa:4349826	4340042<br>4339910<br>4331006<br>4335963<br>4331006<br>4348176<br>4348824<br>4349826		
Purine metabolism	27	135	0.994131692559	0.999999998471	Ma02_g13970<br>Ma03_g05860<br>Ma06_g30180<br>Ma04_g10990<br>Ma07_g24940<br>Ma07_g05720<br>Ma04_g39070<br>Ma03_g23680<br>Ma06_g27690<br>Ma04_g20190<br>Ma08_g15070<br>Ma10_g13290<br>Ma06_g17200<br>Ma03_g03600<br>Ma05_g18090<br>Ma05_g20350<br>Ma05_g01630<br>Ma10_g17250<br>Ma04_g19680<br>Ma00_g02390<br>Ma04_g35390<br>Ma08_g27570<br>Ma10_g18010<br>Ma09_g20570<br>Ma10_g21000<br>Ma01_g17610<br>Ma01_g07600	osa:4328168<br>osa:4329701<br>osa:4350358<br>osa:4343681<br>osa:4344470<br>osa:4352511<br>osa:4352670<br>osa:4324793<br>osa:4351671<br>osa:4331295<br>osa:4348230<br>osa:4344386<br>osa:4344386<br>osa:4328168<br>osa:4344854<br>osa:4334308<br>osa:4352670<br>osa:4342517<br>osa:4333020<br>osa:4344315<br>osa:4349454<br>osa:4334073<br>osa:4333888<br>osa:4337406<br>osa:4325336<br>osa:4337406<br>osa:4334073	4328168<br>4329701<br>4350358<br>4343681<br>4344470<br>4352511<br>4352670<br>4324793<br>4351671<br>4331295<br>4348230<br>4344386<br>4344386<br>4328168<br>4344854<br>4334308<br>4352670<br>4342517<br>4333020<br>4344315<br>4349454<br>4334073<br>4333888<br>4337406<br>4325336<br>4337406<br>4334073		
Oxidative phosphorylation	29	144	0.994874413158	0.999999998471	Ma09_g19700<br>Ma04_g37240<br>mito3_g00150<br>Novel01453<br>Ma06_g04550<br>Ma08_g05390<br>Ma05_g02000<br>Ma03_g04410<br>Ma10_g09000<br>Ma03_g23630<br>Ma09_g23510<br>Ma03_g29190<br>Ma11_g10950<br>Ma04_g13410<br>Ma05_g04210<br>Ma09_g30540<br>Ma04_g31640<br>Ma04_g13500<br>Ma01_g17210<br>Ma09_g26790<br>Ma10_g29140<br>Ma07_g22370<br>Ma02_g07140<br>Ma05_g23990<br>Ma05_g18380<br>Ma03_g21780<br>Ma05_g16030<br>Ma05_g08470<br>Ma02_g06820	osa:4345078<br>osa:4327731<br>osa:6450138<br>osa:6450140<br>osa:4341645<br>osa:4327833<br>osa:4334276<br>osa:4337257<br>osa:4331914<br>osa:4338911<br>osa:4341751<br>osa:4337257<br>osa:4344095<br>osa:4337284<br>osa:4337257<br>osa:4349859<br>osa:4343366<br>osa:4324151<br>osa:4330737<br>osa:4330852<br>osa:4339127<br>osa:4340298<br>osa:4341645<br>osa:4329497<br>osa:4337563<br>osa:4339546<br>osa:4343288<br>osa:4334266<br>osa:4337257	4345078<br>4327731<br>6450138<br>6450140<br>4341645<br>4327833<br>4334276<br>4337257<br>4331914<br>4338911<br>4341751<br>4337257<br>4344095<br>4337284<br>4337257<br>4349859<br>4343366<br>4324151<br>4330737<br>4330852<br>4339127<br>4340298<br>4341645<br>4329497<br>4337563<br>4339546<br>4343288<br>4334266<br>4337257		
Nucleotide excision repair	11	70	0.994884054687	0.999999998471	Ma00_g02630<br>Ma02_g00580<br>Ma03_g03030<br>Ma03_g01100<br>Ma10_g00880<br>Ma10_g20390<br>Ma07_g04480<br>Ma02_g24270<br>Ma09_g16500<br>Ma08_g16890<br>Ma10_g01030	osa:4327706<br>osa:4331991<br>osa:4326359<br>osa:4326359<br>osa:4331991<br>osa:4326152<br>osa:4331062<br>osa:4340268<br>osa:4338689<br>osa:4334389<br>osa:4331991	4327706<br>4331991<br>4326359<br>4326359<br>4331991<br>4326152<br>4331062<br>4340268<br>4338689<br>4334389<br>4331991		
Photosynthesis	13	79	0.995057712848	0.999999998471	Ma01_g17210<br>Ma06_g30010<br>Ma07_g24600<br>Ma04_g22620<br>Ma01_g04250<br>Ma01_g07340<br>Ma01_g10240<br>Ma09_g25040<br>Ma05_g20780<br>Ma04_g31640	osa:4330737<br>osa:4344539<br>osa:4324933<br>osa:4339874<br>osa:4332745<br>osa:4339593<br>osa:4324933<br>osa:4343570<br>osa:4333746<br>osa:4343366	4330737<br>4344539<br>4324933<br>4339874<br>4332745<br>4339593<br>4324933<br>4343570<br>4333746<br>4343366		

					Ma06_g22930<br>Ma09_g31020<br>Ma00_g01500	osa:3131406<br>osa:4344899<br>osa:4351694	3131406<br>4344899<br>4351694		
Base excision repair	5	43	0.996075565534	0.999999998471	Ma00_g04410<br>Ma09_g18880<br>Ma07_g04480<br>Ma09_g08730<br>Ma03_g20210	osa:4340610<br>osa:4327631<br>osa:4331062<br>osa:4329546<br>osa:9268162	4340610<br>4327631<br>4331062<br>4329546<br>9268162		
Basal transcription factors	4	41	0.998089655094	0.999999998471	Ma02_g24270<br>Ma07_g14950<br>Ma09_g16500<br>Ma08_g04080	osa:4340268<br>osa:4338303<br>osa:4338689<br>osa:4338489	4340268<br>4338303<br>4338689<br>4338489		
DNA replication	6	54	0.998776823359	0.999999998471	Ma00_g02630<br>Ma09_g22290<br>Ma03_g03030<br>Ma03_g01100<br>Ma07_g04480<br>Ma08_g16890	osa:4327706<br>osa:4326564<br>osa:4326359<br>osa:4326359<br>osa:4331062<br>osa:4334389	4327706<br>4326564<br>4326359<br>4326359<br>4331062<br>4334389		
Pyrimidine metabolism	18	112	0.999090794187	0.999999998471	Ma07_g25460<br>Ma05_g22240<br>Ma03_g23860<br>Ma07_g05720<br>Ma06_g22860<br>Ma03_g05860<br>Ma11_g14310<br>Ma10_g17250<br>Ma04_g20190<br>Ma10_g18010<br>Ma08_g34680<br>Ma10_g21000<br>Ma03_g26340<br>Ma00_g02390<br>Ma03_g33280<br>Ma06_g27690<br>Ma03_g27120<br>Ma04_g18630	osa:4325937<br>osa:4343249<br>osa:4346302<br>osa:4352511<br>osa:4345970<br>osa:4329701<br>osa:4326794<br>osa:4342517<br>osa:4331295<br>osa:4333888<br>osa:4328991<br>osa:4325336<br>osa:4339675<br>osa:4344315<br>osa:4339675<br>osa:4351671<br>osa:4326346<br>osa:4330910	4325937<br>4343249<br>4346302<br>4352511<br>4345970<br>4329701<br>4326794<br>4342517<br>4331295<br>4333888<br>4328991<br>4325336<br>4339675<br>4344315<br>4339675<br>4351671<br>4326346<br>4330910		
RNA transport	27	151	0.999198397886	0.999999998471	Novel00268<br>Ma10_g22060<br>Ma08_g25720<br>Ma07_g06260<br>Ma07_g25820<br>Ma05_g05900<br>Ma08_g25330<br>Ma10_g26270<br>Ma11_g21440<br>Ma10_g22900<br>Ma11_g11420<br>Ma01_g08110<br>Ma03_g05450<br>Ma05_g06180<br>Ma05_g30450<br>Ma11_g00380<br>Ma10_g17580<br>Ma10_g13420<br>Ma09_g00960<br>Ma11_g01580<br>Ma06_g16850<br>Ma02_g15900<br>Ma03_g29330<br>Ma01_g23590<br>Novel00994<br>Ma06_g03260<br>Ma07_g21170	osa:9272123<br>osa:4328994<br>osa:4346861<br>osa:4346657<br>osa:4329091<br>osa:4325312<br>osa:9272123<br>osa:4332538<br>osa:4343565<br>osa:4331813<br>osa:4345234<br>osa:4343445<br>osa:4331446<br>osa:4328286<br>osa:4338508<br>osa:4352748<br>osa:4343885<br>osa:4331813<br>osa:4336331<br>osa:4339487<br>osa:4332426<br>osa:4343445<br>osa:4329958<br>osa:4328129<br>osa:4342827<br>osa:4325312<br>osa:4341966	9272123<br>4328994<br>4346861<br>4346657<br>4329091<br>4325312<br>9272123<br>4332538<br>4343565<br>4331813<br>4345234<br>4343445<br>4331446<br>4328286<br>4338508<br>4352748<br>4343885<br>4331813<br>4336331<br>4339487<br>4332426<br>4343445<br>4329958<br>4328129<br>4342827<br>4325312<br>4341966		
Homologous recombination	5	53	0.999517955441	0.999999998471	Ma03_g01100<br>Ma10_g18640<br>Ma00_g02630<br>Ma09_g22290<br>Ma03_g03030	osa:4326359<br>osa:4350923<br>osa:4327706<br>osa:4326564<br>osa:4326359	4326359<br>4350923<br>4327706<br>4326564<br>4326359		
Ribosome biogenesis in eukaryotes	10	90	0.999937688376	0.999999998471	Ma06_g24800<br>Ma08_g09760<br>Ma11_g00580<br>Ma06_g03260<br>Ma03_g03000<br>Ma02_g19150<br>Ma05_g05900<br>Ma04_g29900<br>Novel01023<br>Ma07_g17880	osa:4343836<br>osa:4332840<br>osa:4344029<br>osa:4325312<br>osa:4334688<br>osa:4333588<br>osa:4325312<br>osa:4329057<br>osa:4349826<br>osa:4342225	4343836<br>4332840<br>4344029<br>4325312<br>4334688<br>4333588<br>4325312<br>4329057<br>4349826<br>4342225		
mRNA surveillance pathway	12	107	0.999982676481	0.999999998471	Ma05_g06260<br>Ma00_g01270<br>Ma04_g35150<br>Ma08_g20050<br>Ma09_g00960<br>Ma09_g29350	osa:4328769<br>osa:4337821<br>osa:4334494<br>osa:4334494<br>osa:4336331<br>osa:4328733	4328769<br>4337821<br>4334494<br>4334494<br>4336331<br>4328733		

					Ma03_g10420<br>Ma06_g16850<br>Ma11_g23760<br>Ma03_g02170<br>Ma11_g12380<br>Ma04_g31750	osa:4347363<br>osa:4332426<br>osa:4326473<br>osa:4350986<br>osa:4326473<br>osa:4332369	4347363<br>4332426<br>4326473<br>4350986<br>4326473<br>4332369		
Proteasome	4	64	0.999990567681	0.999999998471	Ma06_g03710<br>Novel01107<br>Ma10_g16670<br>Ma07_g23430	osa:4340253<br>osa:4344373<br>osa:4336206<br>osa:4329182	4340253<br>4344373<br>4336206<br>4329182		
Spliceosome	28	196	0.999997933999	0.999999998471	Ma09_g07430<br>Ma06_g13850<br>Ma07_g00810<br>Ma02_g18000<br>Ma09_g12480<br>Ma09_g14000<br>Ma04_g03360<br>Ma07_g06260<br>Ma07_g20610<br>Ma05_g24890<br>Ma08_g10020<br>Ma08_g21660<br>Novel01291<br>Ma05_g17410<br>Ma01_g10610<br>Novel00736<br>Ma08_g34730<br>Ma09_g12490<br>Ma06_g30090<br>Ma06_g16850<br>Ma09_g15600<br>Ma03_g23620<br>Ma06_g21110<br>Ma06_g14060<br>Ma05_g17110<br>Ma08_g21650<br>Ma04_g17280<br>Ma09_g03950	osa:4351875<br>osa:4351875<br>osa:4347985<br>osa:4351208<br>osa:4345910<br>osa:4336619<br>osa:4352279<br>osa:4346657<br>osa:4337147<br>osa:4329810<br>osa:4332781<br>osa:4351208<br>osa:4333866<br>osa:4340964<br>osa:4328900<br>osa:4343339<br>osa:4339012<br>osa:4345254<br>osa:4351875<br>osa:4332426<br>osa:4329902<br>osa:4325074<br>osa:4328237<br>osa:4346506<br>osa:4348949<br>osa:4351208<br>osa:4333915<br>osa:4351208	4351875<br>4351875<br>4347985<br>4351208<br>4345910<br>4336619<br>4352279<br>4346657<br>4337147<br>4329810<br>4332781<br>4351208<br>4333866<br>4340964<br>4328900<br>4343339<br>4339012<br>4345254<br>4351875<br>4332426<br>4329902<br>4325074<br>4328237<br>4346506<br>4348949<br>4351208<br>4333915<br>4351208		
Aminoacyl-tRNA biosynthesis	6	114	0.999999998471	0.999999998471	Ma08_g32300<br>Ma06_g20600<br>Ma05_g24170<br>Ma06_g00500<br>Novel01353<br>Ma00_g01370	osa:4325717<br>osa:4325717<br>osa:4348825<br>osa:4328118<br>osa:4346920<br>osa:4352065	4325717<br>4325717<br>4348825<br>4328118<br>4346920<br>4352065		
